# PsyAcoustX: A flexible MATLAB^®^ package for psychoacoustics research

**DOI:** 10.3389/fpsyg.2015.01498

**Published:** 2015-10-12

**Authors:** Gavin M. Bidelman, Skyler G. Jennings, Elizabeth A. Strickland

**Affiliations:** ^1^Institute for Intelligent Systems, University of Memphis, MemphisTN, USA; ^2^School of Communication Sciences and Disorders, University of Memphis, MemphisTN, USA; ^3^Department of Communication Sciences and Disorders, University of Utah, Salt Lake CityUT, USA; ^4^Department of Speech, Language, and Hearing Sciences, Purdue University, West LafayetteIN, USA

**Keywords:** experiment design software, psychoacoustics, psychometric, auditory perception, forward masking, temporal modulation, temporal effect, gap detection

## Abstract

The demands of modern psychophysical studies require precise stimulus delivery and flexible platforms for experimental control. Here, we describe PsyAcoustX, a new, freely available suite of software tools written in the MATLAB^®^ environment to conduct psychoacoustics research on a standard PC. PsyAcoustX provides a flexible platform to generate and present auditory stimuli in real time and record users’ behavioral responses. Data are automatically logged by stimulus condition and aggregated in an exported spreadsheet for oﬄine analysis. Detection thresholds can be measured adaptively under basic and complex auditory masking tasks and other paradigms (e.g., amplitude modulation detection) within minutes. The flexibility of the module offers experimenters access to nearly every conceivable combination of stimulus parameters (e.g., probe-masker relations). Example behavioral applications are highlighted including the measurement of audiometric thresholds, basic simultaneous and non-simultaneous (i.e., forward and backward) masking paradigms, gap detection, and amplitude modulation detection. Examples of these measurements are provided including the psychoacoustic phenomena of temporal overshoot, psychophysical tuning curves, and temporal modulation transfer functions. Importantly, the core design of PsyAcoustX is easily modifiable, allowing users the ability to adapt its basic structure and create additional modules for measuring discrimination/detection thresholds for other auditory attributes (e.g., pitch, intensity, etc.) or binaural paradigms.

## Introduction

Modern psychoacoustics provides a variety of experimental methodologies to probe the auditory perceptual system with the goal of establishing a link between physical stimuli and their corresponding percepts/sensations. Controlled stimulus manipulations are used to parametrically evaluate the performance of the sensory–perceptual system. Psychoacoustic studies typically involve the precise measurement of detection/discrimination thresholds that approach the limits of listeners’ hearing sensitivity. As such, auditory behavioral experiments have historically required dedicated, highly precise hardware for stimulus delivery and response collection. However, with the ubiquity and flexibility of modern computing, flexible PC-based platforms are now available for the control of rigorous auditory perceptual experiments.

Several commercial software platforms are currently available for auditory research. Both *E-prime*^®^ (Psychology Software Tools, Inc.)^1^ and *Presentation*^®^ (Neurobehavioral Systems, Inc.)^2^ are widely used suites for neuropsychological research. However, both of these packages are available only on the Windows^®^ platform and contain no source code. These platforms are also limited to experimental designs with simple presentation paradigms; auditory stimuli must be pre-made or rendered in external software and then imported as WAV files. This shortcoming makes it cumbersome to implement adaptive tracking rules and forced choice procedures and does not allow the measurement of auditory thresholds—both common in psychoacoustics research (Green and Swets, 1966; Levitt, 1971; Macmillan and Creelman, 2005).

A handful of free software packages are now available for auditory behavioral research. Most of these packages, including WhisPER (Ciba et al., 2009) are tailored only to perceptual audio evaluation (e.g., for sound engineers) and collect only subjective listener judgments. Of the psychophysical packages implementing quantitative response collection, Psycon^3^ is based on the Auditory Syntax (AUX) scripting language (Kwon, 2011) and requires some background programming to run. The platform-independent program PsychoPy (Peirce, 2007), although largely geared toward visual psychophysics research, is able to generate and present auditory stimuli. However, this platform requires some user knowledge of the Python programming language and external auxiliary libraries to handle its graphical and input/output (I/O) engines. Other packages, (MLP Toolbox; Grassi and Soranzo, 2009) ^4^ provide access to psychoacoustic paradigms but limit the user to selected or built-in tasks. Moreover, task modifications require alterations to the source code, rather than through the convenience of a graphical user interface (GUI). Perhaps the most widely used, freely available package is the Psychtoolbox^5^ (Brainard, 1997). The Psychtoolbox is a collection of scripts implemented in MATLAB^®^ (The MathWorks, Natick, MA, USA) that provides access to hardware interfaces (e.g., monitor and sound card), millisecond timing, low-latency audio, and carries a large community of users and support forums. While the Psychtoolbox has garnered a vibrant history of development and is widely used by both behavioral and cognitive neuroscientists, psychoacoustic applications are limited.

Here, we present a new, point-and-click application able to execute typical psychoacoustic paradigms including a wide variety of forward/simultaneous masking paradigms, auditory detection tasks, and temporal processing measures. PsyAcoustX is a freely available, open source platform for psychoacoustics research that uses a common GUI and adaptive tracking rules to measure behavioral thresholds for various psychoacoustic phenomena^6^. PsyAcoustX can be run entirely via its extensive GUI, which provides full access to stimulus generation, calibration, subject logging, and data file I/O. As such, it requires little to no overhead of background programming knowledge. Nevertheless, PsyAcoustX was developed under the MATLAB^®^ programming language to allow maximum flexibility and the development of extensions to the base package by the end user. We first discuss software/hardware system requirements, calibration, and provide an overview of PsyAcoustX’s GUI interface. We then highlight several applications of the program’s engine and illustrate typical data that can be obtained from current modules available in PsyAcoustX.

## Materials and Methods

### Hardware Requirements

PsyAcoustX requires only the MATLAB^®^ base license and the functions of the Signal Processing Toolbox to run. Auditory stimuli are generated as digital waveforms within MATLAB^®^ and output through the PC’s native soundcard and corresponding headphone port. In our laboratories, we use the pro-audio LynxTWO soundcard (Lynx Studio Technology, Inc.) and either ER-2 (Etymotic Research) or E-A-RTONE-5A headphones (Aearo Corp.). Other headphone and soundcard arrangements are possible assuming the user adequately accounts for the frequency response of the signal chain (e.g., correcting for any frequency shaping produced by headphones). However, a soundcard with quantization bit depth of 24-bits is recommended to take advantage of the largest possible dynamic range.

PsyAcoustX was designed in MATLAB^®^ 2011 and has been tested for compatibility through the 2015a release. Data presented herein and those published in our previous studies (Bidelman and Syed Khaja, 2014; Bidelman et al., 2014; Roverud and Strickland, 2015) were collected using MATLAB^®^ version 2013b, or earlier. Importantly, the timing of stimulus presentation in the PsyAcoustX GUI is not dependent on CPU speed. This was made possible by generating stimuli in single experimental trials as contiguous digital waveforms (e.g., see **Figure 3**). This approach has made it possible to create temporally precise auditory stimuli with millisecond resolution—e.g., as would be necessary for a gap detection threshold (GDT) paradigm (Fitzgibbons, 1983; Florentine et al., 1999)—that would be impossible to implement in the presence of any lag/jitter from the CPU. While there are no specific minimum PC hardware requirements for PsyAcoustX, 4 GB of memory is recommended to provide ample space to properly load stimuli to RAM during runtime execution and allow maximum fluidity of the program’s GUI.

### Calibration

PsyAcoustX has a dedicated function which allows the user to calibrate the system output. Detailed calibration procedures are provided in the manual accompanying PsyAcoustX’s source code (see Supplementary Material). Briefly, the GUI allows the user to calibrate the system in reference to a 10-s, 1-kHz sinusoid played at a user-defined reference amplitude. MATLAB’s native audio functions (e.g., audioplayer.m) clip signal values greater than digital full scale (i.e., ±1); a calibration reference root-mean-squared amplitude of 0.4 is recommended to avoid audio clipping. The corresponding acoustic output of the sound hardware chain (i.e., MATLAB^®^ → sound card → headphones) is then measured using a sound pressure level (SPL) meter and spectrum analyzer to ensure that distortion is within acceptable limits (i.e., low total harmonic distortion). The user can then set a variable (“CaldB”) in the systemInfo.mat file to this measured system SPL. “CaldB” represents the maximum obtainable output of PsyAcoustX (and full signal path) before distortion.

### Overview of the GUI Interface and Program Layout

**FIGURE 1:**
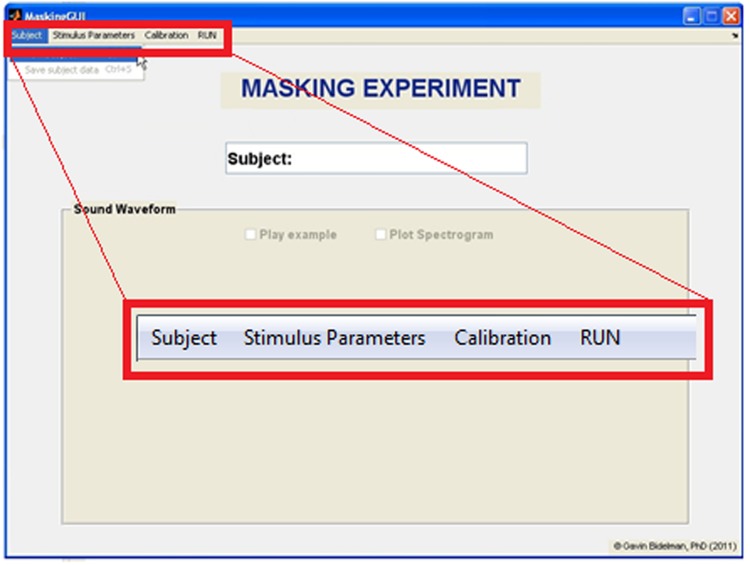
***Home window* of the PsyAcoustX GUI for psychoacoustics research.** From the home screen, users can enroll new subjects to their experiment, define stimulus parameters, calibrate their system, and run the selected experiment.

PsyAcoustX provides a fully functional, point-and-click GUI interface. This makes it possible for the end user to conduct a multitude of psychoacoustics paradigms without having to script or program (although user-based routines are easy to incorporate in the MATLAB^®^ programming language). A screenshot of PsyAcoustX’s *home window* for the masking module is shown in **Figure 1**. Basic operations including subject enrollment, experiment creation, setting stimulus parameters, calibration, and run functions (detailed below) are available directly from the program’s home window.

**FIGURE 2:**
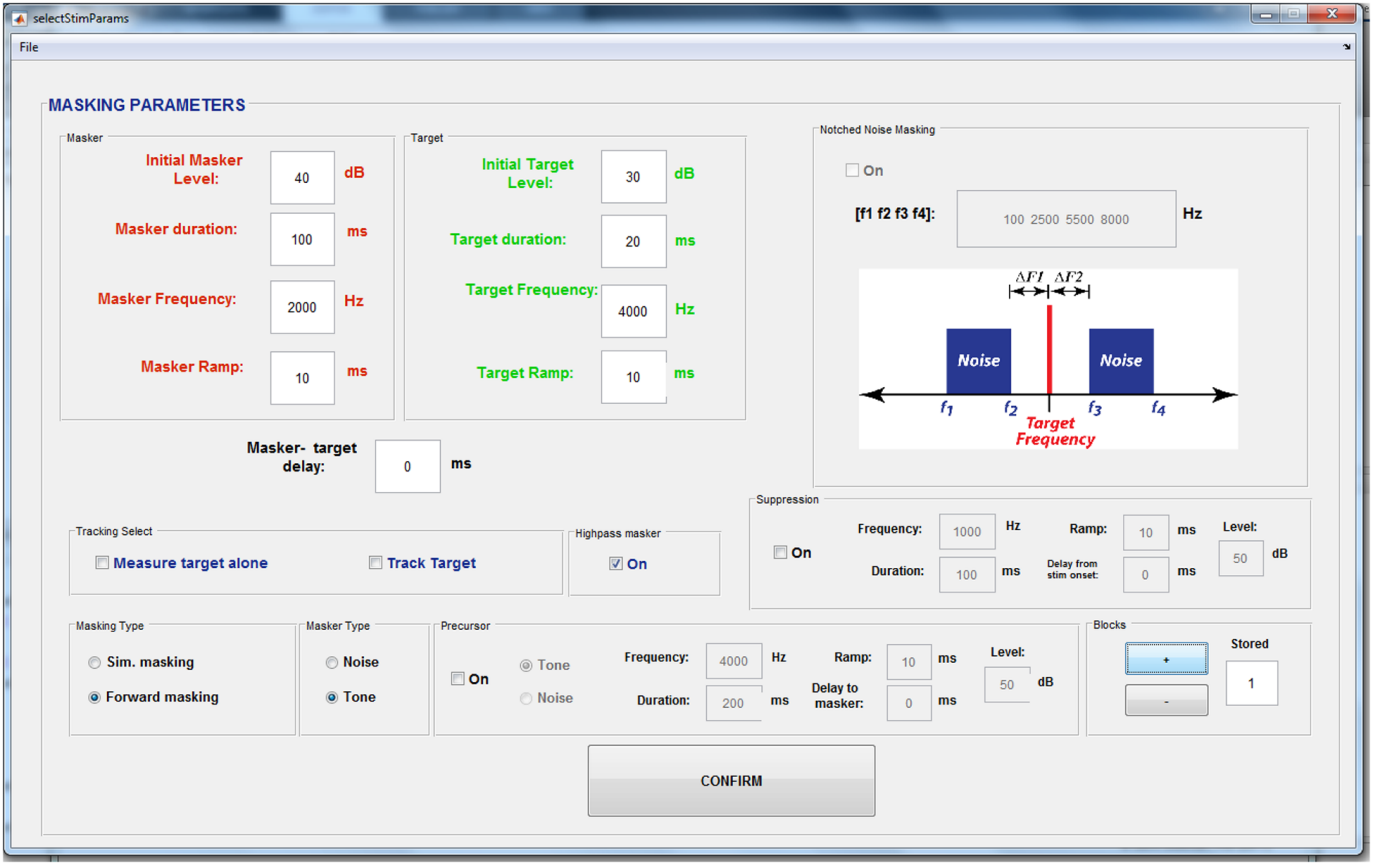
**The PsyAcoustX *stimulus generation window*.** A large number of tunable stimulus parameters are accessible to the user including properties of the probe, maskers, and control sounds as well as their spectrotemporal relations to one another (e.g., masker-probe delay). Precursor signals (Jennings et al., 2009; Roverud and Strickland, 2014), suppression (Duifhuis, 1980), and notched noise (Patterson, 1976; Glasberg and Moore, 1990; Jennings and Strickland, 2012) options are also available for more advanced masking paradigms. A toggle to implement high-frequency masking to limit off-frequency listening (Patterson and Nimmo-Smith, 1980; O’Loughlin and Moore, 1981; Jennings and Strickland, 2012) is also provided.

Users can build auditory stimuli based on the needs of their paradigm, using the *Stimulus generation window* (**Figure 2**). PsyAcoustX was originally designed for auditory masking experiments and we have included a large number of tunable stimulus parameters for users to adjust properties of the probe, maskers, and control sounds as well as their temporal and spectral relations to one another (e.g., masker-probe delay). Precursor signals (Bacon and Healy, 2000; Jennings et al., 2009; Roverud and Strickland, 2014), suppression (Duifhuis, 1980), and notched noise (Patterson, 1976; Glasberg and Moore, 1990; Jennings and Strickland, 2012) options are also available for more complex masking paradigms. Additionally, we have included a toggle to implement a secondary high-frequency masker to limit off-frequency listening (Patterson and Nimmo-Smith, 1980; O’Loughlin and Moore, 1981; Jennings and Strickland, 2012). In our experience, these options promote maximum flexibility and make it possible to run a myriad of masking-based protocols.

**FIGURE 3:**
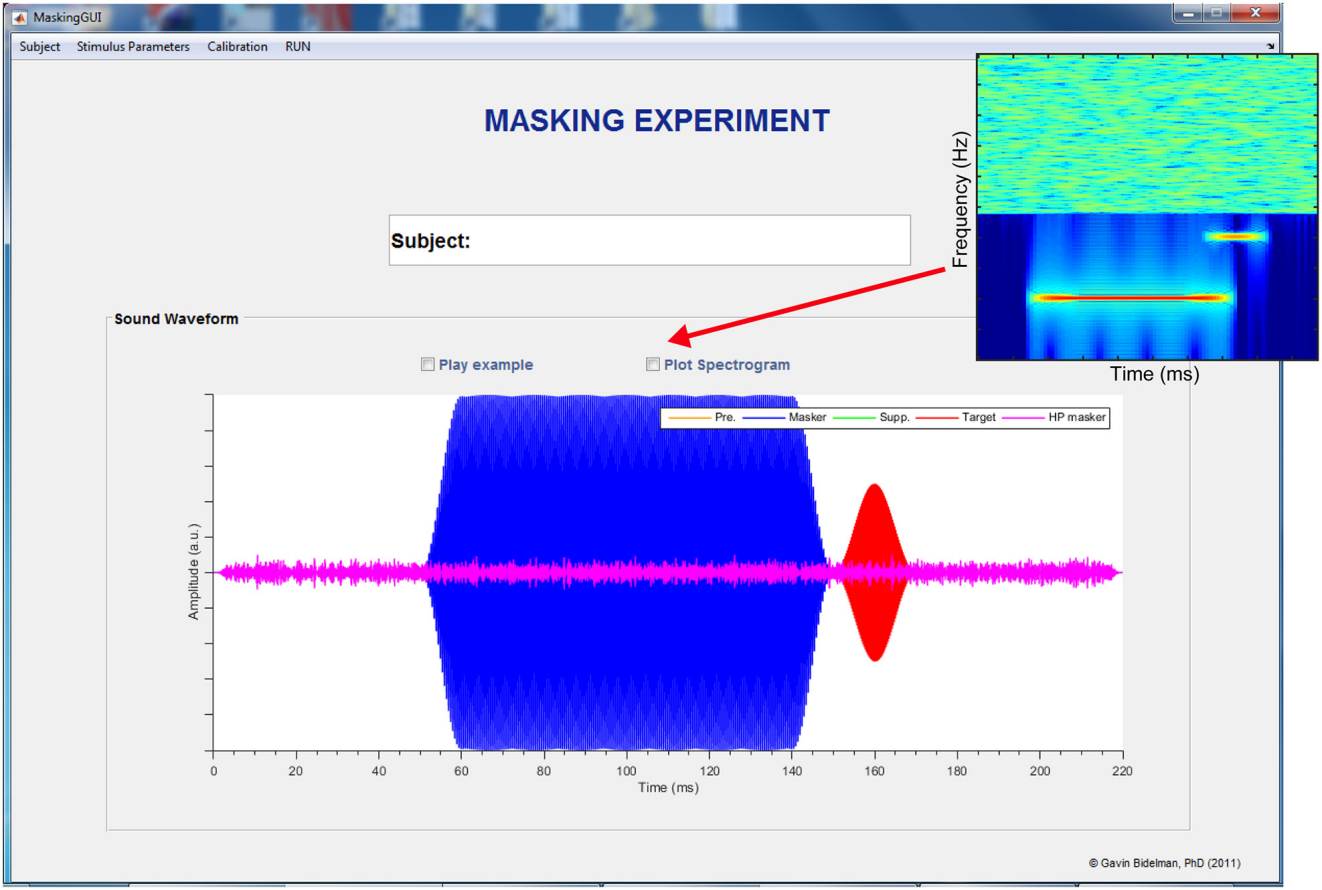
**The PsyAcoustX *stimulus visualization window*.** Once desired stimulus parameters are selected the program provides a convenient graphical representation to confirm the time course and spectral (inset) details of the stimulus. Users can also play an example of the stimuli to familiarize themselves with the listening task. The example here illustrates a forward masking condition (off-frequency masker). Low-level high-pass masking noise is also implemented to limit off-frequency listening (Patterson and Nimmo-Smith, 1980; Jennings and Strickland, 2012).

Multiple stimulus conditions can be loaded into a single experiment, allowing the researcher to define all experimental conditions, run these conditions in a random order, and repeat conditions as necessary (see “Enroll Subject Feature”). Once the desired parameters are selected and condition blocks loaded, the program plots a spectrogram in PsyAcoustX’s *home window* to allow the user to verify the time course and spectral details of the stimuli (**Figure 3**). PsyAcoustX also allows the participant to play an example of the stimulus prior to starting the experiment to familiarize him/herself with the task.

**FIGURE 4:**
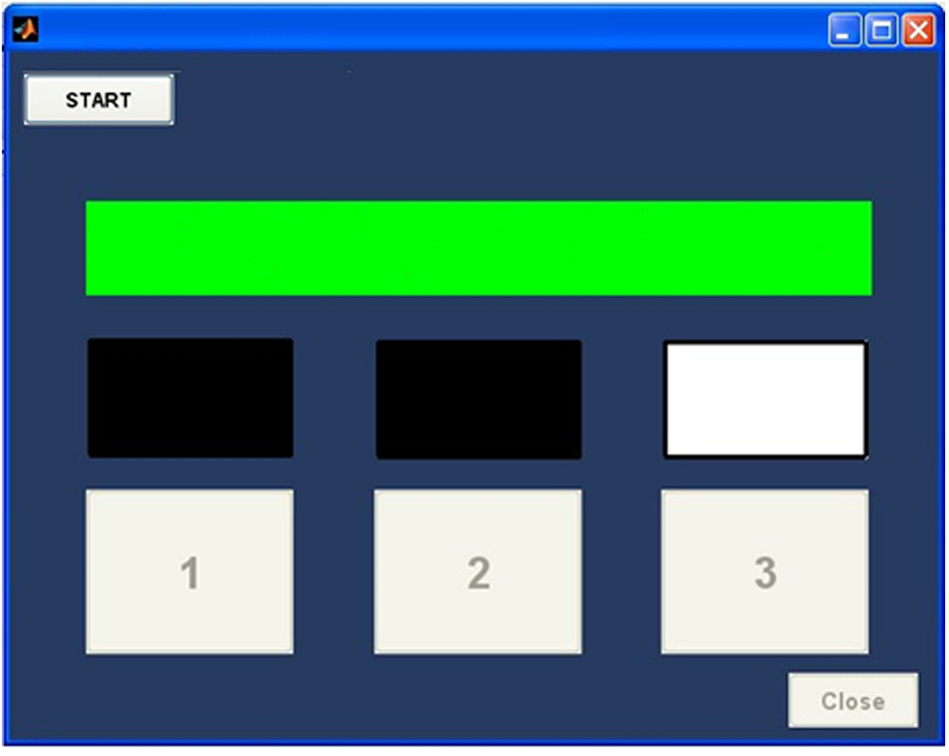
**The PsyAcoustX *response window*.** The program implements a 3IFC paradigm (2-down, 1-up tracking; Levitt, 1971) via a simple three-button response interface. Users initiate the experimental run through a START button. Behavioral thresholds are then measured adaptively. The response box includes lights to visualize the presentation order of the intervals and another graphical light for response feedback (green = correct; red = incorrect).

PsyAcoustX uses a common experimental engine for all of its experimental modules. Experiments are implemented as a three-interval forced choice (3IFC) design with a 2-down—1-up adaptive tracking rule (Levitt, 1971). That is, one interval contains the probe while the other two do not (i.e., “noise” intervals). PsyAcoustX’s *response window* is shown in **Figure 4**. The response box includes lights to visualize the presentation order of the 3IFC task and another graphical light for feedback (green = correct; red = incorrect). The 3IFC paradigm provides a simple three button response interface for subjects. The common tracking rule provides a consistent criterion performance level (i.e., 71%; Levitt, 1971) for all experimental data. Thresholds for a given task are measured adaptively. Tracking on the probe level or masker level is possible within the masking module of PsyAcoustX. In other words, this allows the researcher to fix either the probe or masker while measuring threshold. One could, for example, measure either a masking pattern (Egan and Hake, 1950) or a psychophysical tuning curve (PTC; Moore, 1978) depending on whether threshold is measured by varying the probe, or masker, respectively.

During data collection, a small window (*run tracker* panel) displays the stimulus parameters, the subjects’ response history, the level of the dependent variable (e.g., probe level), and the threshold and standard deviation of the previous run. If two monitors are available on the PC system, the end user can position this window so it is not visible to the subject. This window is useful when monitoring the subject’s progress within a run, and when keeping a paper record of experimental data.

### Data Management

Participants’ data are stored in “test” files and “completed” files, in the TXT format. Test files specify the stimulus parameter used while acquiring a given threshold measurement. Completed files contain a copy of the stimulus parameters, the subject’s responses for each individual trial in the adaptive track, and the measured threshold and standard deviation. Data management occurs automatically when subjects are enrolled in an experiment using the *enroll subject window*. This feature enables automatic test file and completed file generation. This feature also stores the information of test/completed files in Excel^®^ and MATLAB^®^ formats, to facilitate GUI automation and data export. Test files can also be saved and loaded manually using menu items in the *stimulus generation window* of PsyAcoustX.

### Enroll Subject Feature

**FIGURE 5:**
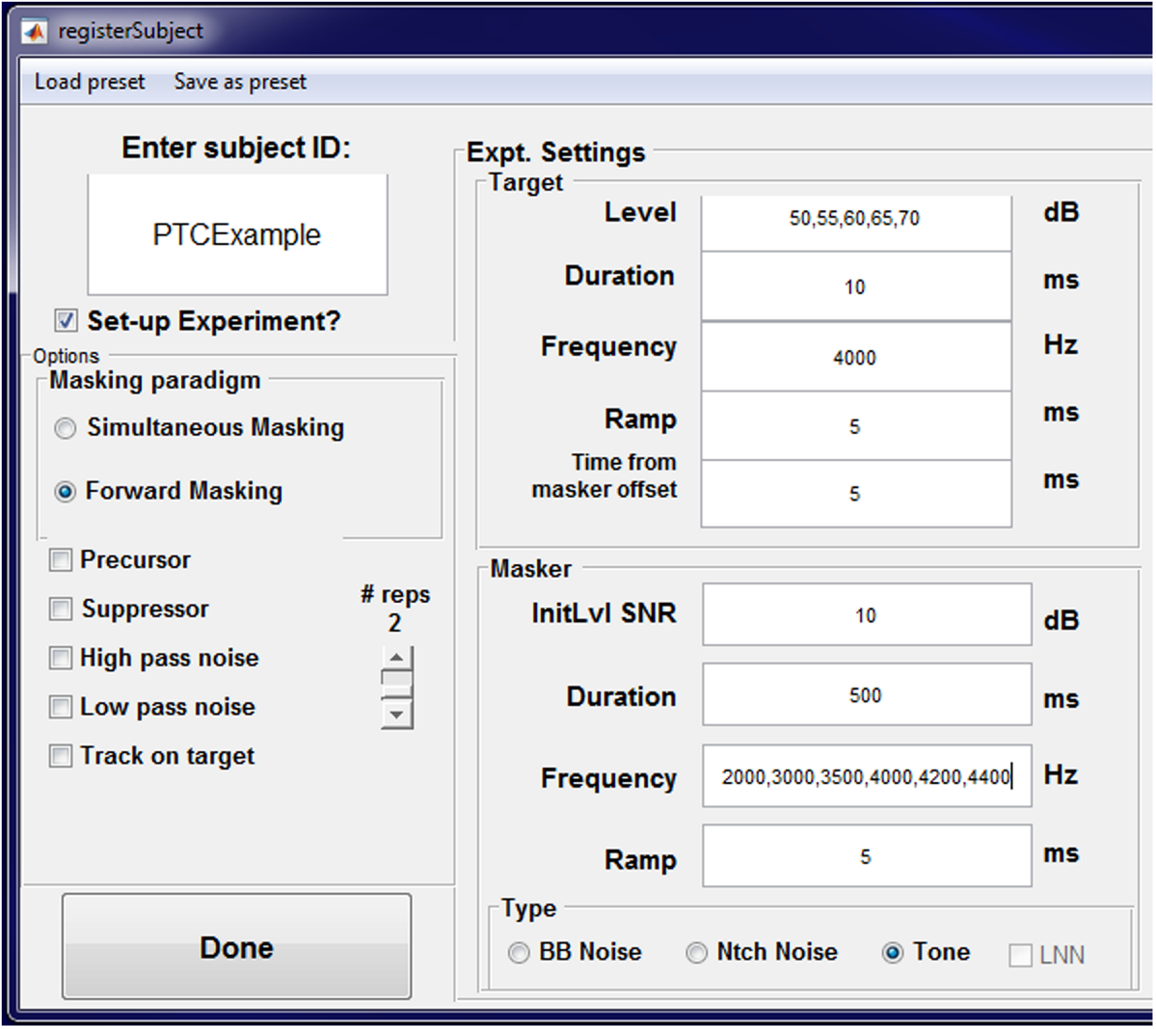
**The PsyAcoustX *enroll subject window*.** This window appears when the user selects “New Subject” from PsyAcoustX’s *home window*. Fields in the *enroll subject window* allow for multiple entries, separated by commas. When multiple entries are provided, PsyAcoustX will generate all possible stimulus conditions associated with the entries in the fields, and make a folder to store these conditions for the subject designated in “subject ID.” The example provided demonstrates a hypothetical experiment measuring psychophysical tuning curves with a 4000 Hz probe at several probe levels.

One of the more convenient features of PsyAcoustX is the ability to “enroll” a subject and store various experimental conditions. This feature allows the user to enter all possible values assumed by a given stimulus parameter during an experiment. When enrolling a subject (done using the menu bar in PsyAcoustX’s *home window*), the *enroll subject window* appears (**Figure 5**), which contains editable fields for all possible stimulus parameters. Most of these fields will accept single or multiple entries. For example, consider an experiment that involves measuring PTCs for several probe levels using a 4000-Hz probe. In the probe level and masker frequency fields, the user can enter all probe levels (e.g., 50, 55, 60, 65, and 70 dB SPL), and masker frequencies (e.g., 2000, 3000, 3500, 4000, 4200, and 4400 Hz), as well as other settings (e.g., probe frequency = 4000 Hz). Once the user submits the desired stimulus parameters, PsyAcoustX confirms the user-selected conditions in a table. After confirming the settings, PsyAcoustX then creates an array of folders and files associated with the subject, the experiment, and the conditions to be measured in the experiment. Moreover, these files and folders are automatically updated to track the participant’s progress during data collection.

If multiple subjects are to be run on the same experiment, the end user can save the experiment’s stimulus parameters as a “preset,” and load this preset when enrolling additional subjects. When experimental conditions are created using the *enroll subject window*, the user has three options for subsequent runtime. First, the user can select to continue with data collection, which will result in PsyAcoustX loading the next experimental condition and prompting the subject to start the run. Second, the user can select to start with a “warm up” condition. In this case, PsyAcoustX will load the next experimental condition, but will not save the data, nor flag the condition as completed after the subject’s thresholds are collected. This warm-up feature is useful to acclimatize subjects at the beginning of a testing session. Finally, the user can select to run a single user-defined condition. In this case, PsyAcoustX allows the user to navigate to a manually generated test file to run a single condition.

## Results and Discussion

We now demonstrate some of the functional capabilities of the PsyAcoustX program. Various psychoacoustic paradigms are introduced and representative data are presented to illustrate the program’s flexibility.

### Audiometric Hearing Thresholds

**FIGURE 6:**
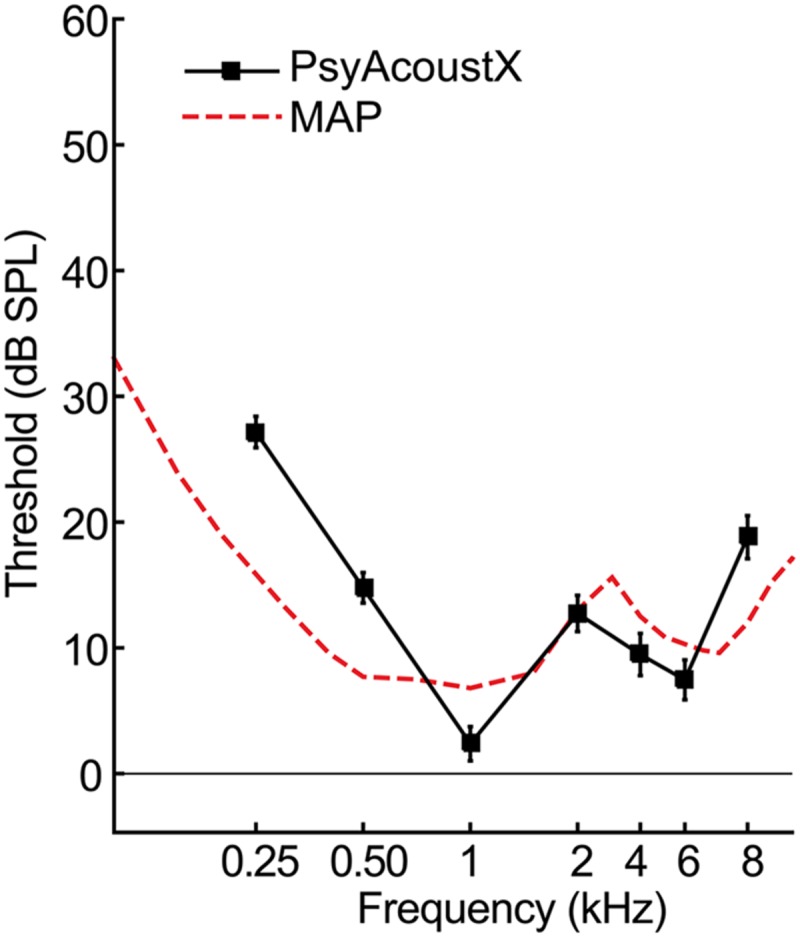
**Representative audiometric hearing threshold data.** Average air conduction hearing thresholds (i.e., audiograms) for *n* = 17 normal hearing listeners. Also shown for comparison is the minimum audible pressure (MAP, dashed line; Killion, 1978), representing auditory thresholds measured under headphone listening. Audiometric thresholds measured via PsyAcoustX agree well with those measured via other hardware/software platforms. Errorbars = ±1SEM.

Auditory experiments typically involve first measuring listeners’ thresholds for long tones in quiet at octave frequencies from 250 to 8000 Hz, as would be done clinically, to rule out confounds of hearing acuity in psychoacoustic tasks. In PsyAcoustX, it is straightforward to measure these “audiometric” thresholds by turning off all maskers and tracking the probe frequency of interest. Detection thresholds are then measured adaptively based on PsyAcoustX’s stock 3IFC paradigm with a two-down one-up tracking rule, which converges on 71% correct performance (Levitt, 1971). **Figure 6** shows the average audiometric thresholds for *n* = 17 normal hearing listeners. Additionally, the minimum audible pressure (MAP; Killion, 1978) is shown, representing auditory thresholds measured under headphone listening. As is illustrated in the figure, audiometric thresholds measured through PsyAcoustX agree well with typical hearing thresholds measured via other hardware. That is, once calibrated, PsyAcoustX can provide a tool for threshold measurements that is similar to audiological-grade systems. Subtle discrepancies in thresholds measured with PsyAcoustX and MAP curves are due to differences in the calibration reference between measures. MAP curves represent SPLs in the ear canal at each individual measurement frequency. Conversely, thresholds measured with PsyAcoustX are based on a calibration reference at 1 kHz and do not account for the frequency response of the headphones, or ear canal acoustics. We should stress that PsyAcoustX is intended for experimental purposes and not for diagnostic testing; proper audiological measurements typically require a sound booth, a quiet testing environment, calibrated earphones, and normative data.

### Simultaneous Masking

PsyAcoustX can be used to measure thresholds in simultaneous masking. Here, we present simultaneous masking data on a frequently studied effect called “overshoot” or the “temporal effect” (Zwicker, 1965). Thresholds for a short sinusoidal probe improve as the probe’s temporal position approaches the temporal center of a gated, broadband masker (e.g., Bacon, 1990; Strickland, 2001). The magnitude of this improvement (i.e., overshoot) is the difference between thresholds measured at the onset of the masker, versus the temporal center. PsyAcoustX was used to measure overshoot for a 4000-Hz probe at several probe levels (50, 60, 70, 80, and 90 dB SPL). The probe was delayed by 2-ms (short delay) or 198-ms (long delay) from the onset of a noise masker (filtered Gaussian noise, 100–8000 Hz). Masker spectrum level at threshold was the dependent variable.

**FIGURE 7:**
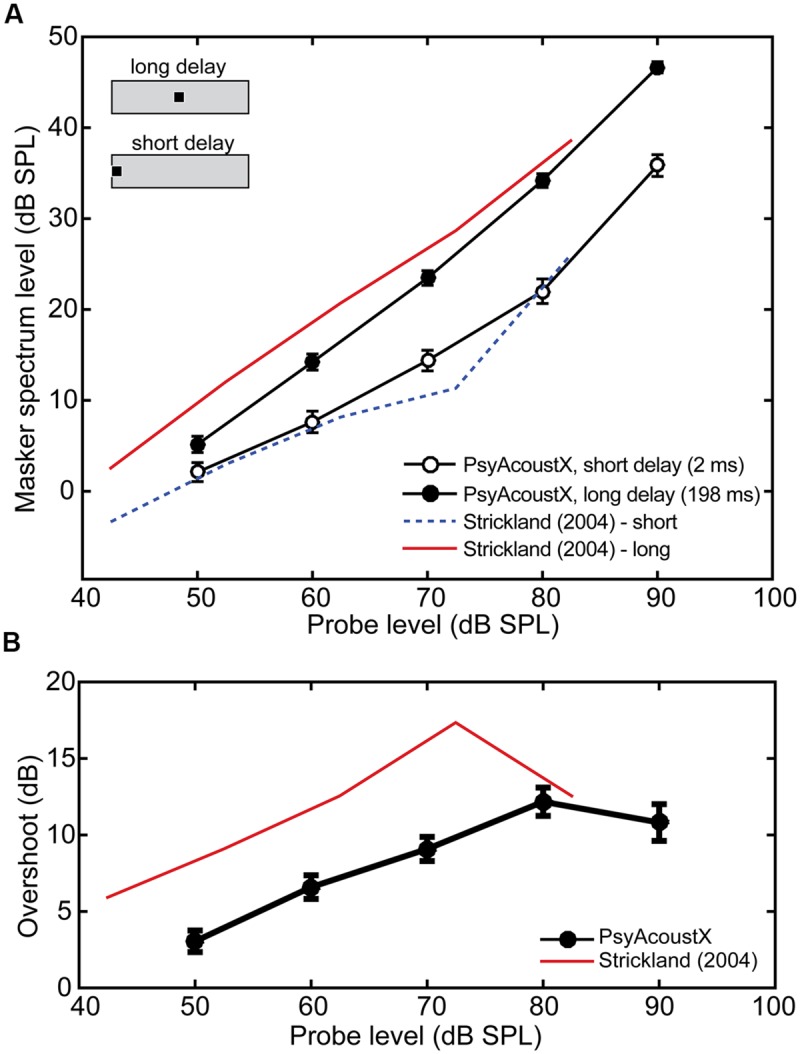
**Representative simultaneous masking (overshoot) data. (A)** Masking thresholds for probes presented at the onset (open symbols, “short delay”) or temporal center (filled symbols, “long delay”) of the masker. **(B)** Overshoot calculated from data in **(A)**. Data from Strickland (2004) are shown for comparison; their input levels have been adjusted by 7.54 dB to account for stimulus ramping.

**Figure 7A** shows the masker spectrum level at threshold as a function of probe level for *n* = 17 normal-hearing ears for the short and long delay conditions. Overshoot from these measurements is shown in **Figure 7B**, computed as the difference in detection threshold between the long and short delay (i.e., long–short). Consistent with previous studies, listeners are better at detecting the probe (i.e., they experience less masking) in the long relative to the short delay condition. Participants show ∼4–12 dB release from masking when the probe is temporally centered in the masker compared to when it occurs concurrent with its onset (**Figure 7B**). Data from Strickland (2004) are also included for comparison.

### Temporal Masking: Forward and Backward Masking

Temporal (i.e., non-simultaneous) masking is commonly used as an assay of temporal resolution. Temporal masking can also provide a psychoacoustic estimate of the basilar membrane’s compressive non-linearity (Oxenham and Plack, 1997; Nelson et al., 2001), and cochlear frequency selectivity (Moore, 1978; Shera et al., 2002). In forward masking, the interfering masker (e.g., tone or noise) precedes the probe; whereas in backwards masking, the masker follows the probe. Forward masking is thought to reflect direct physiological mechanisms of cochlear processing, e.g., the recovery from short-term adaptation or “ringing” of the basilar membrane (Harris and Dallos, 1979; Moore, 2003). Physiological mechanisms of backward masking are not well understood but are thought to result from “top–down” mechanisms such as selective attention and/or listeners’ confusion between the probe and masker (Wright, 1998; Moore, 2003).

**FIGURE 8:**
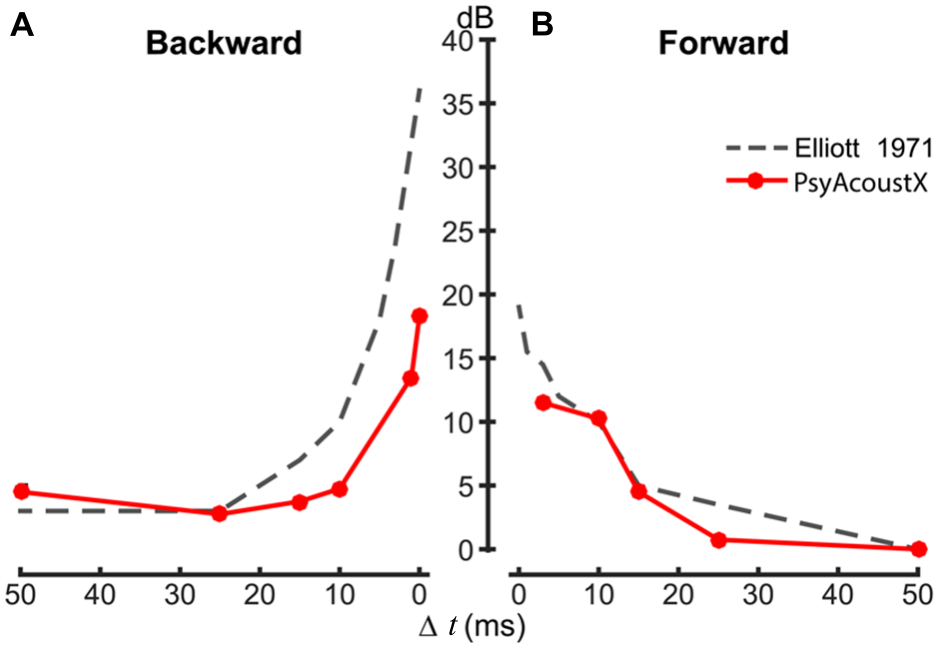
**Representative temporal (forward and backward) masking data. (A)** Backward and **(B)** forward masking functions. The time delay between the noise masker and probe tone (Δ*t*) was varied parametrically between –50 and +50 ms; Δ*t* = 0 represents the condition where the masker and probe are contiguous. Temporal masking functions were modeled after stimulus parameters from Elliott (1971) (10 ms, 1-kHz probe masked by 70 dB SPL, 50-ms wideband noise). Data measured in PsyAcoustX agree well with experimental data and show the characteristic asymmetry in temporal masking; forward masking is more effective (i.e., persists longer) than backwards masking.

Both forward and backward masking paradigms are available in PsyAcoustX. Temporal masking functions were measured according to the parameters described by Elliott (1971) (10-ms, 1-kHz probe masked by 70-dB, 50-ms wideband noise). In this example, we varied Δ*t*, the time delay between the masker and probe. Representative temporal masking data from a normal hearing listener are shown in **Figure 8**. Temporal masking functions show the threshold elevation (in dB) for the probe when it precedes (**Figure 8A**, backwards) and follows (**Figure 8B**, forward) the masker. Consistent with data in the literature (Elliott, 1971), **Figure 8** shows the consistent asymmetry observed in temporal masking studies between forward and backward conditions. Masking is more effective and persists for longer masker-probe intervals (Δ*t*) in the forward compared to the backward case. The improved backward masking thresholds in our data relative to those of Elliott (1971) are likely attributable to the extensive musical training of our listener (first author), which is known to reduce the effects of backward masking (Strait et al., 2010).

### Psychophysical Tuning Curves

The peripheral auditory system (i.e., cochlea) is typically conceived of as bank of overlapping bandpass filters that performs a spectral decomposition on the incoming sound (Fletcher, 1940; Patterson and Moore, 1986). In humans, the magnitude response of the auditory filters can be estimated using a number of psychophysical techniques. In the simplest simultaneous masking approach (Moore, 1978), the detection of a low-level probe is measured in the presence of a masking tone. The probe encourages the participant to “listen” at a specific cochlear location, or characteristic frequency (CF), while the masker interferes with the detection of the probe (Moore, 1978). Detectability of the probe varies dependent on the spectral proximity to the masker. The tuning (i.e., frequency selectivity) of a given cochlear location (i.e., CF) can then be estimated by plotting the masked probe threshold as a function of masker frequency to derive the so-called PTC. PTCs can be measured using simultaneous and forward masking approaches (for comparisons, see Moore, 1978; Bidelman et al., 2014) and/or using tonal or noise maskers. Although details are beyond the scope of the present report, each of these approaches has various strengths/weakness and controls for other extraneous factors, e.g., cochlear suppression and/or beating cues (Abbas and Sachs, 1976; Moore, 1978; Oxenham and Shera, 2003).

Relevant to the present work, we have successfully used PsyAcoustX in our recent studies to estimate cochlear tuning in human listeners via PTCs (Bidelman and Syed Khaja, 2014; Bidelman et al., 2014). Here, we present PTCs from a representative normal hearing listener (first author), measured at probe frequencies of 500 and 2000 Hz. PTCs were measured using forward masking with a 300-ms pure tone masker followed immediately by a 35-ms probe tone (0 ms masker-probe delay; for details, see Bidelman et al., 2014). We used 10 masker frequencies (i.e., five below and above the probe frequency). Masked threshold as a function of masker frequency provides an estimate of the PTC function, that is, the listeners’ auditory filter shape at a given CF.

**FIGURE 9:**
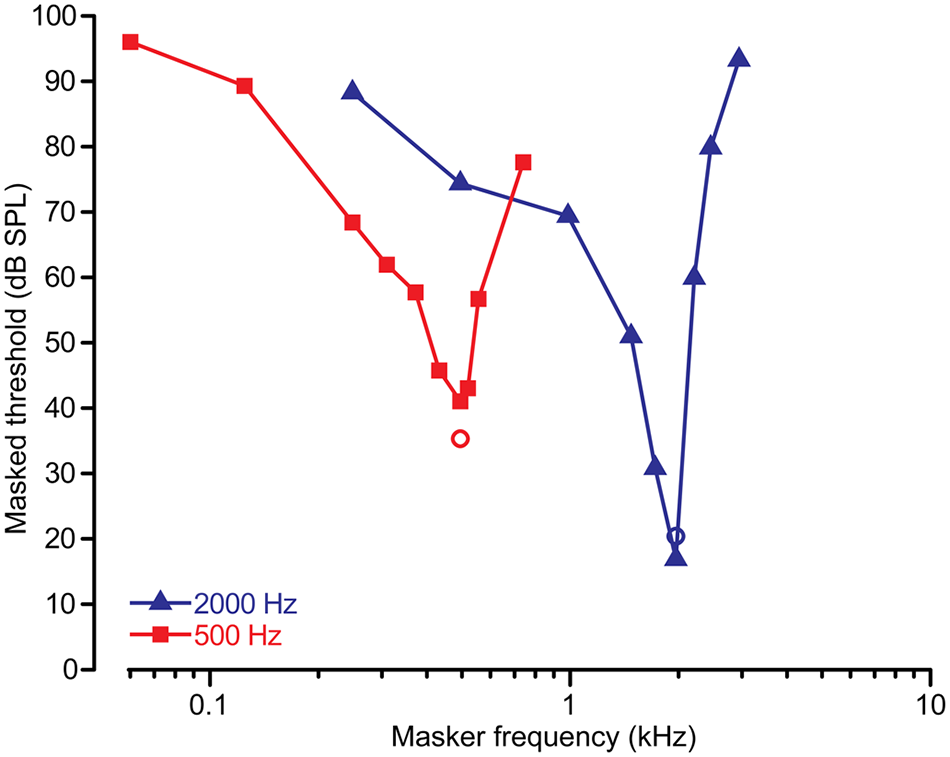
**Representative psychophysical tuning curve (PTC) data.** PTCs were measured for signal frequencies of 500 and 2000 Hz using a forward masking paradigm (Bidelman et al., 2014). Circles show the level and frequency of the probe. PTCs show the typical “V-shape” with a low-frequency tail, highly selective tip, and steep high-frequency skirt characteristic of auditory filters. Also apparent is the higher quality factor (i.e., sharpness) in human tuning for higher compared to lower characteristic frequencies (CFs) (Shera et al., 2002).

Forward masked PTCs are shown in **Figure 9**. PTCs show the typical “V-shape” with a low-frequency tail, highly selective tip, and steep high-frequency skirt characteristic of auditory filters measured via psychophysical paradigms (Moore, 1978; Sek et al., 2005; Jennings and Strickland, 2012; Bidelman et al., 2014). It should be noted that other approaches to estimating auditory filters are also possible in PsyAcoustX. The notched noise method is another common technique to measure auditory filter bandwidths via masking. In this approach, the detectability of a probe is measured amidst noise with a notch of varying spectral width to derive the shape of the auditory filters (Patterson, 1976; Glasberg and Moore, 1990; Jennings and Strickland, 2012). In PsyAcoustX, the generation of both symmetric and asymmetric notched noise are possible, allowing the user to measure the inherent asymmetries in auditory filter profiles (Unoki et al., 2006).

### Gap Detection

By nature, hearing involves decoding and interpreting changes in acoustic stimuli over time. The ability of the auditory system to follow these changes is known as “temporal resolution.” One measure of temporal resolution involves detecting a temporal gap in an otherwise steady-state stimulus, often called a “marker.” The minimum audible gap duration is known as the gap detection threshold (GDT) (Fitzgibbons, 1983; Florentine and Buus, 1984; Florentine et al., 1999).

**FIGURE 10:**
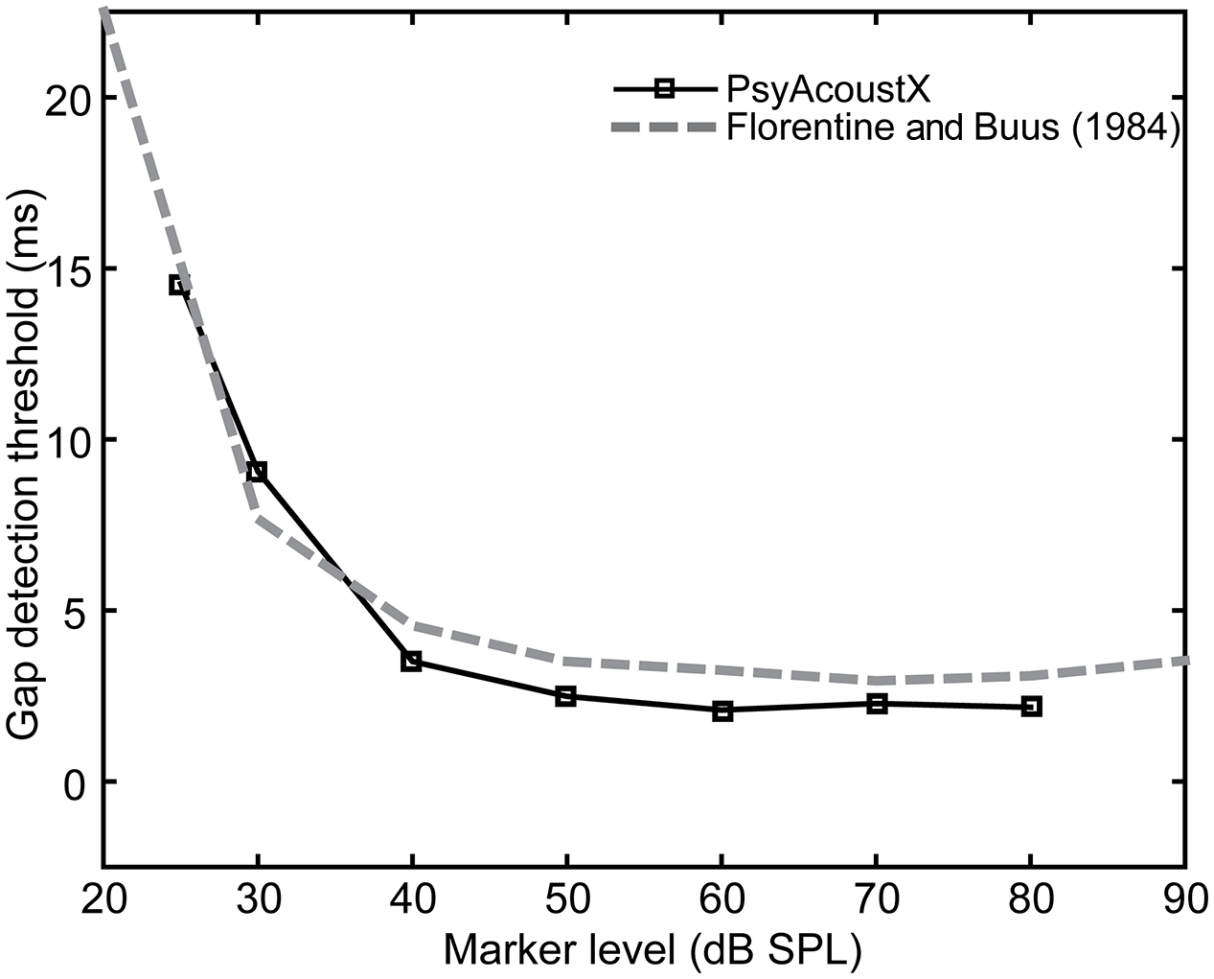
**Representative gap detection thresholds (GDTs) as a function of marker level.** Squares are data collected using PsyAcoustX in one normal hearing subject. Data from Florentine and Buus (1984) are shown for comparison.

GDTs and other temporal aspects of hearing may deteriorate with age and with cochlear hearing loss; however, some methodological factors complicate this simple interpretation (for review, see Reed et al., 2009). Regardless, gap detection remains a popular measurement of temporal resolution in hearing research. GDTs for a single listener measured using PsyAcoustX are shown in **Figure 10**. The 500-ms marker was a broadband noise (100–8000 Hz) presented at several SPLs, with the temporal gap positioned centrally within the marker. Data from Florentine and Buus (1984) are also provided for comparison. Consistent with their study, GDTs improved rapidly with increasing marker level and then remained relatively constant.

### Amplitude Modulation Detection

Gap detection thresholds provide only a single estimate of a listener’s auditory temporal resolution (Fitzgibbons, 1983; Florentine and Buus, 1984; Florentine et al., 1999). Moreover, due to the short duration stimuli used in typical GDT paradigms, stimuli may be confounded by additional cues of spectral splatter. This can improve perceptual detection and thus, can overestimate a listener’s temporal resolution thresholds (Moore, 2003).

To circumvent acoustic issues of GDT paradigms and provide a more complete, functional description of auditory temporal resolution, some psychoacousticians measure the temporal modulation transfer function (TMTF; Viemeister, 1979; Strickland and Viemeister, 1996; Dau et al., 1997). TMTFs measure the ability to follow or resolve amplitude fluctuations in an ongoing carrier. Often, a carrier signal (e.g., sinusoidal tone, *f*_c_) is modulated by another sinusoid (*f*_m_). Wideband noise carriers are often used to prevent altering the long-term power spectrum of the modulated stimulus and to prevent listeners from detecting audible sidebands resulting from the *f*_m_ (Burns and Viemeister, 1981). The modulation depth needed for a listener to just detect amplitude fluctuations is then recorded as a function of the modulation frequency. TMTFs typically resemble a low-pass filter with cutoff frequency of ∼100 Hz; listeners are more sensitive at detecting amplitude modulations at low compared to high modulation frequencies (Viemeister, 1979).

**FIGURE 11:**
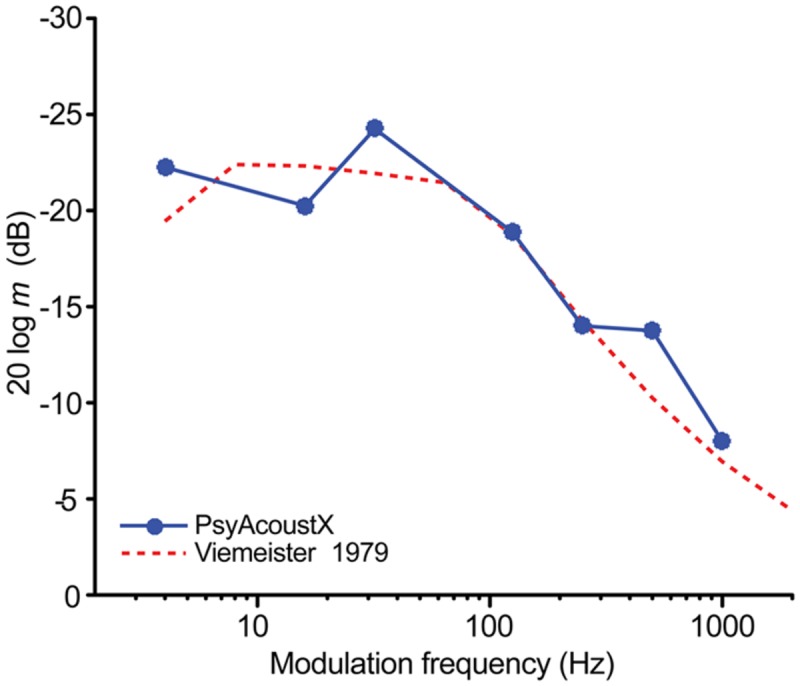
**Representative temporal modulation transfer function (TMTF) data.** The TMTF recorded from a representative normal hearing listener illustrates temporal acuity for detecting amplitude fluctuations in continuous sounds as a function of modulation frequency. Broadband noise was modulated with a sinusoidally amplitude modulated (SAM) tone of varying modulation frequencies. Data from Viemeister (1979) (their Figure 6) are shown for comparison. TMTFs reveal a characteristic low-pass filter shape indicating better temporal resolution at lower compared to higher modulation frequencies.

A representative TMTF recorded in PsyAcoustX is shown in **Figure 11**. This TMTF was measured from a normal hearing listener (first author) in response to gated 500-ms sinusoidally amplitude modulated (SAM) noise (high-pass filtered at 80 Hz) presented at 60 dB SPL. PsyAcoustX was used to track the modulation depth at threshold (in dB) for various *f*_m_s ranging from 4 to 1000 Hz (e.g., Viemeister, 1979; their Figure 6). Thresholds measured for TMTFs represent the smallest amplitude modulation that the listener can reliably detect at each modulation frequency.

## Conclusion

The current report presents PsyAcoustX, a new open-source, MATLAB^®^ -based software suite for psychoacoustics research. PsyAcoustX is advantageous compared to other platforms as it does not require dedicated hardware or programming knowledge. In addition, stimuli are generated adaptively within the program according to the participant’s response. These features make it possible for users to execute highly precise psychophysical paradigms entirely via a GUI interface and measure behavioral thresholds adaptively. Current applications available through PsyAcoustX’s engine were presented including measurement of audiometric thresholds, simultaneous and non-simultaneous masking paradigms, PTCs, temporal overshoot, gap detection, and amplitude modulation detection. While the current version of the software is geared toward psychoacoustics research and masking and temporal resolution paradigms under monaural listening, users can easily extend the base package to accommodate any number of conceivable psychoacoustic paradigms (e.g., pitch discrimination). Future iterations of the program could also extend the GUI to include binaural auditory tasks (e.g., spatial masking paradigms, and interaural time/intensity discrimination).

## Conflict of Interest Statement

The authors declare that the research was conducted in the absence of any commercial or financial relationships that could be construed as a potential conflict of interest.

## Footnotes

1 http://www.pstnet.com/eprime.cfm

2 www.neurobs.com

3 http://auditorypro.com/download/psycon/psycon.html

4 http://www.psy.unipd.it/~grassi/psychoacoustics_new.htm

5 http://psychtoolbox.org/

6 https://sites.google.com/site/psyacoustx/
