# Supplementary material for: PsyAcoustX: A flexible MATLAB® package for psychoacoustics research
Source: Front Psychol. 2015 Oct 12;6:1498. doi: 10.3389/fpsyg.2015.01498 (PMC4601020; doi:10.3389/fpsyg.2015.01498)

Contents

[Opening the GUI: 2](#_Toc339962439)

[Enrolling a new subject in an experiment: 4](#_Toc339962440)

[Measuring thresholds in quiet at multiple frequencies 5](#_Toc339962441)

[Running an experiment on an enrolled subject 6](#_Toc339962442)

[Adding a condition to an existing experiment: 12](#_Toc339962443)

[Removing conditions from an experiment: 14](#_Toc339962444)

[Calibration: 18](#_Toc339962445)

[Features: 19](#_Toc339962446)

[Playing an Example: 19](#_Toc339962447)

[runTracker: 21](#_Toc339962448)

# Opening the GUI:

To open the PSYACOUS_GUI program, first open MATLAB and then do one of the following: 1) selected the PSYCHOACOUS GUI shortcut on the MATLAB shortcuts bar (item #1 below), 2) after navigating to the PSYACOUS_GUI directory (item #2 below), type “psychoacousticsGUI” at the command line (item #3 below).


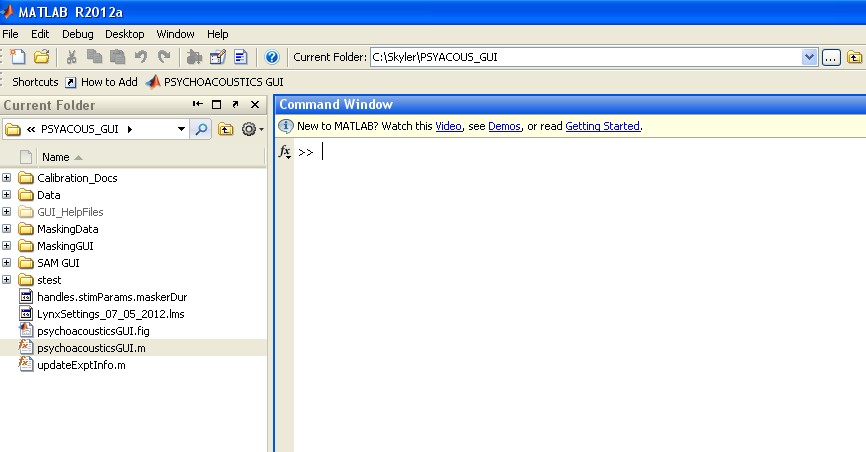


type “psychoacousticsGUI here…


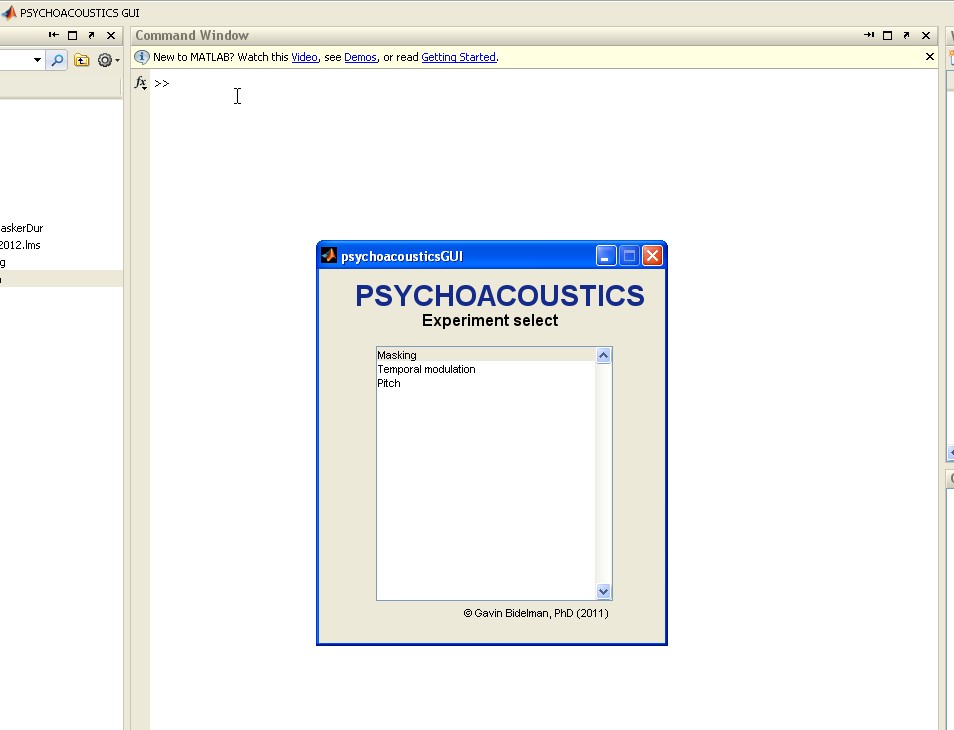
After the program has opened, a window will appear at the center of the screen title “psyacousticsGUI” and prompt the user to select a type of experiment. Currently, only the “Masking” experiment is supported, so the user should single-click “Masking” on the menu list to proceed.

After “masking” is selected from the psyacousticsGUI window, a large window titled “MaskingGUI” will open. At this point the user has several options including: 1) enrolling a new subject in an experiment involving several conditions, 2) defining the stimulus parameters for a single user-defined condition, 3) running conditions on an experiment or on a single user-defined condition, 4) adding/deleting conditions from an existing experiment or 5) calibrating the laboratory equipment. Many of these options are discussed in the sections that follow.


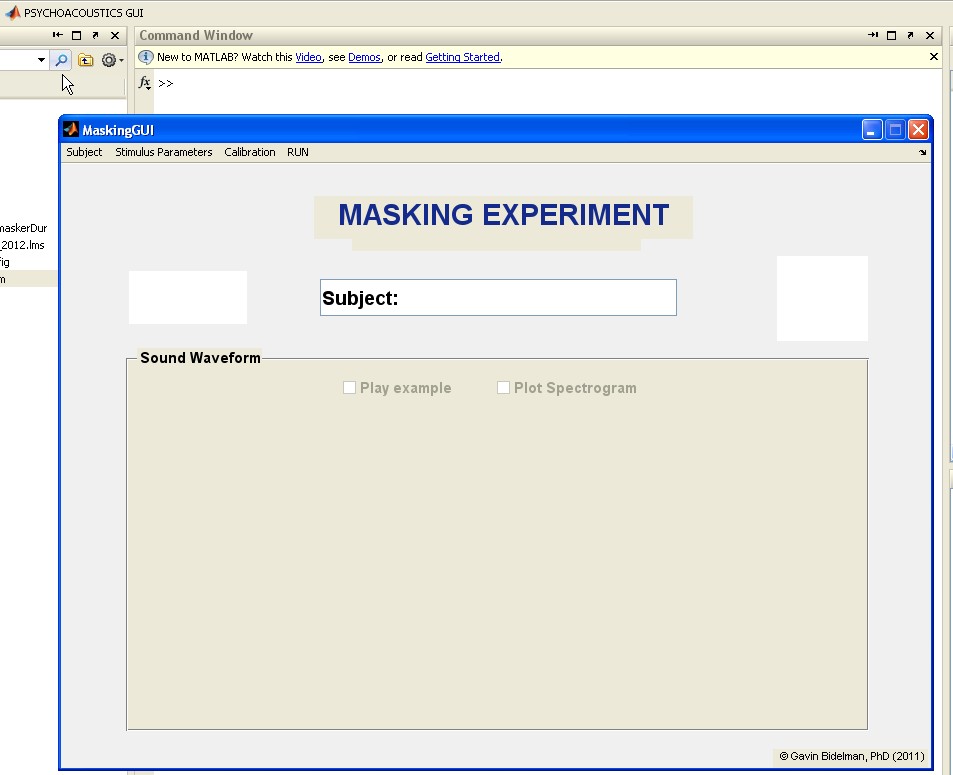


# Enrolling a new subject in an experiment:

To enroll a new subject in an experiment, select **Subject 🡪 New Subject** from the menu in the MaskingGUI window. This will open the registerSubject window.


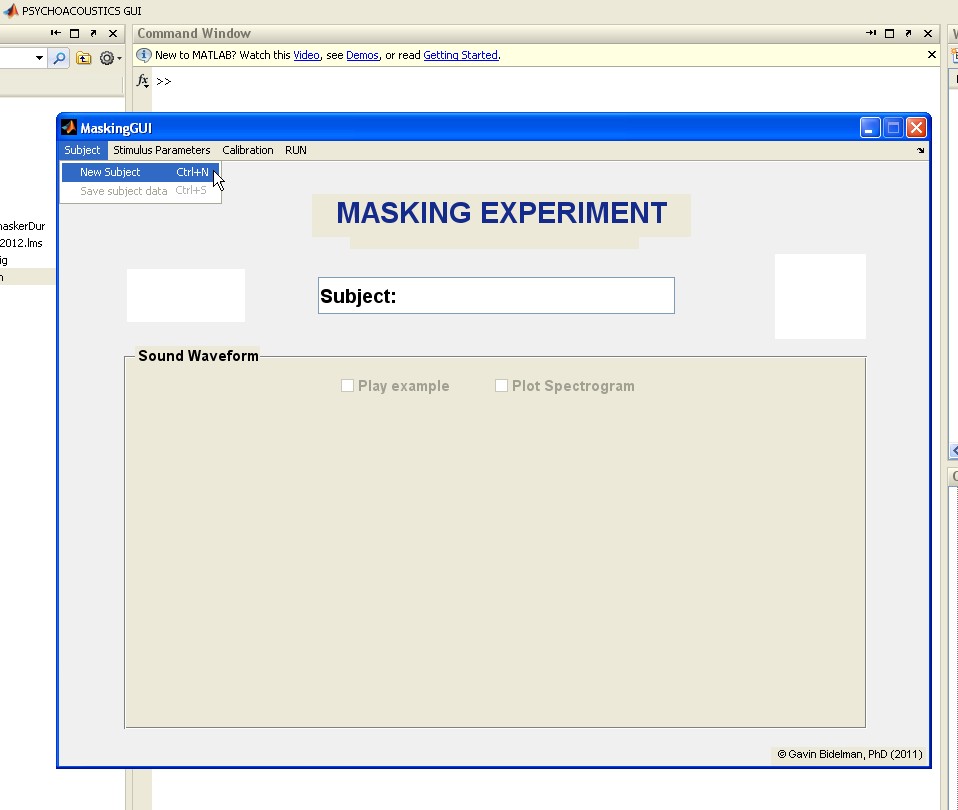


In the registerSubject window, the subject’s ID and information about the experimental stimuli is defined. The fields associated with stimulus parameters can receive several values if separated by commas. In the example below, a simultaneous masking experiment involving three signal levels and two delays from masker onset is defined.


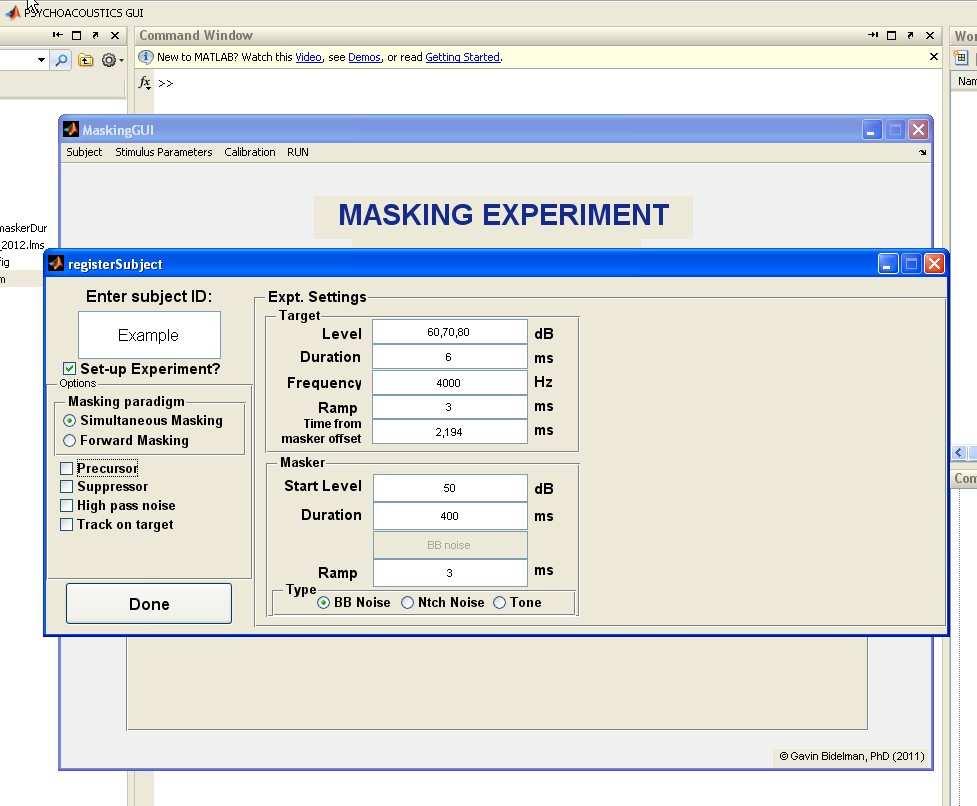


After defining the stimulus parameters in the registerSubject window, the user presses the “Done” button to proceed and a table is presented that displays all of the conditions in the experiment. This table shows the stimulus parameters as rows and condition numbers as columns. This example shows the output of the previous simultaneous masking example with three signal levels and two delays from masker onset. Notice that these signal levels and delays are combined in all possible combinations resulting in six total stimulus conditions. A given combination of signal level and delay from masker onset can be seen by observing the values for the rows labeled “delay” and “targetLevel” for a given column. At this point, the user can review the table to determine if the experiment has been set-up correctly and if so, the user then presses the OK button and then closes the window. After the window is closed, the program will return to the MaskingGUI window.


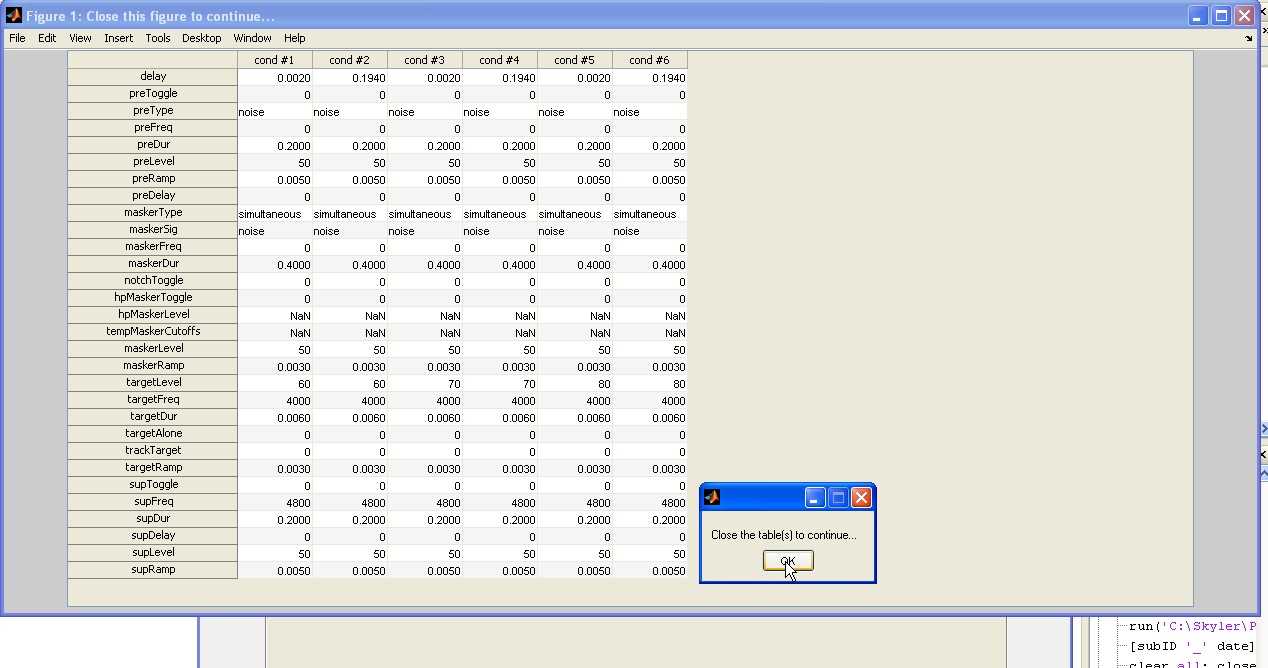


## Measuring thresholds in quiet at multiple frequencies

The Register Subject dialog can be used to create stimulus conditions similar to those used in measuring a puretone audiogram. To measure thresholds in quiet, enter the subjects ID and then set the masking paradigm to Simultaneous Masking and click the box labeled “Track on target.” In the panel labeled “target” set the Time from masker offset to zero and then select the desired duration (a scalar), ramp (a scalar) and test frequencies (a vector with each value separated by a comma). In the panel labeled “masker,” set the masker duration to be greater than the target duration (e.g. if you target is 350 ms, set your masker to 400 ms) and set the masker level to -120 dB. Finally, in the target panel, set the InitLvl SNR to result in a 20-30 dB SPL target level. For a masker that is -120 dB this initLvl SNR will be 140-150 dB. A screenshot of an experiment using audiometric frequencies and a target duration of 350 ms is provided below. The same measurement is also shown for a shorter signal duration (6 ms).


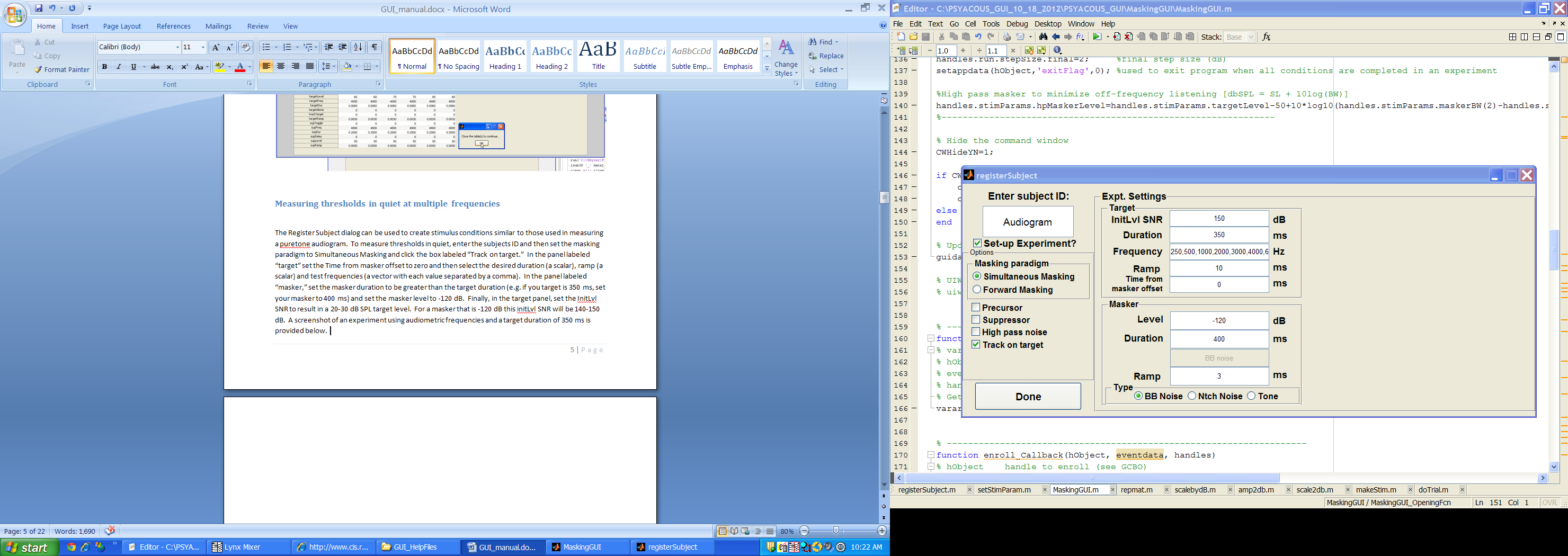


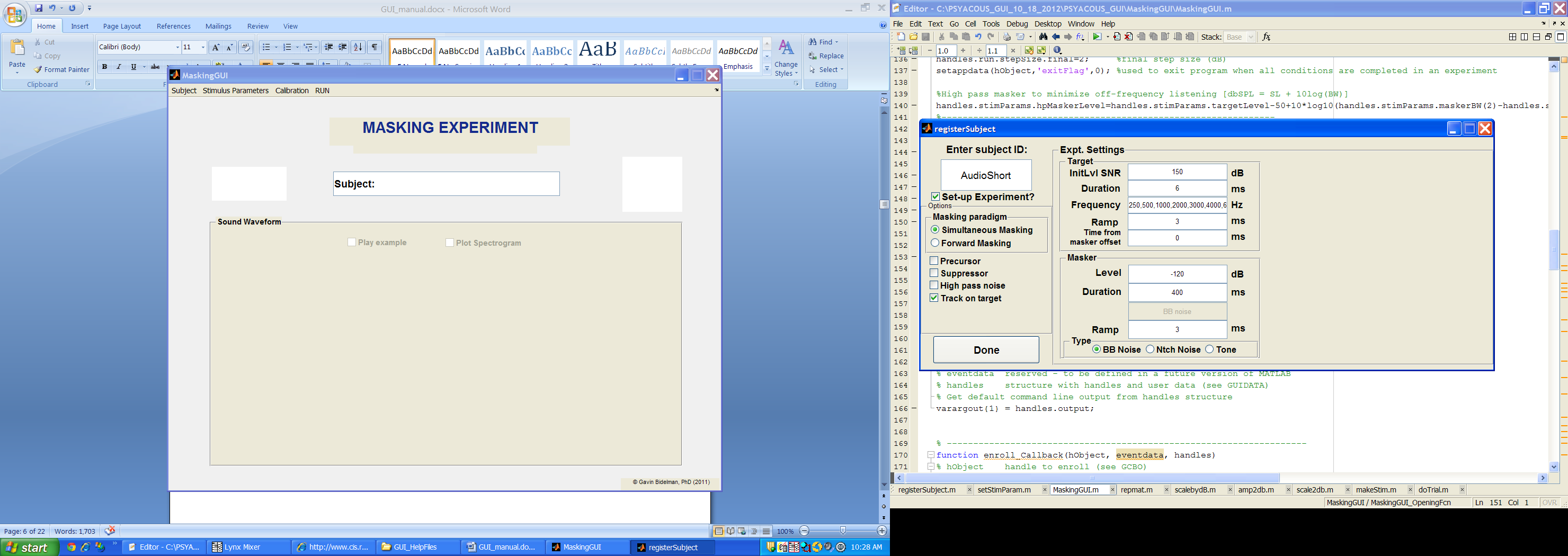


# Running an experiment on an enrolled subject

To run an experiment on an enrolled subject, select **Stimulus Parameters 🡪 Define** on the menu in the MaskingGUI window. This will open a new window titled “selectStimParams.”


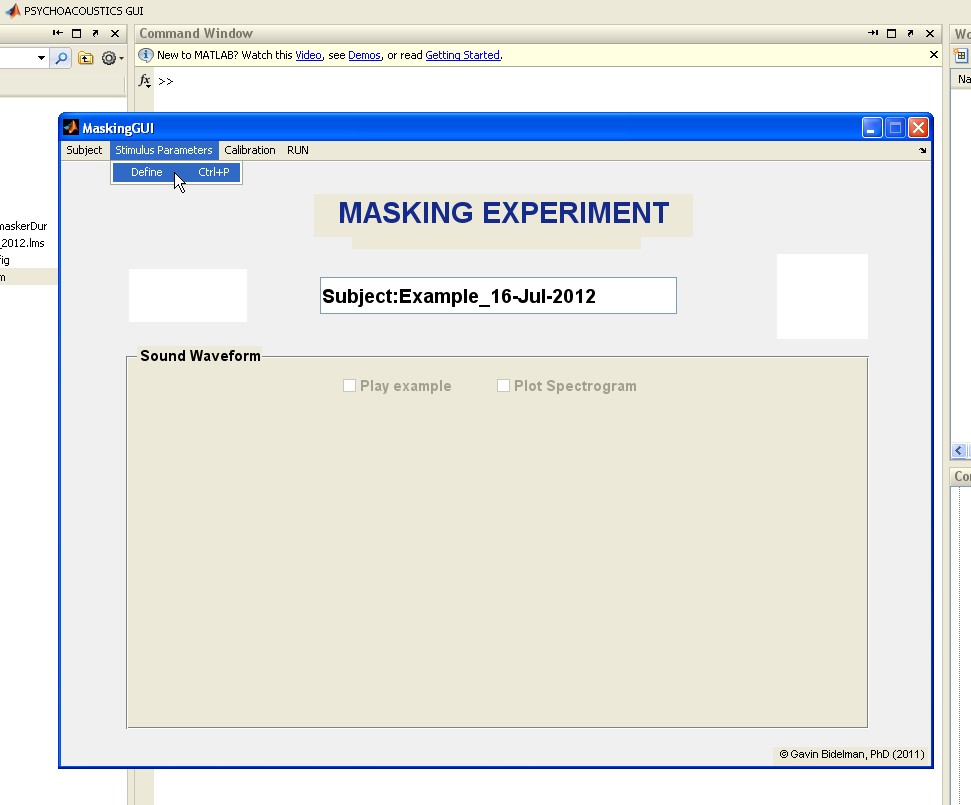


The user then selects **File 🡪 Load** from the menu in the selectStimParams window and is prompted to select one of the following options: 1) Run next condition in selected experiment, or 2) Run a single user-defined condition.


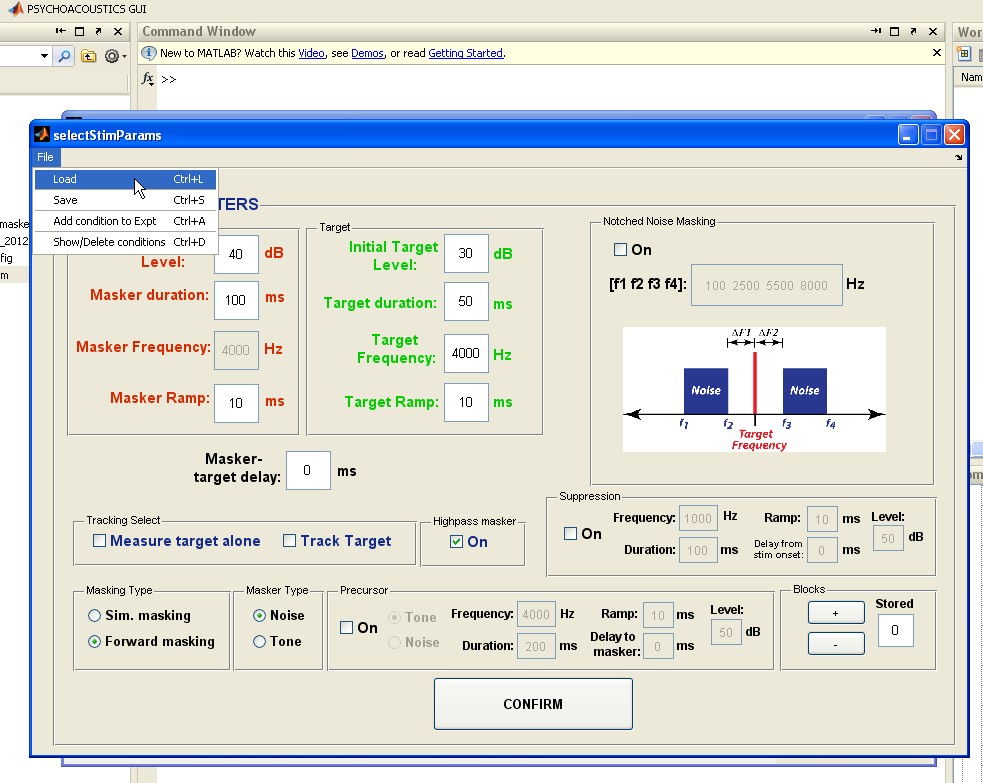


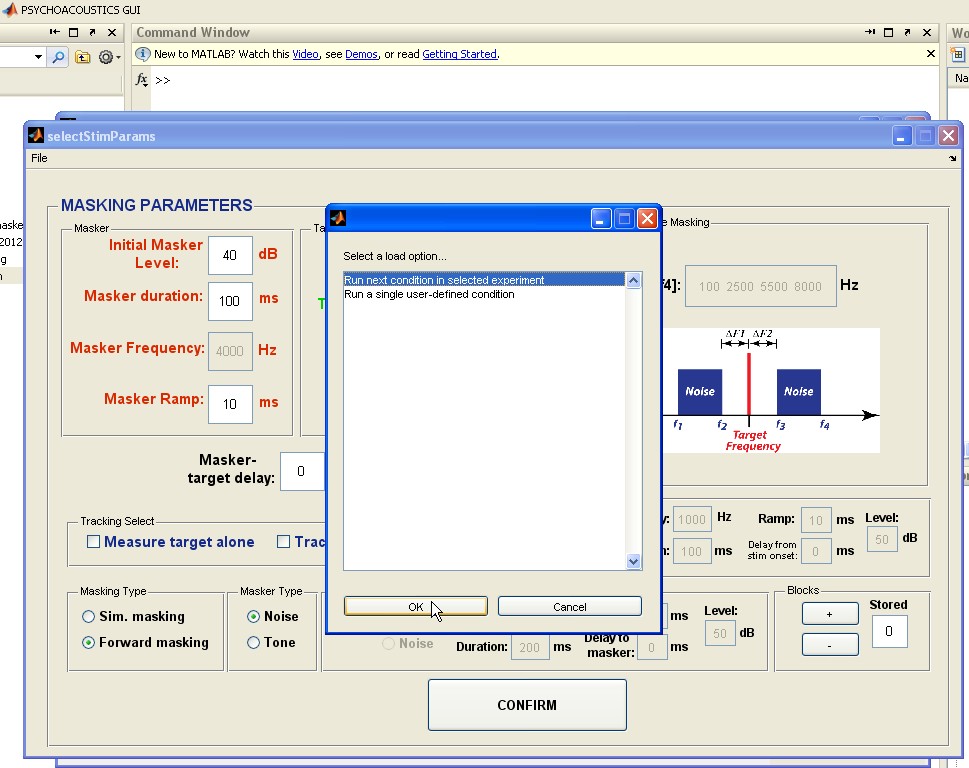
The user then selects option #1 and is prompted to locate the subjects folder and “ExptInfo.mat” file that was created when the experiment was define using the registerSubject window.


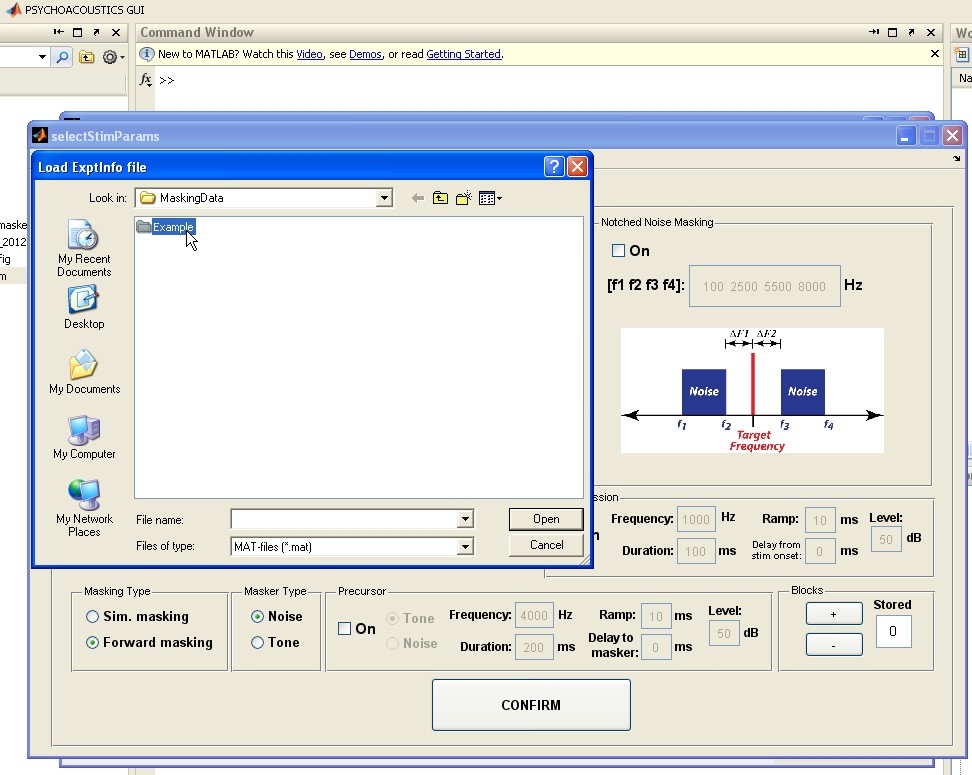
The subjects folder is located in the MaskingData directory and has the same name as the subject ID (default is “subject”) defined when the experiment was created using the registerSubject window.


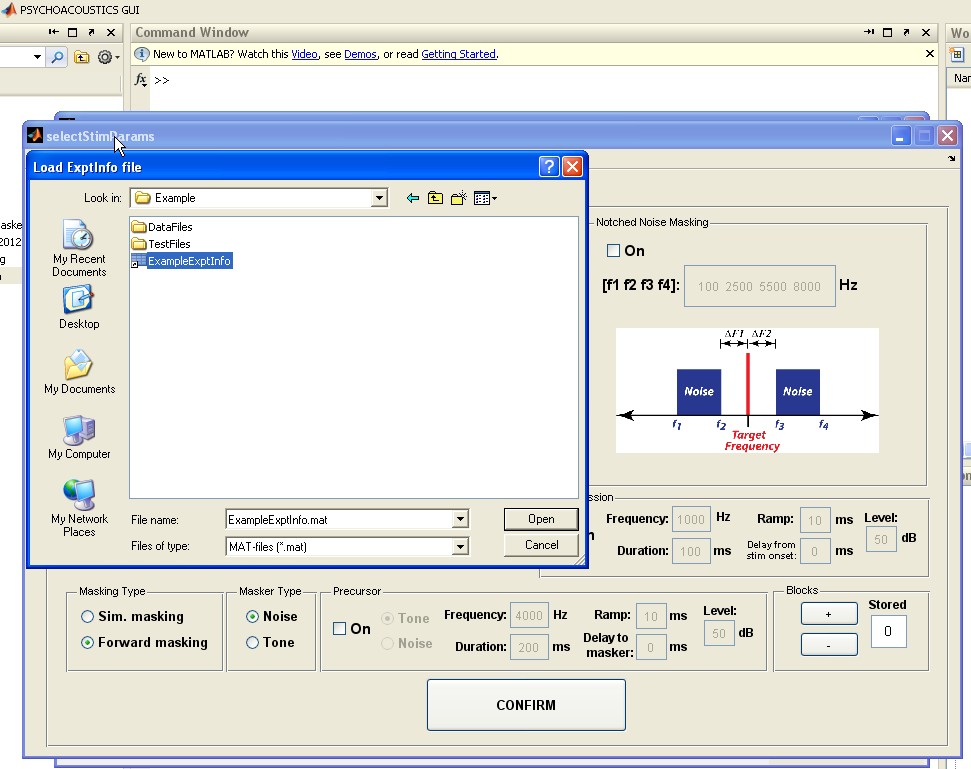


The ExptInfo.mat file is located within the subject’s directory, with the subject’s ID as a prefix to the file name. After the ExptInfo.mat file is located, click “open” in the prompt window. The program will then automatically load the next condition in the experiment and display the time waveform of the stimuli in the MaskingGUI window.

To start a run, selected **RUN 🡪 GO!** from the menu in MaskingGUI. This will open the window that visually displays the observation intervals while the stimuli are played and the collects the subject’s responses.


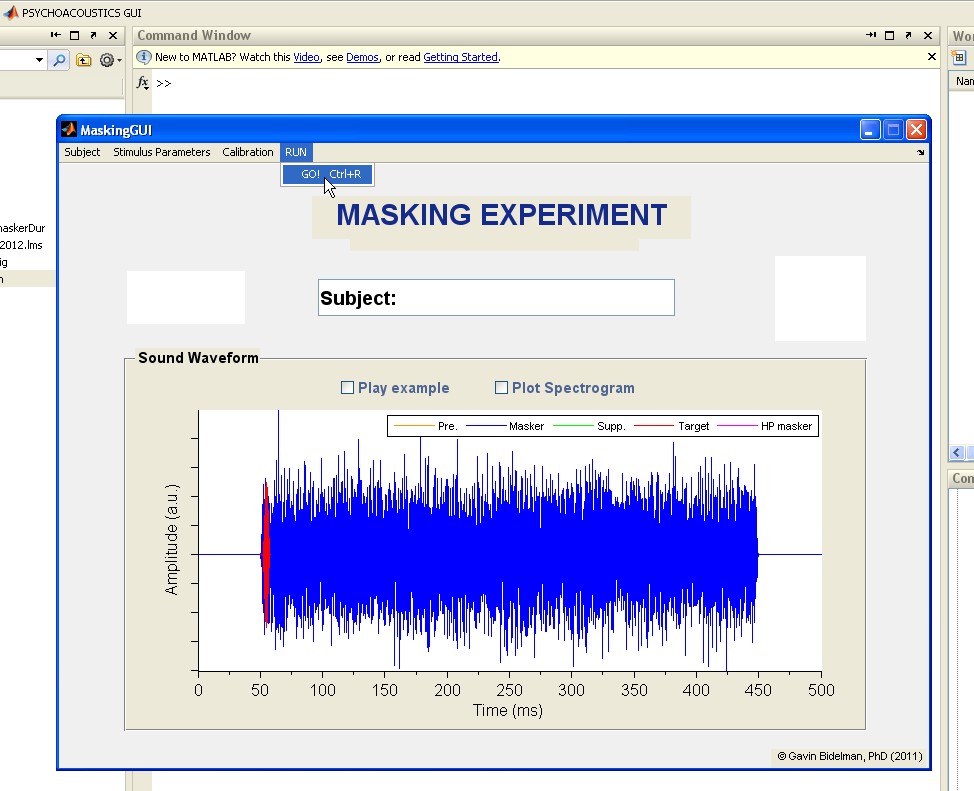


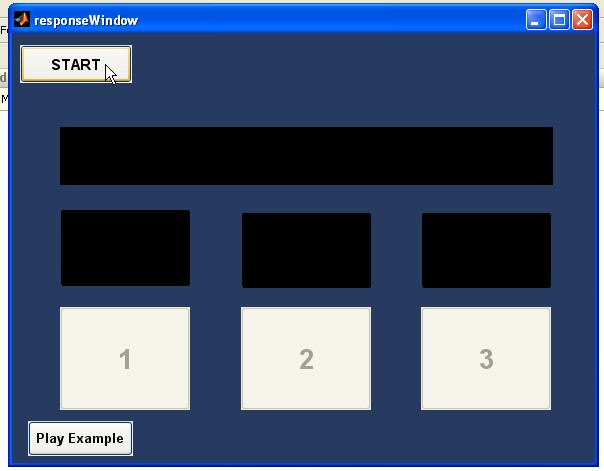
The subject then presses the START button to begin a run.

Information about the current condition is presented in a window titled “runTracker.” More will be discussed about this feature in the section entitled “Features.”


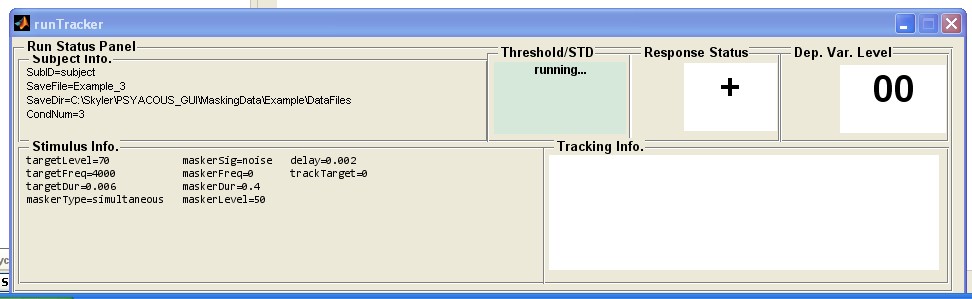


After the START button is pressed, the adaptive three-interval forced-choice procedure will begin where the subject will listen and respond to several trials before a threshold is obtained. During a run, the response window will display the word “running” in the upper left corner.


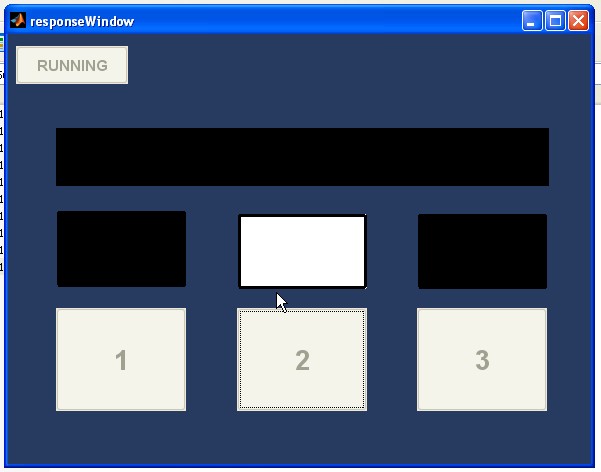


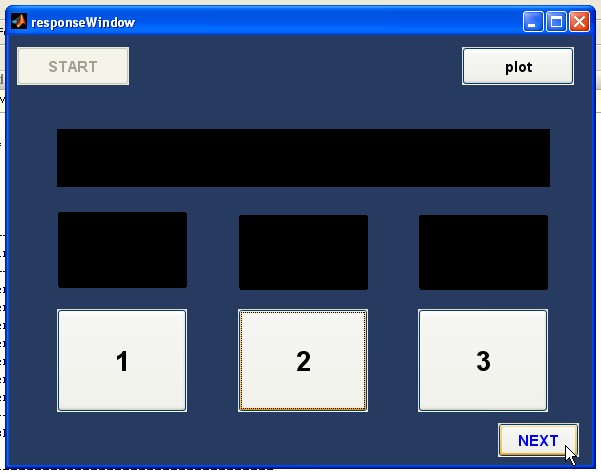

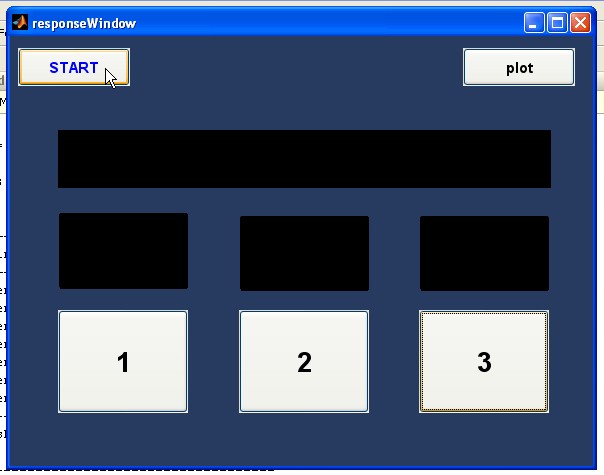
After a run is finished, the START button will reappear in blue, prompting the user to start the second run of the condition. When the second run is completed, the START button will be deactivated and a button labeled “NEXT” will appear in the bottom right-hand side of the window. When the subject presses this button, the next condition of the experiment will be loaded automatically. Subjects should be encouraged to take breaks only when the START button is enabled. If the START button is deactivated or displays “running,” subjects should be encouraged to continue with the task until the START button appears and is enabled. When all conditions are finished, a small window will appear displaying the phrase “You have completed all conditions!” After the subject presses the OK button in this window, the program will save the remaining data and reset.


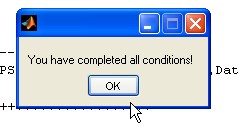


# Adding a condition to an existing experiment:

To add a condition to an existing experiment, first define the condition using the selectStimParams window, then select **File🡪add Condition to Expt** in the menu of the selectStimParams window.


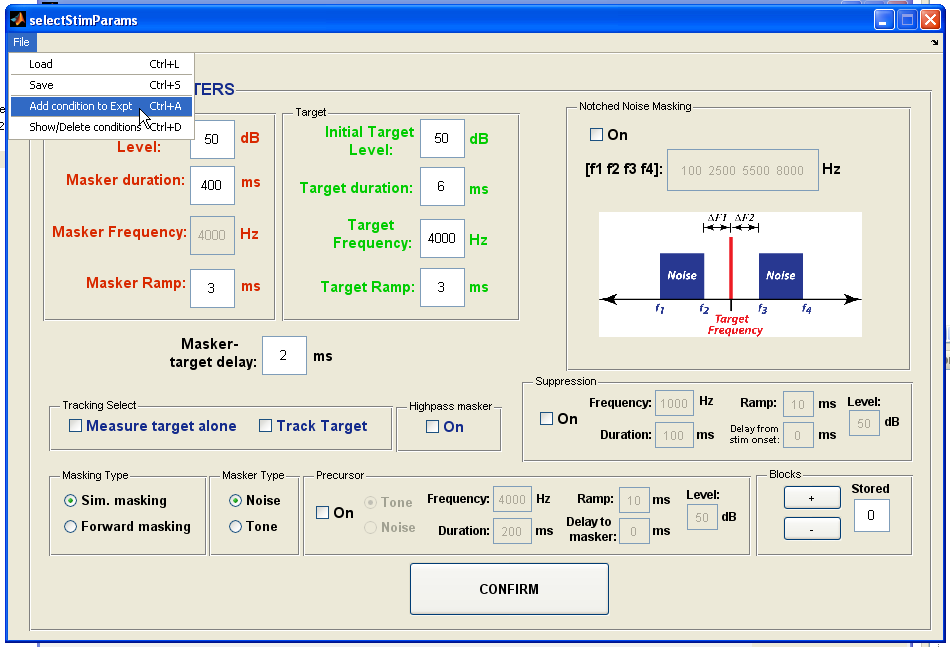


Define conditions here…

The user is then prompted to locate the subject’s directory where the condition is to be added. Once the directory is located, the user then selects the ExptInfo.mat file and message is displayed to show that the condition was displayed.


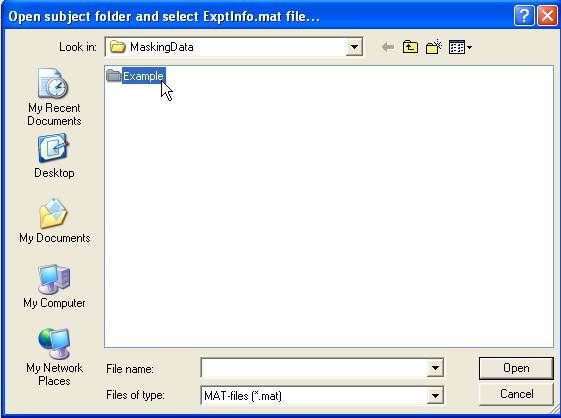


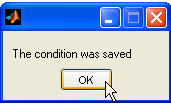

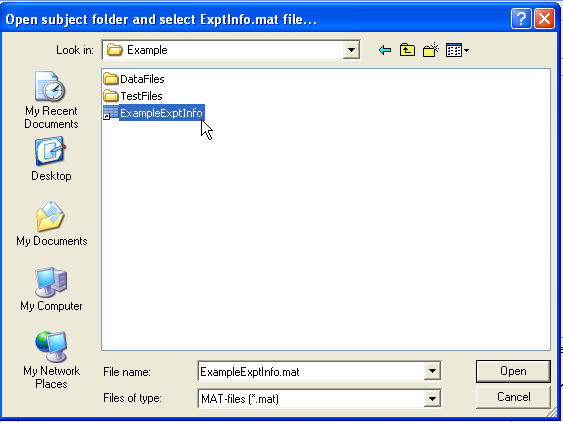


# Removing conditions from an experiment:

To remove conditions from an experiment, **select File 🡪 Show/Delete conditions** in the menu of the selectStimParams window.


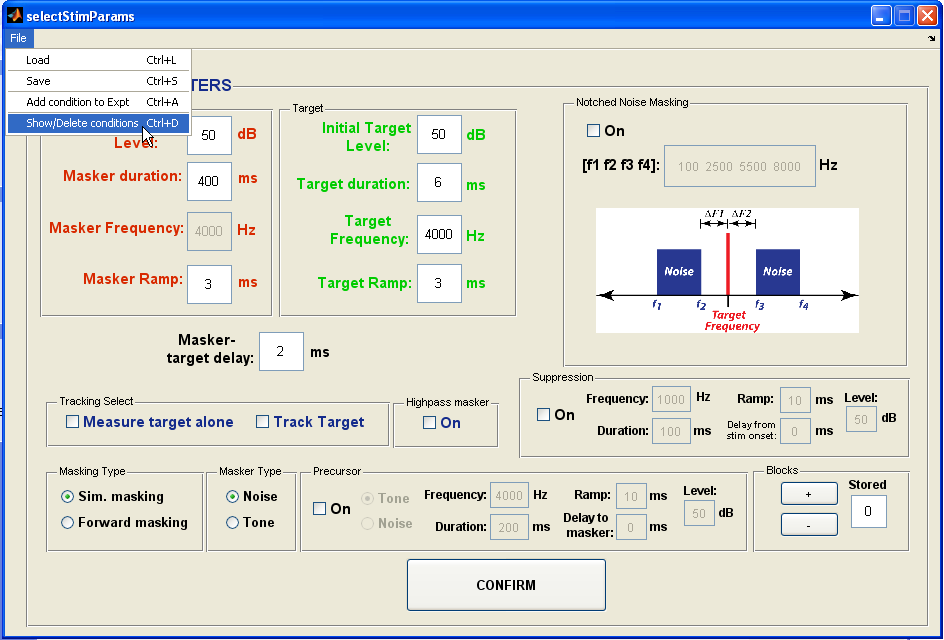


The user is then prompted to locate the subject’s directory where the condition is to be added. Once the directory is located, the user then selects the ExptInfo.mat file and a table showing the current list is displayed.


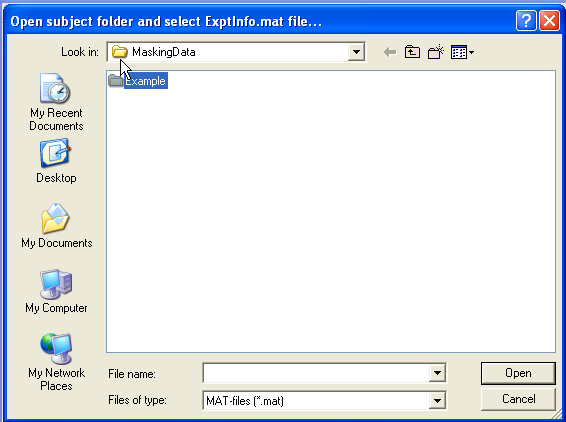


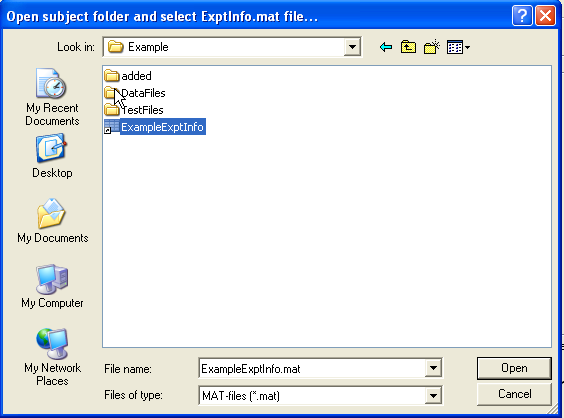


The user is then asked if a condition(s) is to be deleted. If the user selects “No,” the table will continue to be displayed until the user closes the window. If the user selects “Yes,” a message appears indicating how to delete a condition. Specifically, this message indicates that a condition will be deleted if a cell corresponding to a condition is left-clicked and then right-clicked.


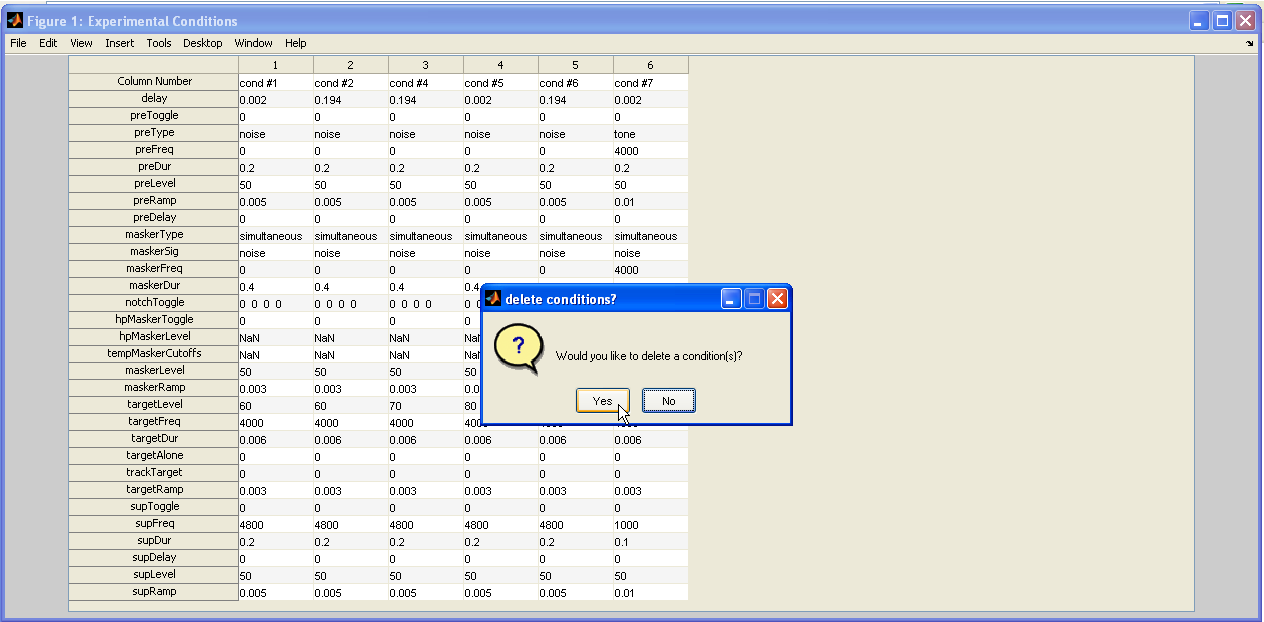


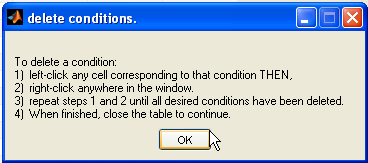


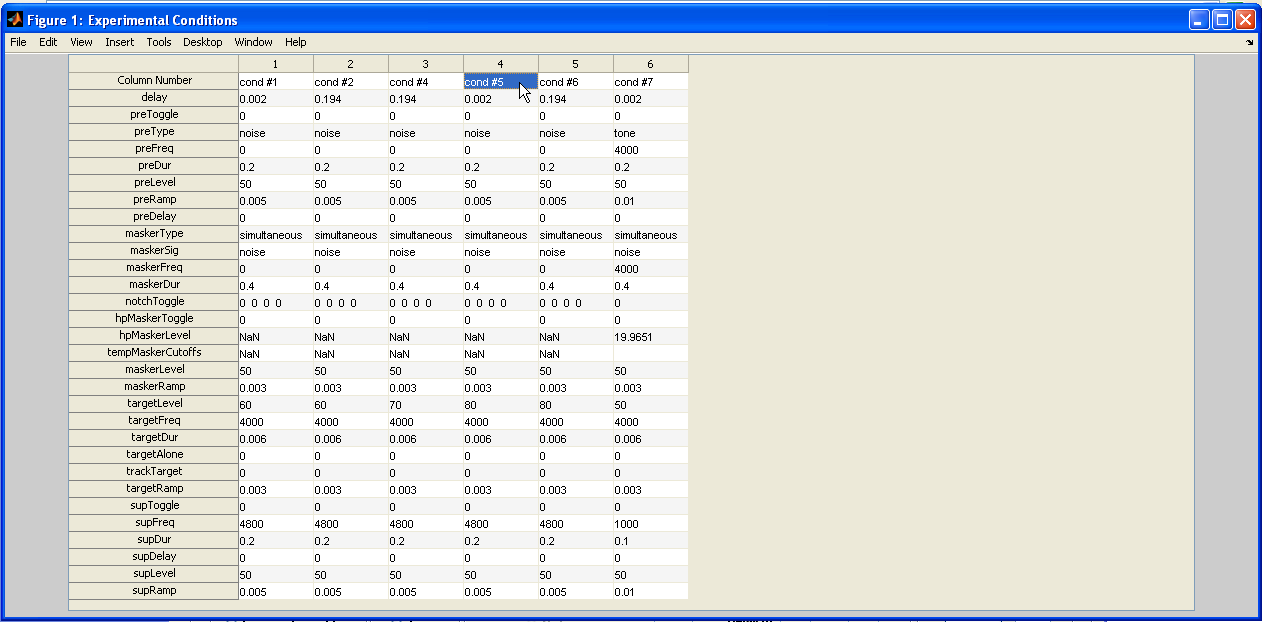
Below is an example of a condition being removed from the experimental list of conditions. Notice that in the first figure condition #5 appears, but in the second it is deleted. The user can continue to delete conditions until all desired conditions have been deleted, after which, the user closes the window.


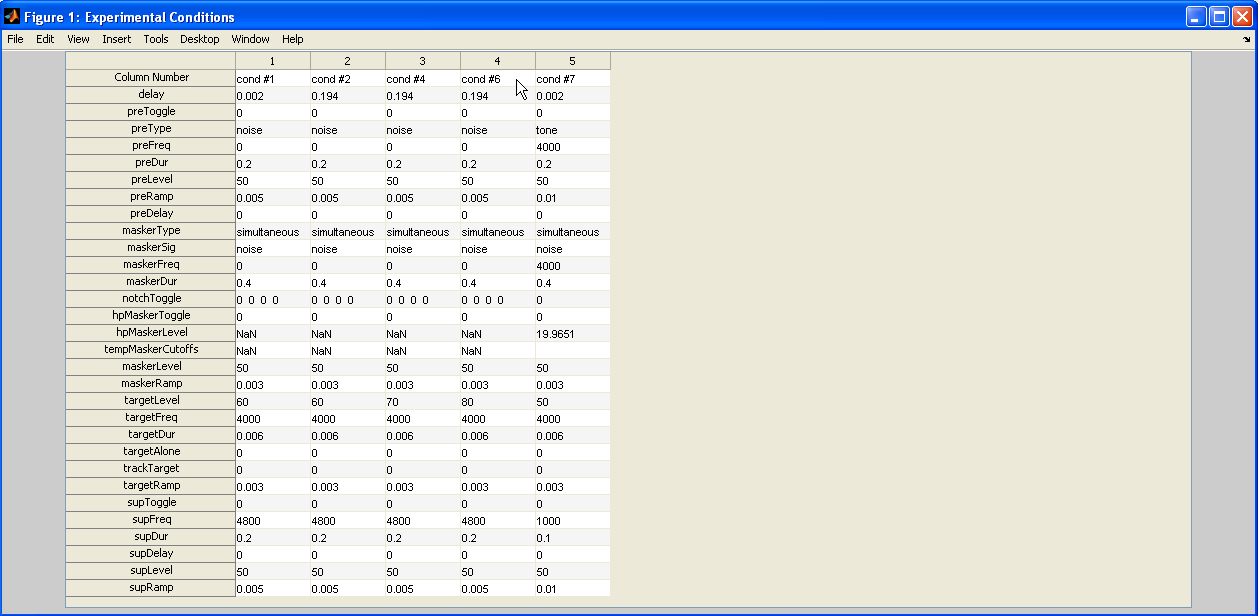

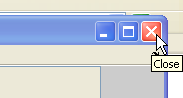


# Calibration:

The MaskingGUI program has a calibration feature where a 1 kHz tone is played at a level defined in the MaskingGUI.m script according to the variable named “CaldB.” The value of CaldB represents the maximum output of the program and should be adjusted for each experimental system using calibration equipment to ensure that distortion is within acceptable limits and the sound pressure level measure during calibration is the same as CaldB.

To play the calibration tone, select **Calibration 🡪 Play calibration tone…** on the menu of the MaskingGUI window. The calibration tone will play for several seconds and then turn itself off. After calibration, a criterion voltage range can be specified in the MaskingGUI.m script and then displayed when selecting to play the calibration tone (in the example, this range is between 42.66-67.60 mV).

#
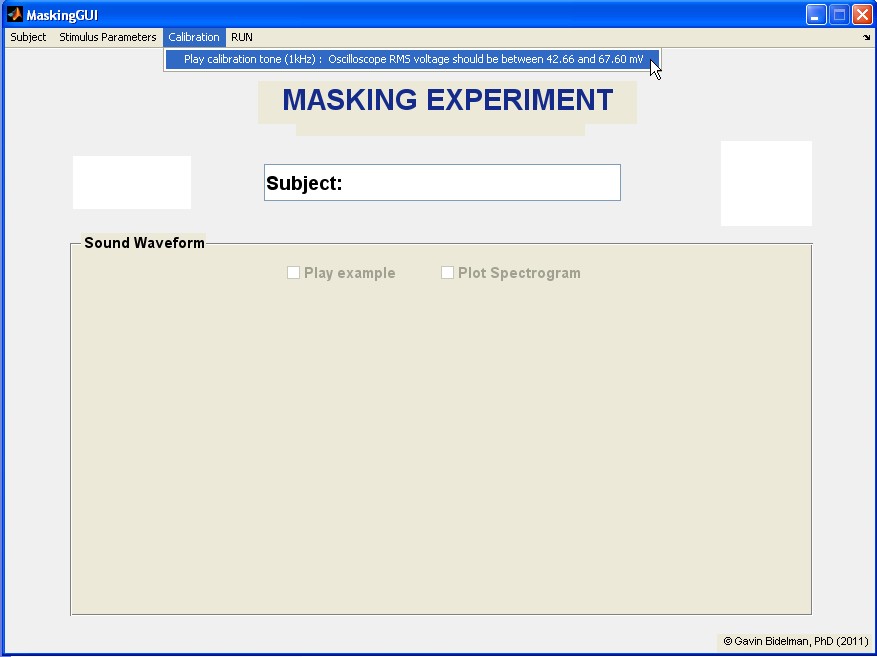


# Features:

## Playing an Example:

Prior to the start of a run, a button appears at the bottom of the response window that allows the subject to hear an example of the condition to be run. To start the example, the user pushes this button and a “dummy” response window appears in which the user then presses the button labeled “START EXAMPLE.”


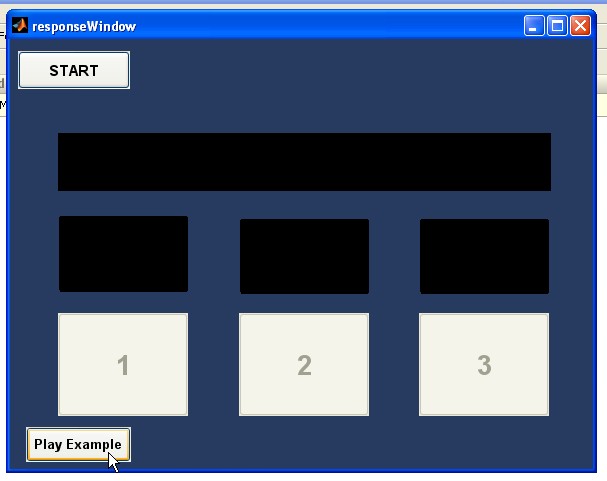


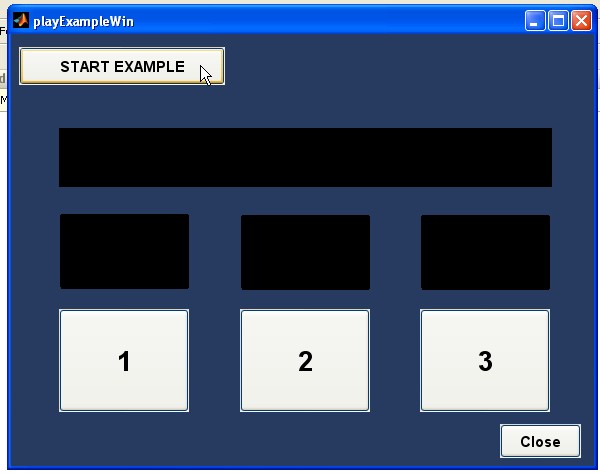


A example then starts where a dummy trial is repeated until the user touches or clicks on the same button used to start the example. For a given repetition of the dummy trial, the program indicates which interval contained the target. After the subject stops the example, buttons are displayed to make the task easier or harder. The subject can click these buttons and restart the example or click close.


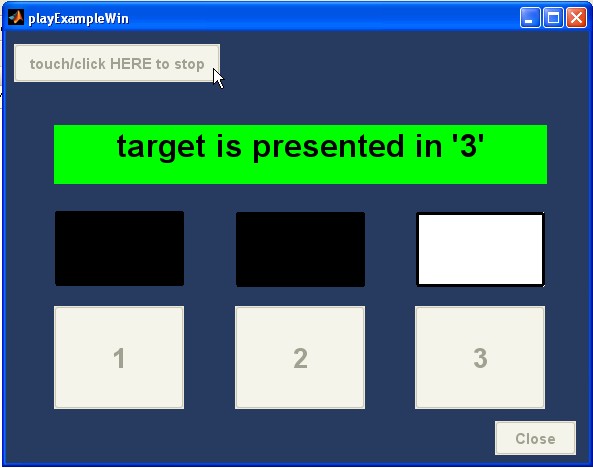


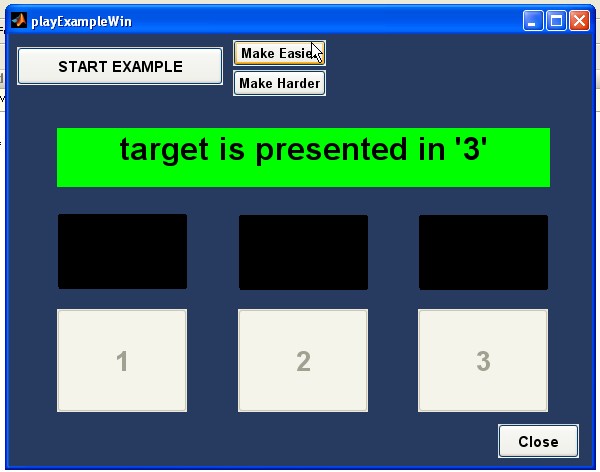


## runTracker:

The runTracker window is displayed while a subject is completing a run. Information regarding the subject, stimuli, response and current masker/signal level are provided in addition to a history of responses and masker/signal levels. At the end of a run, the threshold and standard deviation values are displayed in the box “Threshold/STD.”


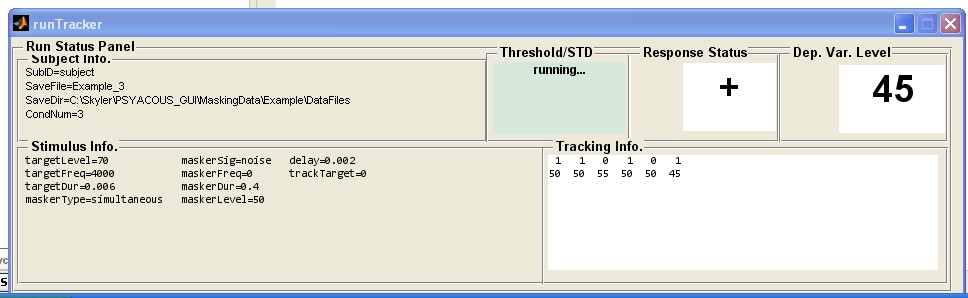


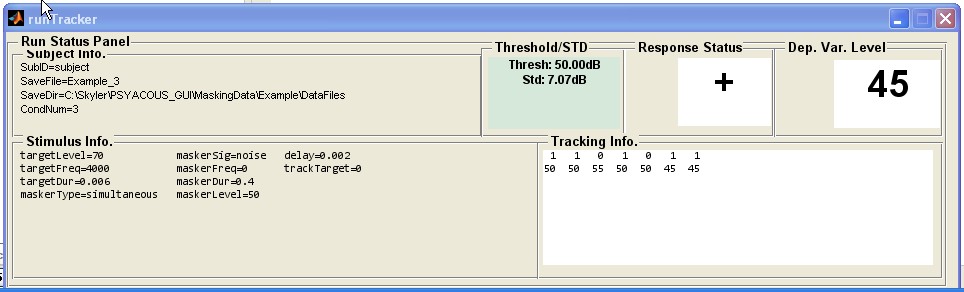

Supplement: Supplementary file 1 [file Data_Sheet_1.ZIP › PsyAcoustX/GUI_HelpFiles/GUI_manual.docx]
