# Supplementary material for: PsyAcoustX: A flexible MATLAB® package for psychoacoustics research
Source: Front Psychol. 2015 Oct 12;6:1498. doi: 10.3389/fpsyg.2015.01498 (PMC4601020; doi:10.3389/fpsyg.2015.01498)

# Increment/Decrement GUI: Quick Guide

**Opening the GUI:**

Open the GUI by running the “psychoacousticsGUI.m” file located in the PSYACOUS_GUI directory, then select the “Increment/Decrement” option from the menu after acknowledging the calibration reminder.

**Registering a subject:**

On the main GUI screen, select the Subject 🡪 New Subject


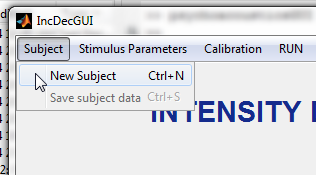


A new window will appear. Enter the subject’s ID in the box labeled “subject,” and click “Done.”


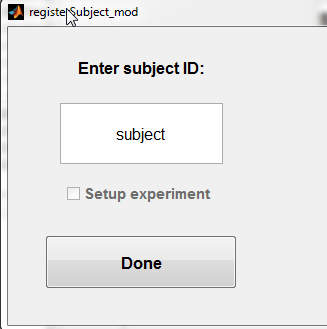


**Defining the Stimulus Parameters:**

On the main GUI select Stimulus Paramters 🡪 Define. A new window will appear (shown below).


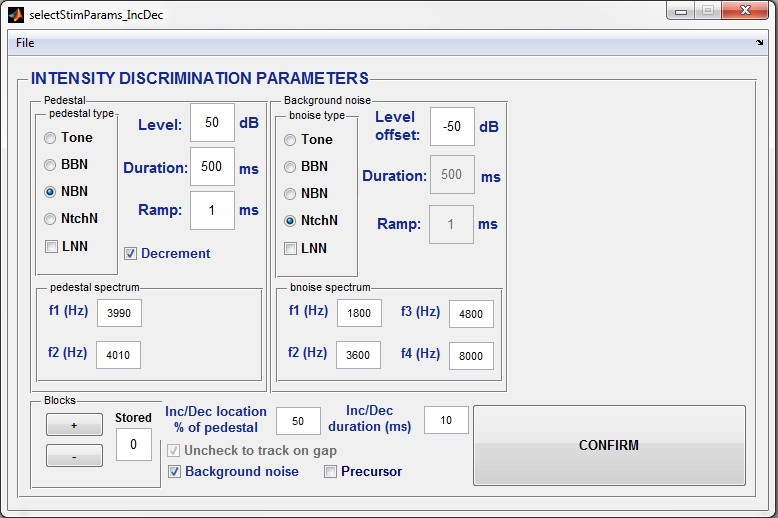


Confirm button

Precursor Panel

Background noise Panel

Blocks Panel

Options

Pedestal Panel

Description of stimulus selection GUI features

*Pedestal Panel:* Used to set properties of the pedestal, including its spectrum, duration, level, and whether the task is increment or decrement detection.

*Background Noise Panel:* For limiting off-frequency listening. This panel allows the user to set the properties of a background noise intended to block off-frequency listening. Typically, the noise will be notched and consist of flanking spectral bands around the pedestal spectrum. The duration and ramp of the background noise is hard-coded to be the same as the duration and ramp of the pedestal.

*Precursor Panel:* Used to set the properties of the precursor (if selected by pressing the “precursor check box” in the options portion of the GUI).

*Blocks Panels:* Allow the user to define a series of conditions. Each time the blocks button is pressed, the stimulus properties displayed in the GUI will be added to a queue of conditions. These conditions will then be run sequentially. If two runs are desired for each condition, press the blocks button twice each time your finish defining stimulus settings for a given condition.

*Options:* The user can turn on or off the background noise or precursor, set the location of the increment/decrement (in percentage from the start of the pedestal) and the duration of the increment/decrement.

*Confirm button:* pressed when all conditions have been defined and the user is ready to start measuring thresholds.

**Saving and loading Stimulus Parameters:**

*Saving:* Select File 🡪 Save on the selectStimParams_IncDec GUI after having set the GUI panels and options to the desired stimulus parameters. A save window will appear. Name your file and click on save.

*Loading:* Select File 🡪 Load. Navigate to the desired file and click open. The saved conditions will appear on the selectStimParams_IncDec GUI. Check to ensure the parameters are correct and then select “CONFIRM” to begin data collection.


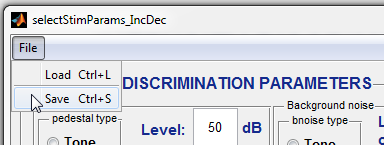

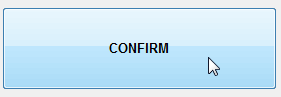

Supplement: Supplementary file 1 [file Data_Sheet_1.ZIP › PsyAcoustX/GUI_HelpFiles/IncDecGUI_QuickGuide.docx]
