# Supplementary material for: PsyAcoustX: A flexible MATLAB® package for psychoacoustics research
Source: Front Psychol. 2015 Oct 12;6:1498. doi: 10.3389/fpsyg.2015.01498 (PMC4601020; doi:10.3389/fpsyg.2015.01498)
Supplement: Supplementary file 1 [file Data_Sheet_1.ZIP › PsyAcoustX/IncDecGUI/Data/Decrement Protocol.docx]

Decrement Protocol

December 2014

Training:

1. Run the training conditions in the order found in the decrement workbook, worksheet titled "Training_2k_Template" ('C:\Skyler\JenningsLabSVN\PSYACOUS_GUI\IncDecGUI\Data\DecrementWorkbook.xlsx')
2. Create a new copy of the decrement workbook and name it 'XX_DEC", where XX are the initials of the subject (OR PRINT OUT THE WORKBOOK, DON'T CREATE A COPY)
3. Register the subject in the GUI
4. When running the decrement GUI, load the training conditions from the folder titled : 'C:\Skyler\JenningsLabSVN\PSYACOUS_GUI\IncDecGUI\Data\dec2014TRAIN'
5. Enter to run 2 repetitions
6. Write down the training thresholds in Training_2k_Template worksheet in the 'XX_DEC workbook.

Experiment

1. Copy the ith column of the 'RandomOrderGenerator" worksheet of the XX_DEC workbook, where "i" is the subject number. Paste this column into the first column of the DEC_Expt_runs1-2 worksheet
2. Run the experiment conditions as indicated by the ORDER column of in the decrement workbook, worksheet titled "DEC_Expt_runs1-2": ('C:\Skyler\JenningsLabSVN\PSYACOUS_GUI\IncDecGUI\Data\DecrementWorkbook.xlsx')
3. Register the subject in the GUI
4. When running the decrement GUI, load the conditions from the folder titled : 'C:\Skyler\JenningsLabSVN\PSYACOUS_GUI\IncDecGUI\Data\ dec2014EXPT
5. Enter to run 2 repetitions
6. Write down the thresholds in the DEC_Expt_runs1-2 in the 'XX_DEC' workbook.
7. Repeat steps 1-5 except with the DEC_Expt_runs3-4 worksheet.
