# Supplementary figures and images for: PsyAcoustX: A flexible MATLAB® package for psychoacoustics research
Source: Front Psychol. 2015 Oct 12;6:1498. doi: 10.3389/fpsyg.2015.01498 (PMC4601020; doi:10.3389/fpsyg.2015.01498)

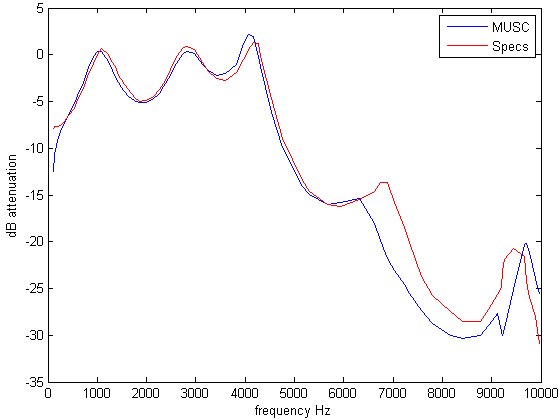

Supplement: Supplementary file 1 [file Data_Sheet_1.ZIP › PsyAcoustX/Cal/MUSC_vs_SPECS.png]

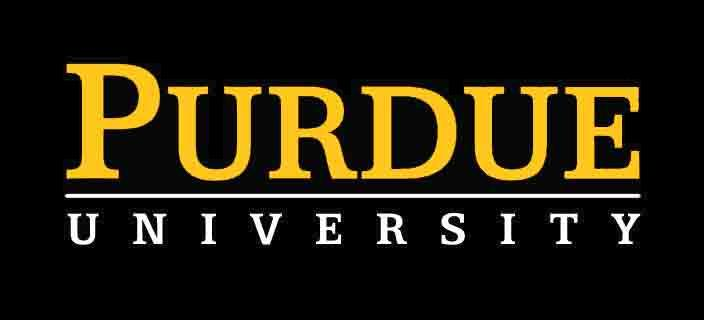

Supplement: Supplementary file 1 [file Data_Sheet_1.ZIP › PsyAcoustX/GapGUI/Interface/purdueSeal.bmp]

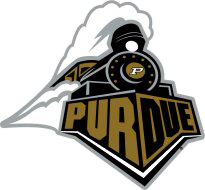

Supplement: Supplementary file 1 [file Data_Sheet_1.ZIP › PsyAcoustX/GapGUI/Interface/purdueTrainLogo.tif]

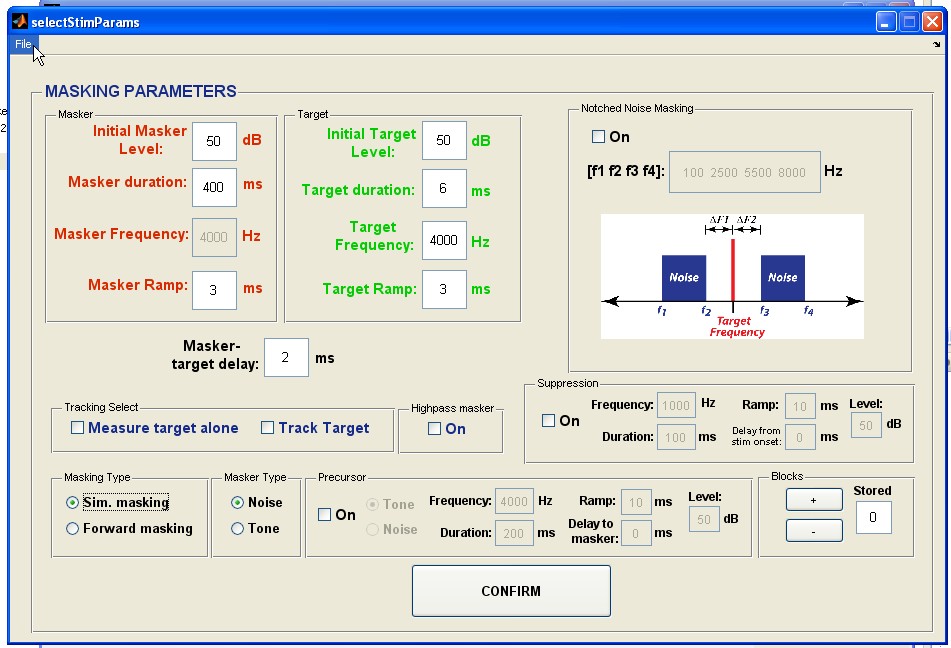

Supplement: Supplementary file 1 [file Data_Sheet_1.ZIP › PsyAcoustX/GUI_HelpFiles/FIGS/AddingConditions2Expt/ScreenHunter_11 Jul. 16 16.35.jpg]

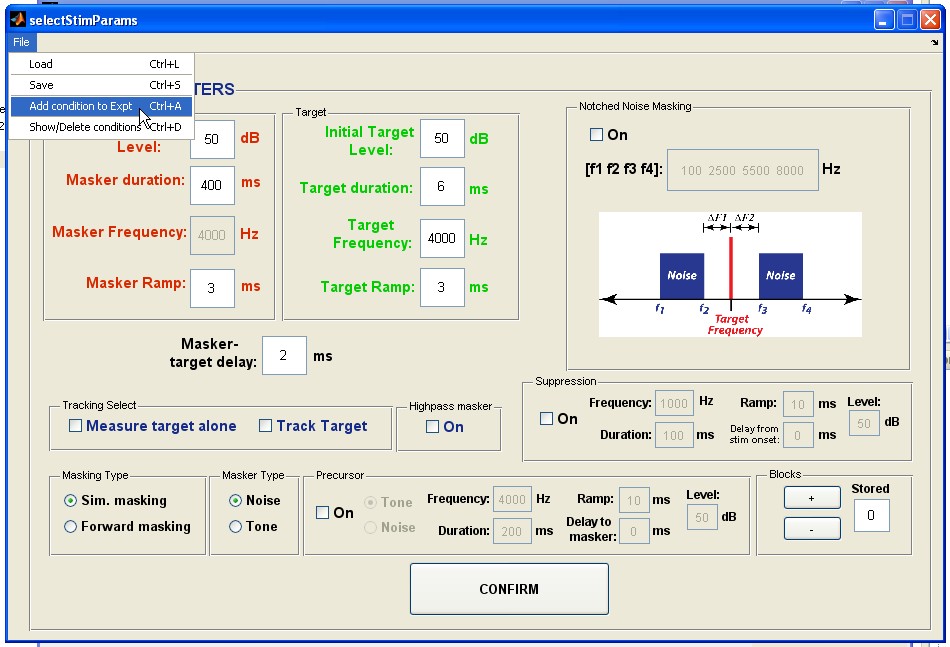

Supplement: Supplementary file 1 [file Data_Sheet_1.ZIP › PsyAcoustX/GUI_HelpFiles/FIGS/AddingConditions2Expt/ScreenHunter_11 Jul. 16 16.36.jpg]

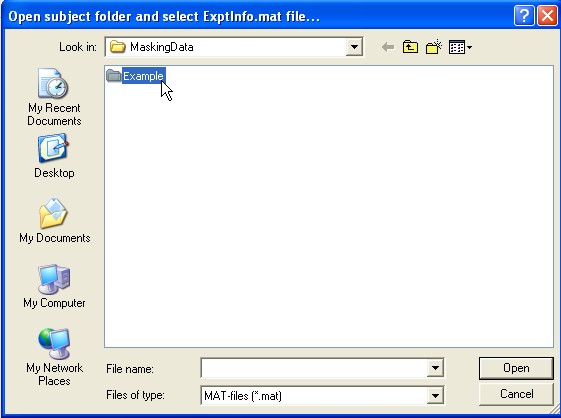

Supplement: Supplementary file 1 [file Data_Sheet_1.ZIP › PsyAcoustX/GUI_HelpFiles/FIGS/AddingConditions2Expt/ScreenHunter_12 Jul. 16 16.36.jpg]

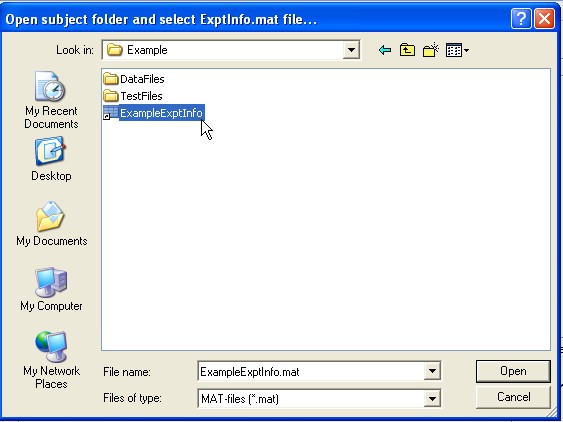

Supplement: Supplementary file 1 [file Data_Sheet_1.ZIP › PsyAcoustX/GUI_HelpFiles/FIGS/AddingConditions2Expt/ScreenHunter_13 Jul. 16 16.36.jpg]

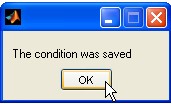

Supplement: Supplementary file 1 [file Data_Sheet_1.ZIP › PsyAcoustX/GUI_HelpFiles/FIGS/AddingConditions2Expt/ScreenHunter_13 Jul. 16 16.38.jpg]

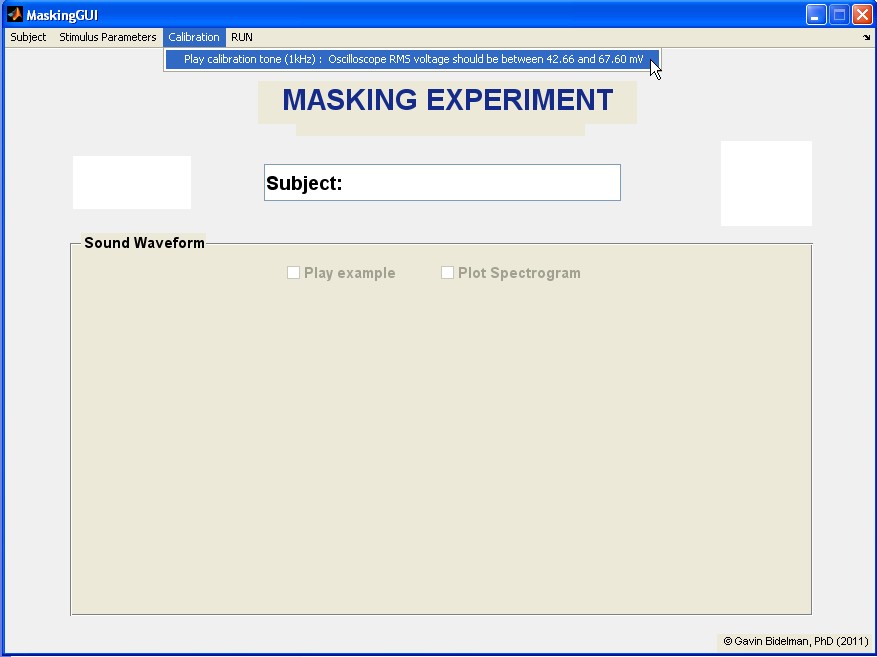

Supplement: Supplementary file 1 [file Data_Sheet_1.ZIP › PsyAcoustX/GUI_HelpFiles/FIGS/Calibration/ScreenHunter_17 Jul. 17 15.48.jpg]

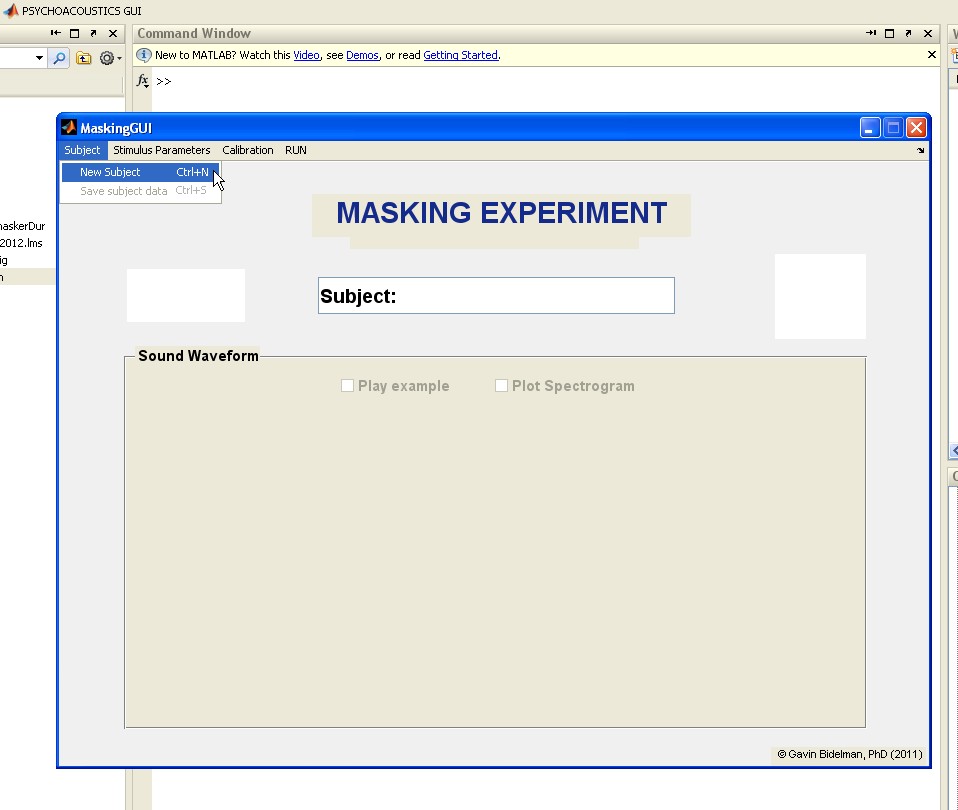

Supplement: Supplementary file 1 [file Data_Sheet_1.ZIP › PsyAcoustX/GUI_HelpFiles/FIGS/EnrollingSubject/ScreenHunter_02 Jul. 16 16.03.jpg]

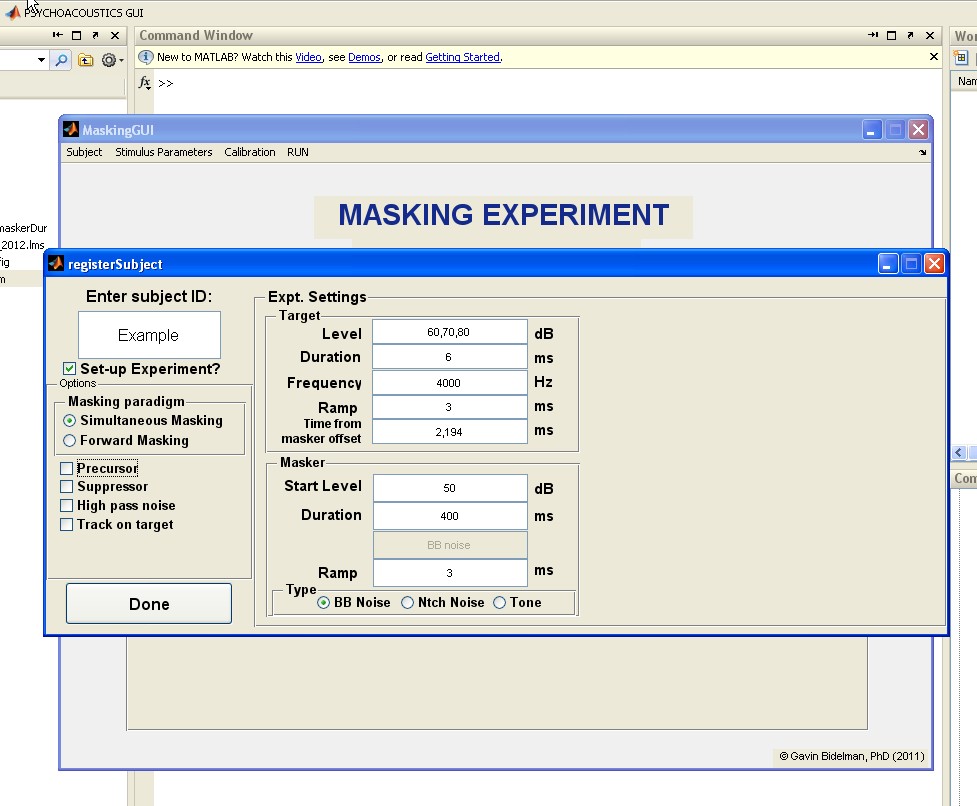

Supplement: Supplementary file 1 [file Data_Sheet_1.ZIP › PsyAcoustX/GUI_HelpFiles/FIGS/EnrollingSubject/ScreenHunter_02 Jul. 16 16.05.jpg]

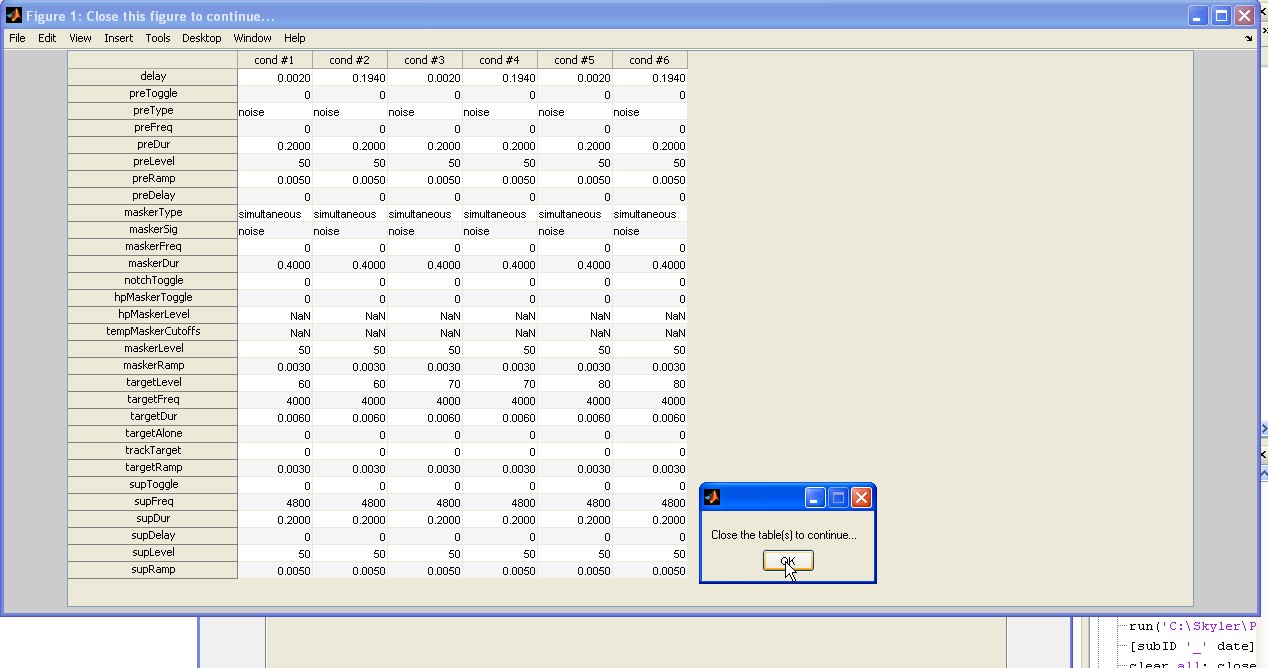

Supplement: Supplementary file 1 [file Data_Sheet_1.ZIP › PsyAcoustX/GUI_HelpFiles/FIGS/EnrollingSubject/ScreenHunter_02 Jul. 16 16.06.jpg]

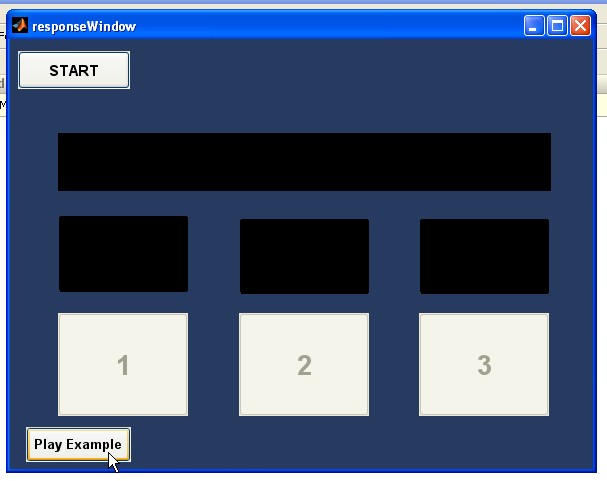

Supplement: Supplementary file 1 [file Data_Sheet_1.ZIP › PsyAcoustX/GUI_HelpFiles/FIGS/Features/PlayingExample/ScreenHunter_07 Jul. 16 16.20.jpg]

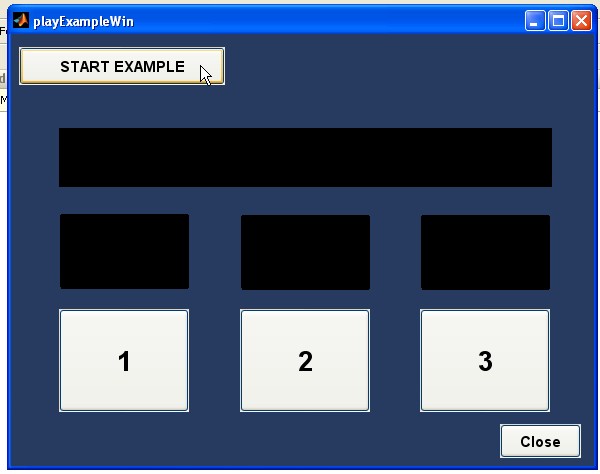

Supplement: Supplementary file 1 [file Data_Sheet_1.ZIP › PsyAcoustX/GUI_HelpFiles/FIGS/Features/PlayingExample/ScreenHunter_08 Jul. 16 16.20.jpg]

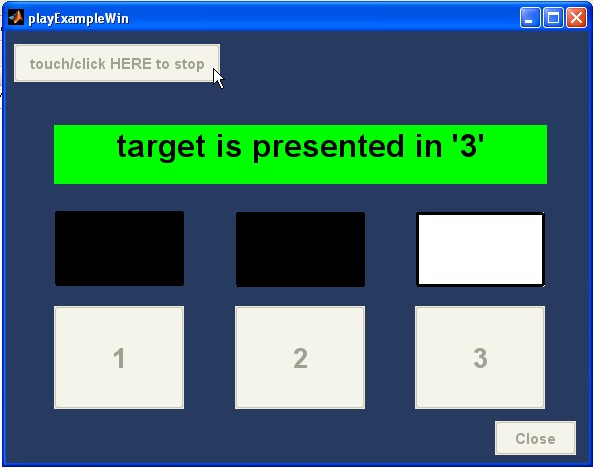

Supplement: Supplementary file 1 [file Data_Sheet_1.ZIP › PsyAcoustX/GUI_HelpFiles/FIGS/Features/PlayingExample/ScreenHunter_08 Jul. 16 16.21.jpg]

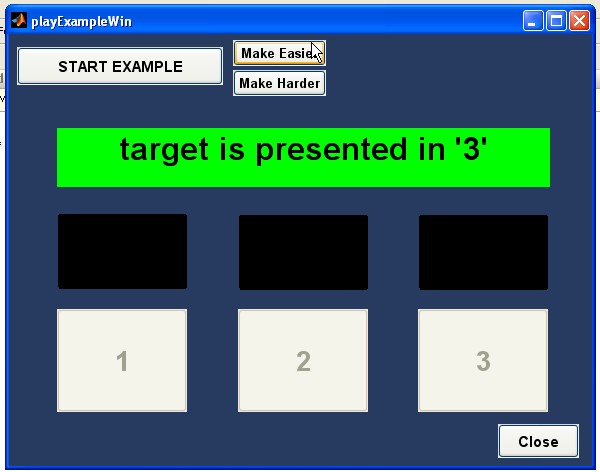

Supplement: Supplementary file 1 [file Data_Sheet_1.ZIP › PsyAcoustX/GUI_HelpFiles/FIGS/Features/PlayingExample/ScreenHunter_08 Jul. 16 16.22.jpg]

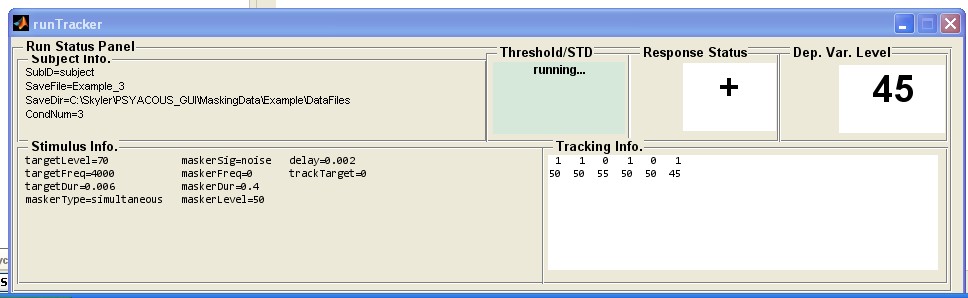

Supplement: Supplementary file 1 [file Data_Sheet_1.ZIP › PsyAcoustX/GUI_HelpFiles/FIGS/Features/runTracker/ScreenHunter_08 Jul. 16 16.25.jpg]

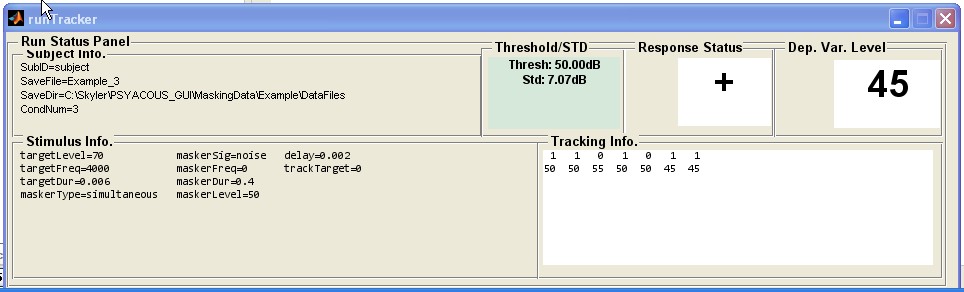

Supplement: Supplementary file 1 [file Data_Sheet_1.ZIP › PsyAcoustX/GUI_HelpFiles/FIGS/Features/runTracker/ScreenHunter_09 Jul. 16 16.25.jpg]

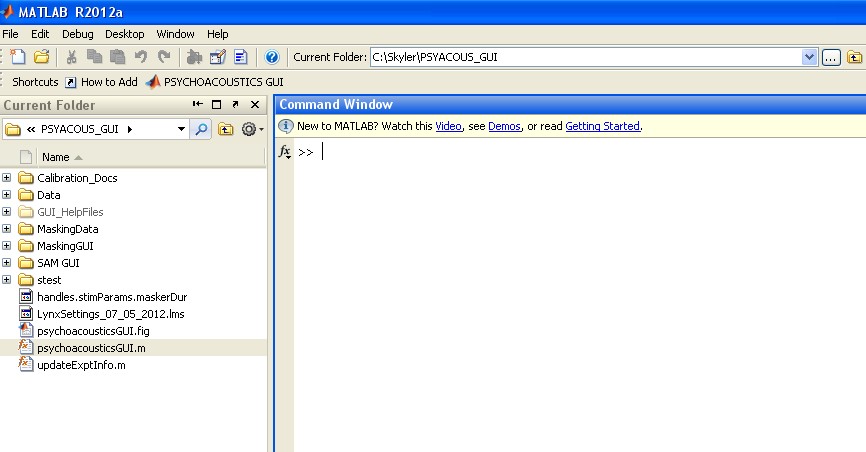

Supplement: Supplementary file 1 [file Data_Sheet_1.ZIP › PsyAcoustX/GUI_HelpFiles/FIGS/OpeningGUI/ScreenHunter_02 Jul. 16 15.58.jpg]

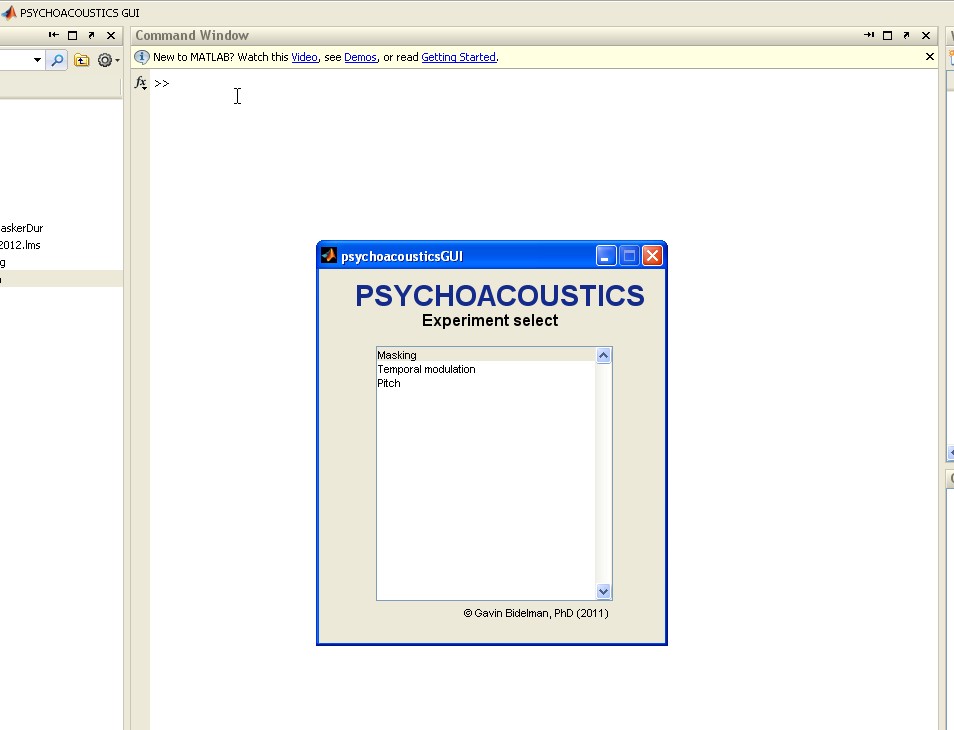

Supplement: Supplementary file 1 [file Data_Sheet_1.ZIP › PsyAcoustX/GUI_HelpFiles/FIGS/OpeningGUI/ScreenHunter_02 Jul. 16 16.01.jpg]

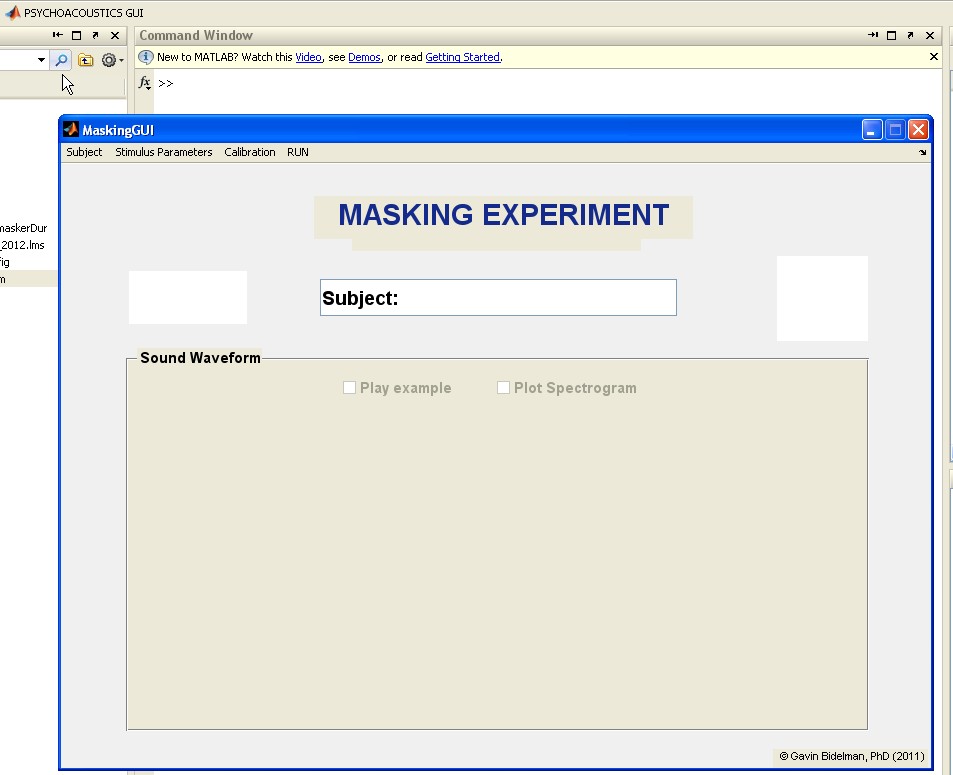

Supplement: Supplementary file 1 [file Data_Sheet_1.ZIP › PsyAcoustX/GUI_HelpFiles/FIGS/OpeningGUI/ScreenHunter_02 Jul. 16 16.02.jpg]

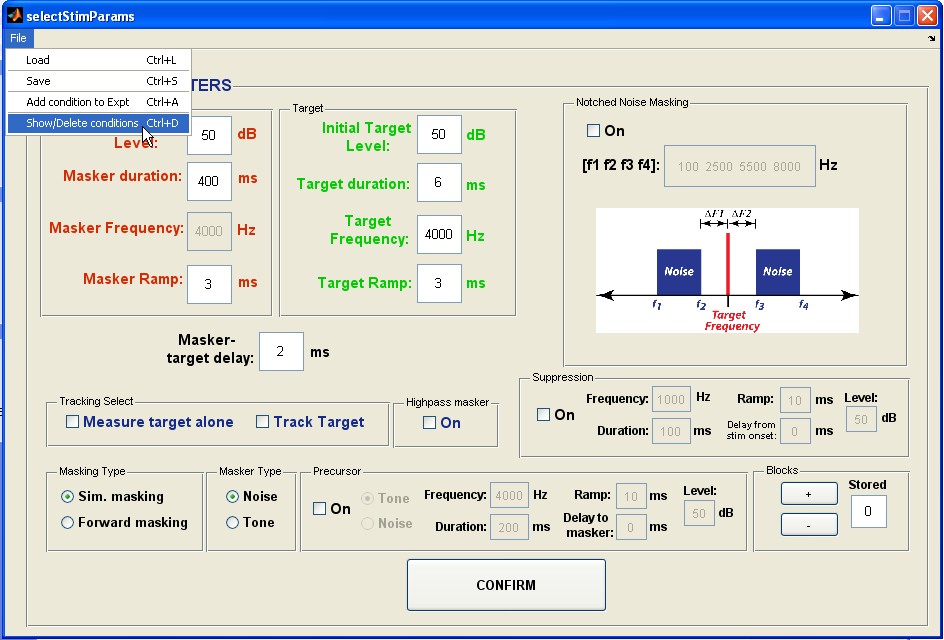

Supplement: Supplementary file 1 [file Data_Sheet_1.ZIP › PsyAcoustX/GUI_HelpFiles/FIGS/RemovingConditions/ScreenHunter_13 Jul. 16 16.38.jpg]

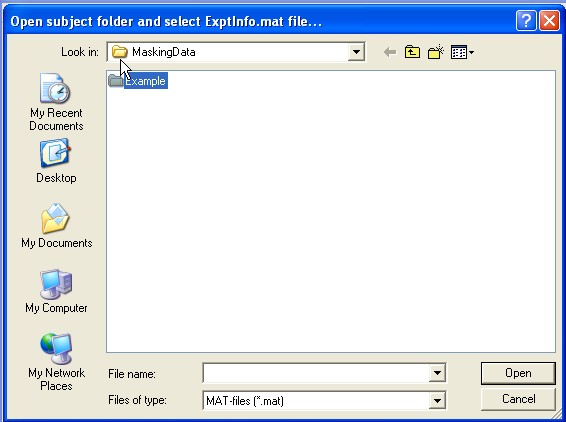

Supplement: Supplementary file 1 [file Data_Sheet_1.ZIP › PsyAcoustX/GUI_HelpFiles/FIGS/RemovingConditions/ScreenHunter_13 Jul. 16 16.39.jpg]

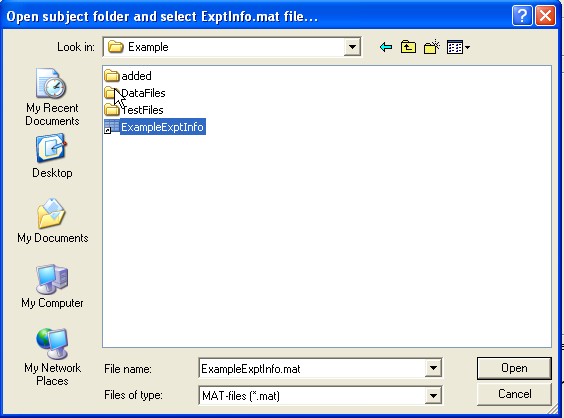

Supplement: Supplementary file 1 [file Data_Sheet_1.ZIP › PsyAcoustX/GUI_HelpFiles/FIGS/RemovingConditions/ScreenHunter_14 Jul. 16 16.39.jpg]

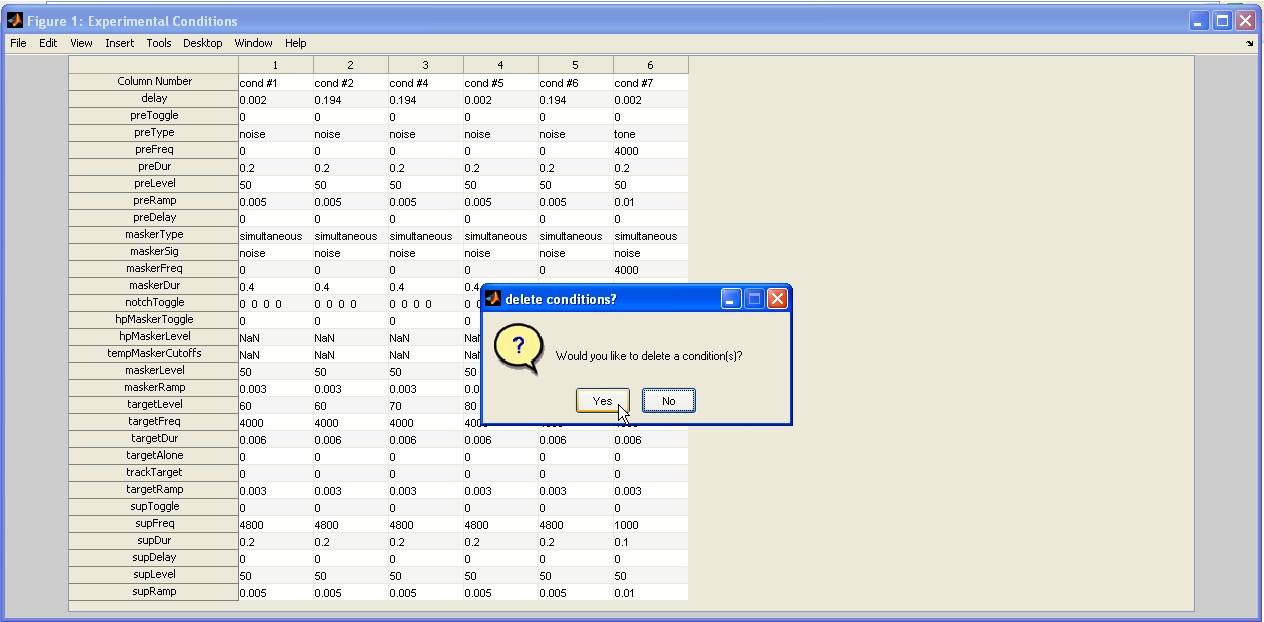

Supplement: Supplementary file 1 [file Data_Sheet_1.ZIP › PsyAcoustX/GUI_HelpFiles/FIGS/RemovingConditions/ScreenHunter_15 Jul. 16 16.39.jpg]

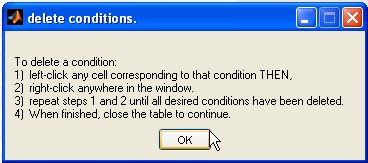

Supplement: Supplementary file 1 [file Data_Sheet_1.ZIP › PsyAcoustX/GUI_HelpFiles/FIGS/RemovingConditions/ScreenHunter_15 Jul. 16 16.40.jpg]

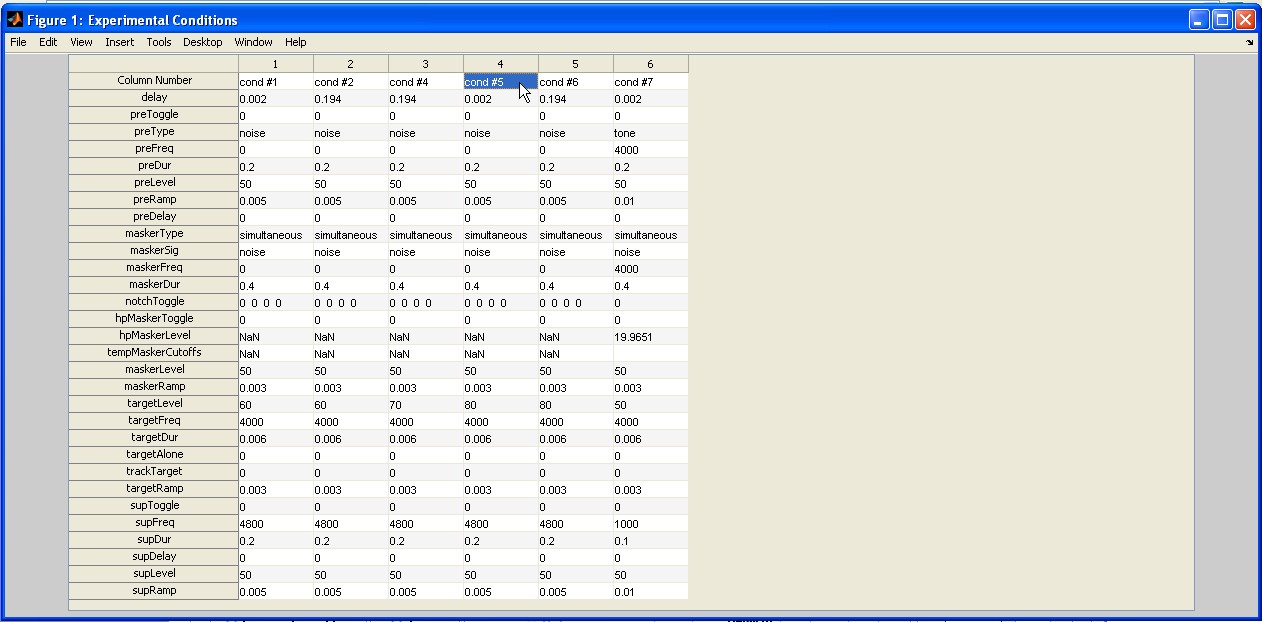

Supplement: Supplementary file 1 [file Data_Sheet_1.ZIP › PsyAcoustX/GUI_HelpFiles/FIGS/RemovingConditions/ScreenHunter_16 Jul. 16 16.40.jpg]

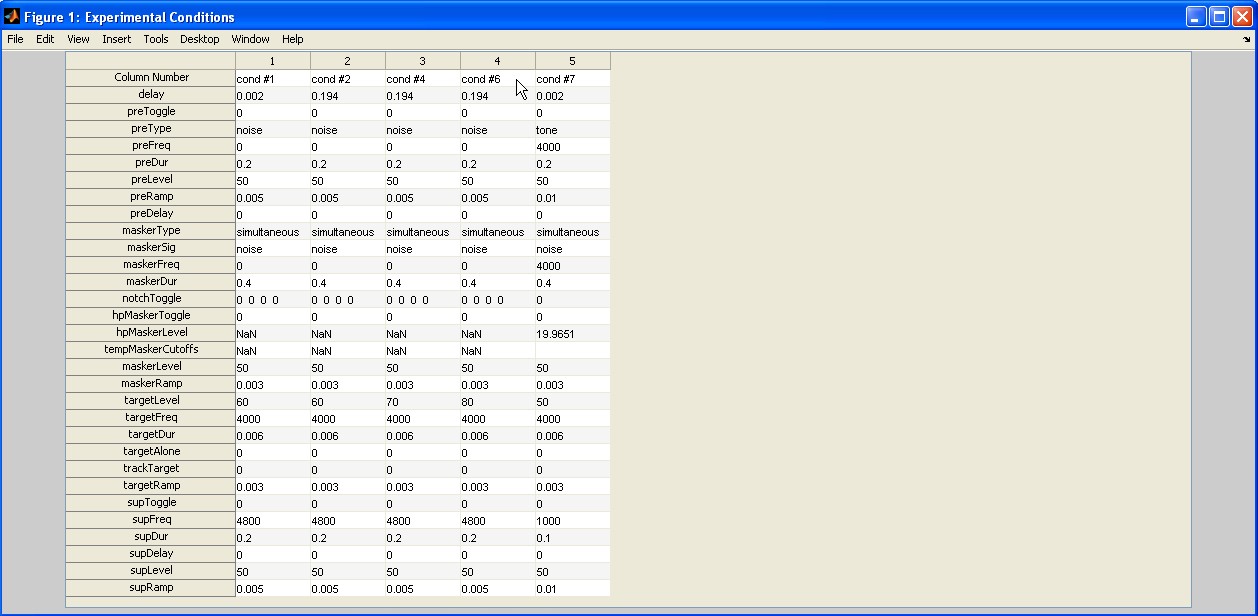

Supplement: Supplementary file 1 [file Data_Sheet_1.ZIP › PsyAcoustX/GUI_HelpFiles/FIGS/RemovingConditions/ScreenHunter_16 Jul. 16 16.41.jpg]

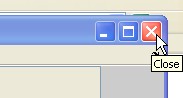

Supplement: Supplementary file 1 [file Data_Sheet_1.ZIP › PsyAcoustX/GUI_HelpFiles/FIGS/RemovingConditions/ScreenHunter_17 Jul. 16 16.41.jpg]

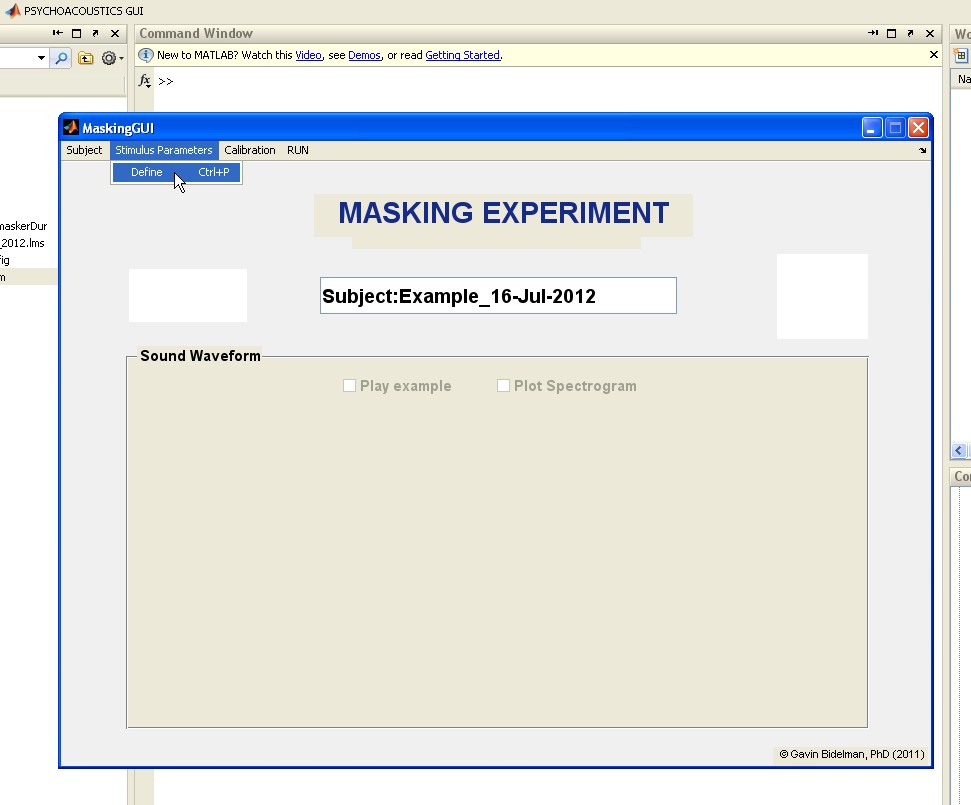

Supplement: Supplementary file 1 [file Data_Sheet_1.ZIP › PsyAcoustX/GUI_HelpFiles/FIGS/RunningSubjects/EnrolledSubject/ScreenHunter_02 Jul. 16 16.11.jpg]

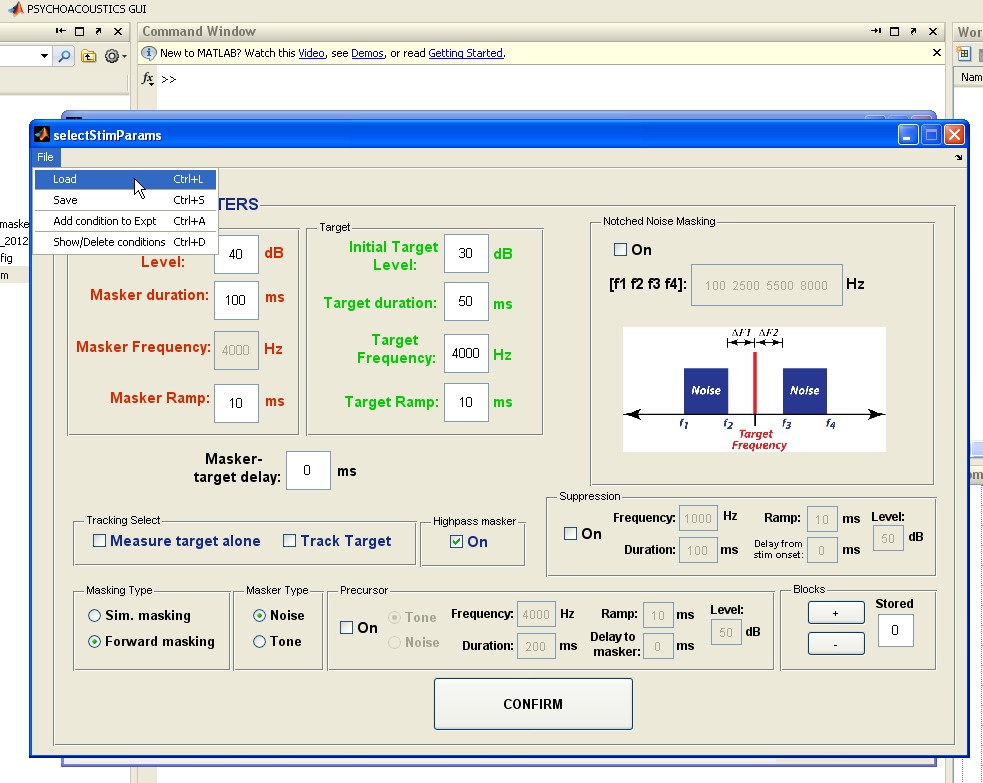

Supplement: Supplementary file 1 [file Data_Sheet_1.ZIP › PsyAcoustX/GUI_HelpFiles/FIGS/RunningSubjects/EnrolledSubject/ScreenHunter_03 Jul. 16 16.11.jpg]

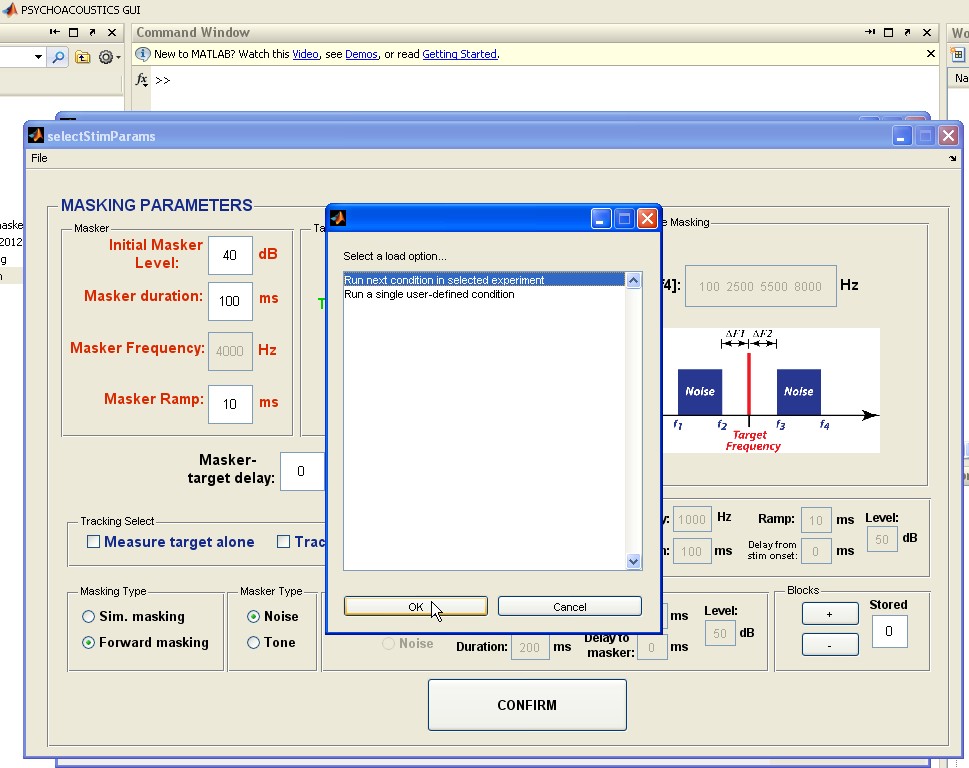

Supplement: Supplementary file 1 [file Data_Sheet_1.ZIP › PsyAcoustX/GUI_HelpFiles/FIGS/RunningSubjects/EnrolledSubject/ScreenHunter_04 Jul. 16 16.11.jpg]

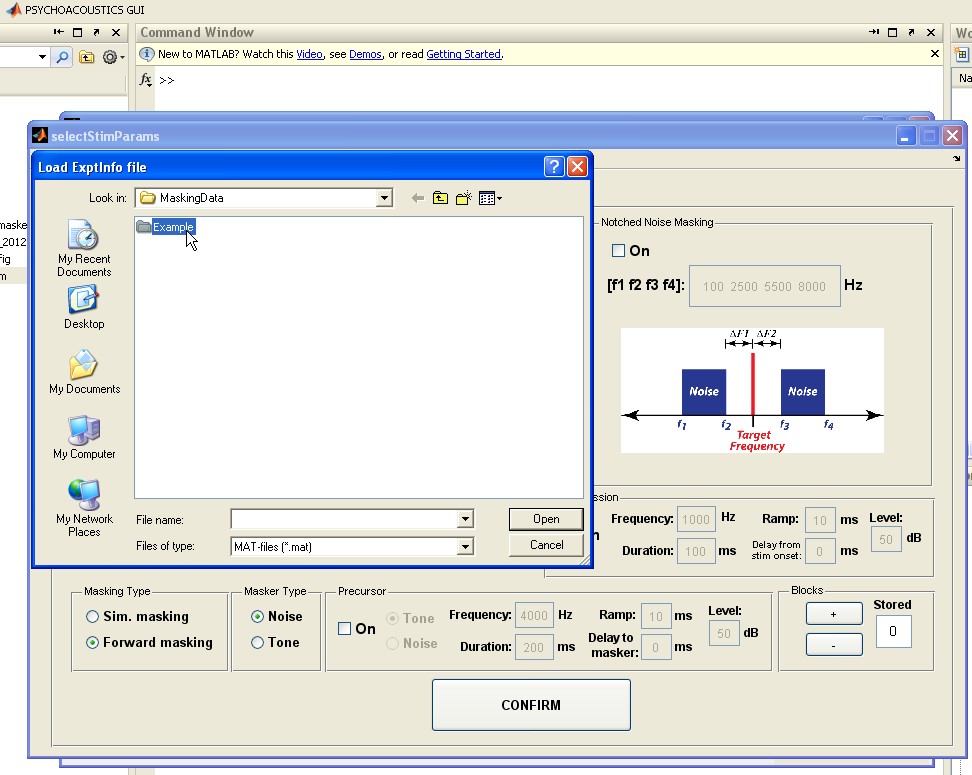

Supplement: Supplementary file 1 [file Data_Sheet_1.ZIP › PsyAcoustX/GUI_HelpFiles/FIGS/RunningSubjects/EnrolledSubject/ScreenHunter_04 Jul. 16 16.14.jpg]

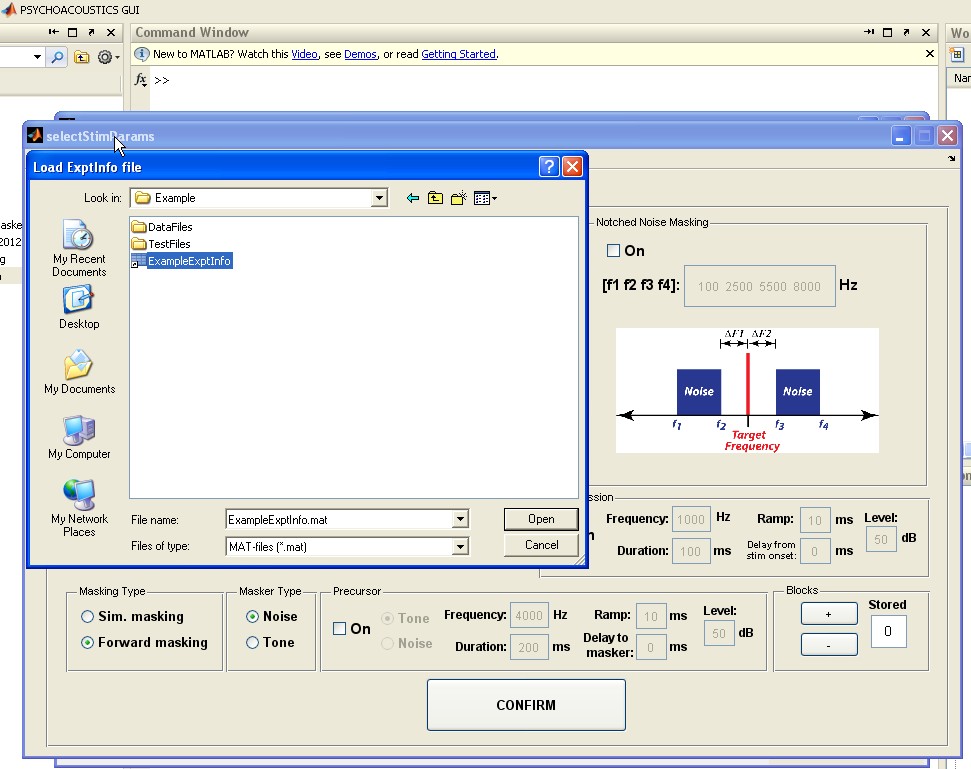

Supplement: Supplementary file 1 [file Data_Sheet_1.ZIP › PsyAcoustX/GUI_HelpFiles/FIGS/RunningSubjects/EnrolledSubject/ScreenHunter_05 Jul. 16 16.14.jpg]

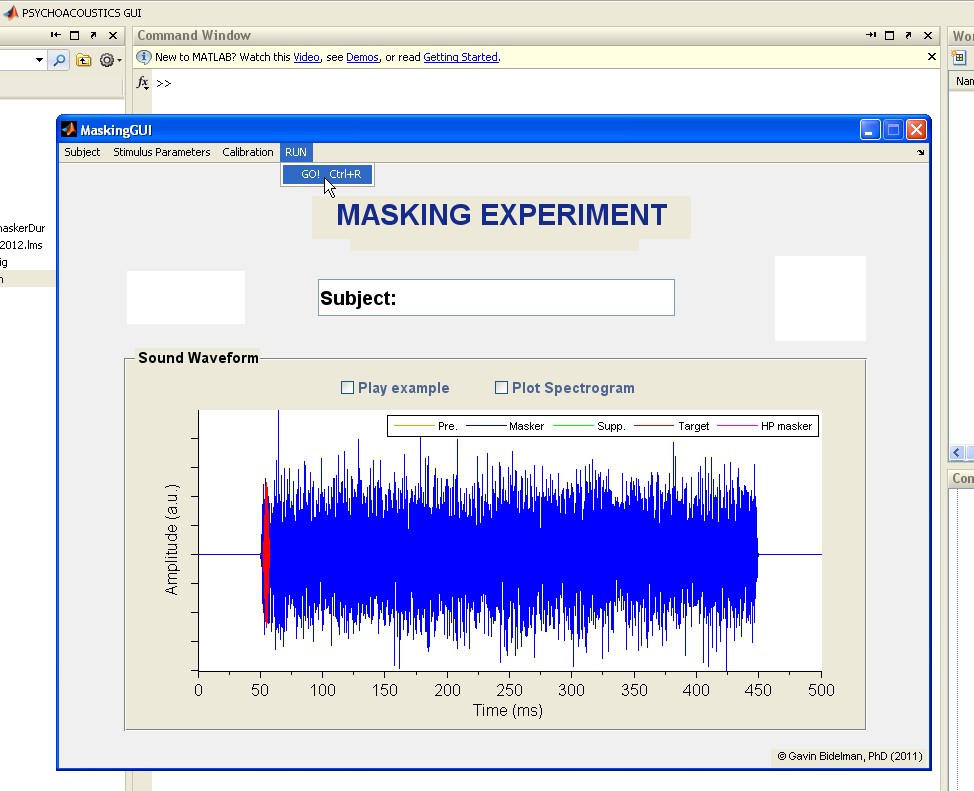

Supplement: Supplementary file 1 [file Data_Sheet_1.ZIP › PsyAcoustX/GUI_HelpFiles/FIGS/RunningSubjects/EnrolledSubject/ScreenHunter_05 Jul. 16 16.15.jpg]

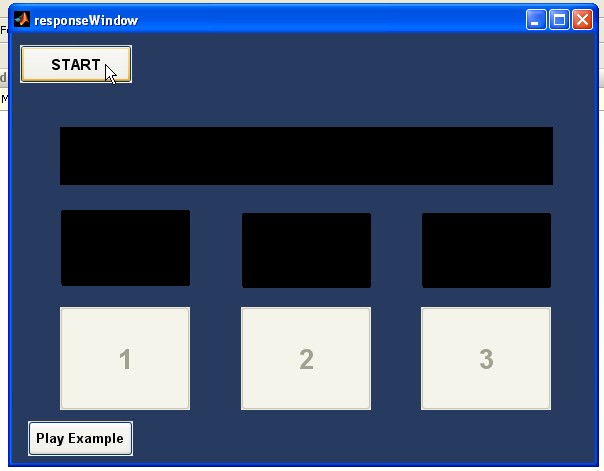

Supplement: Supplementary file 1 [file Data_Sheet_1.ZIP › PsyAcoustX/GUI_HelpFiles/FIGS/RunningSubjects/EnrolledSubject/ScreenHunter_06 Jul. 16 16.15.jpg]

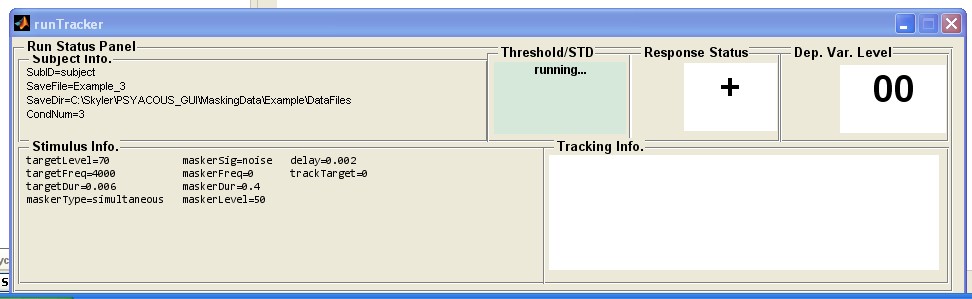

Supplement: Supplementary file 1 [file Data_Sheet_1.ZIP › PsyAcoustX/GUI_HelpFiles/FIGS/RunningSubjects/EnrolledSubject/ScreenHunter_07 Jul. 16 16.15.jpg]

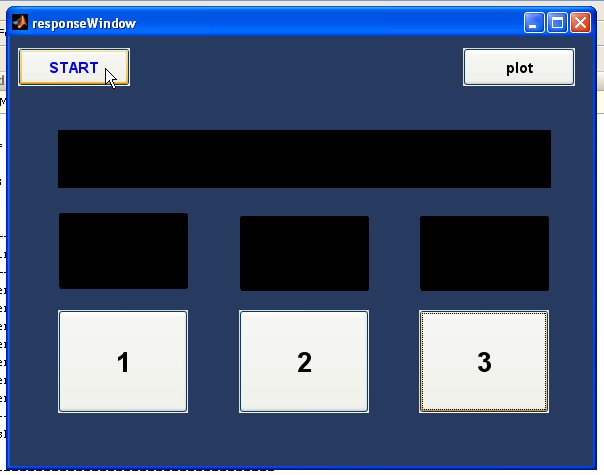

Supplement: Supplementary file 1 [file Data_Sheet_1.ZIP › PsyAcoustX/GUI_HelpFiles/FIGS/RunningSubjects/EnrolledSubject/ScreenHunter_10 Jul. 16 16.26.jpg]

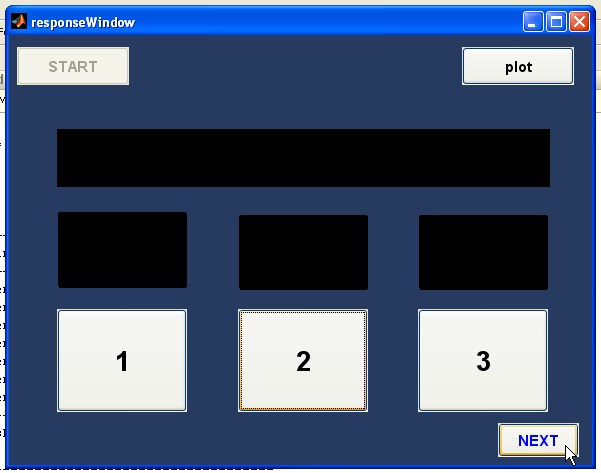

Supplement: Supplementary file 1 [file Data_Sheet_1.ZIP › PsyAcoustX/GUI_HelpFiles/FIGS/RunningSubjects/EnrolledSubject/ScreenHunter_11 Jul. 16 16.26.jpg]

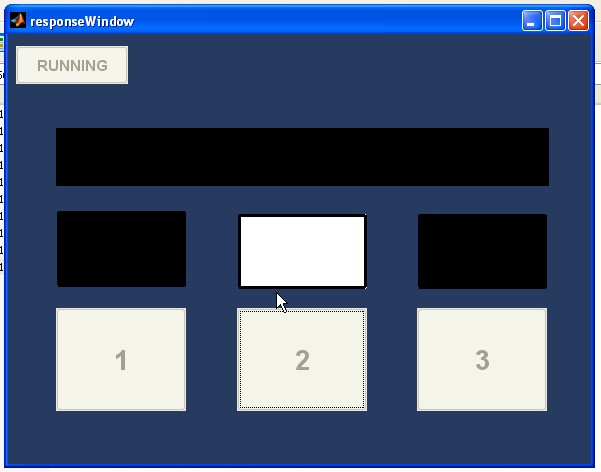

Supplement: Supplementary file 1 [file Data_Sheet_1.ZIP › PsyAcoustX/GUI_HelpFiles/FIGS/RunningSubjects/EnrolledSubject/ScreenHunter_11 Jul. 16 16.29.jpg]

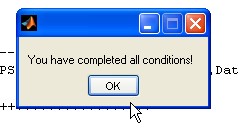

Supplement: Supplementary file 1 [file Data_Sheet_1.ZIP › PsyAcoustX/GUI_HelpFiles/FIGS/RunningSubjects/EnrolledSubject/ScreenHunter_11 Jul. 16 16.32.jpg]
