# Supplementary material for: PsyAcoustX: A flexible MATLAB® package for psychoacoustics research
Source: Front Psychol. 2015 Oct 12;6:1498. doi: 10.3389/fpsyg.2015.01498 (PMC4601020; doi:10.3389/fpsyg.2015.01498)

PsyAcoustX Manual (v1) - 2015

Skyler Jennings, PhD

Contents

Getting started: Experiment Menu and Calibration Opening the GUI: 2

Experiment Menu 2

The SystemInfo.mat file 3

Calibration: 4

Calibration example: 4

Measuring a Threshold with PsyAcoustX 5

Manually defining single or multiple experimental conditions 6

Saving an experimental condition for future data collection 7

Loading a single experimental condition 7

Displaying and saving results 7

Playing an Example: 7

runTracker: 10

Enrolling a new subject in an experiment (masking tasks only): 10

Example experiment using the enroll subject feature 11

Measuring thresholds in quiet at multiple frequencies 12

Running an experiment on an enrolled subject 13

Removing conditions from an experiment: 20

Getting started: Experiment Menu and Calibration

To open the PsyAcoustX program, first open MATLAB and then do one of the following: after setting Matlab's current folder to the PsyAcoustX directory (label #1 below), 1) type "psychoacousticsGUI" at the command line (label #2 below) or drag the function "psychoacousticsGUI.m" to the command window (label #3 below).

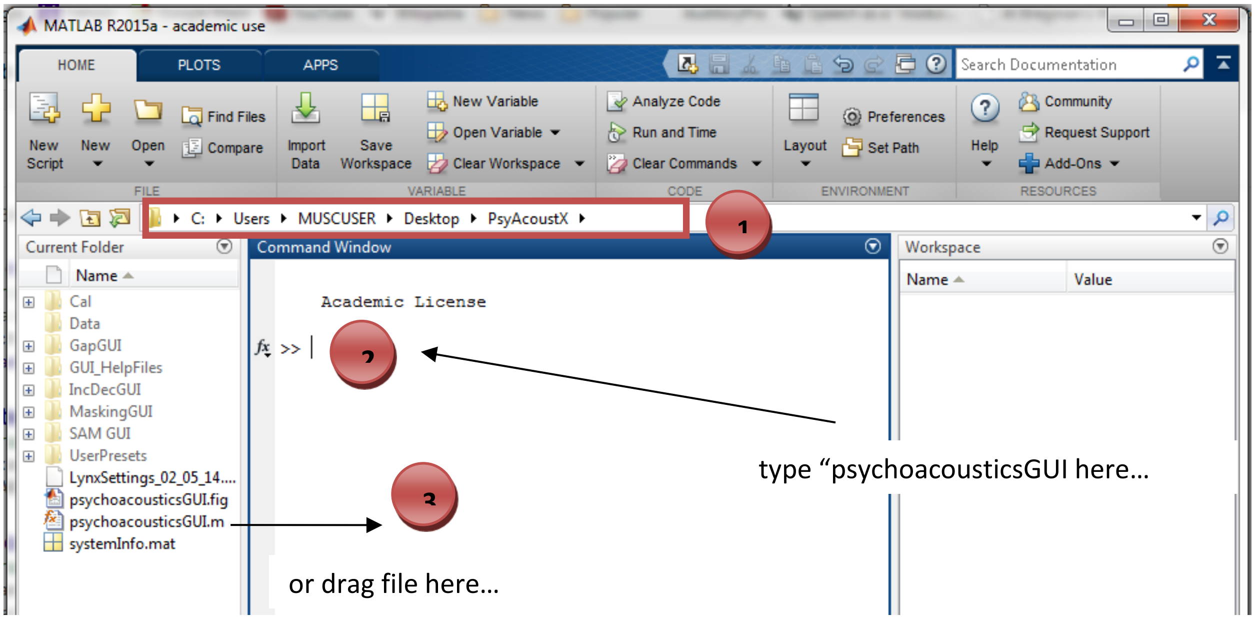

Experiment Menu

After the program has opened, a window will appear at the center of the screen titled "PsycAcoustX" and prompt the user to select a type of experiment. Several perceptual tasks are supported including masking tasks, temporal modulation detection tasks, increment/decrement detection tasks, and gap detection tasks.

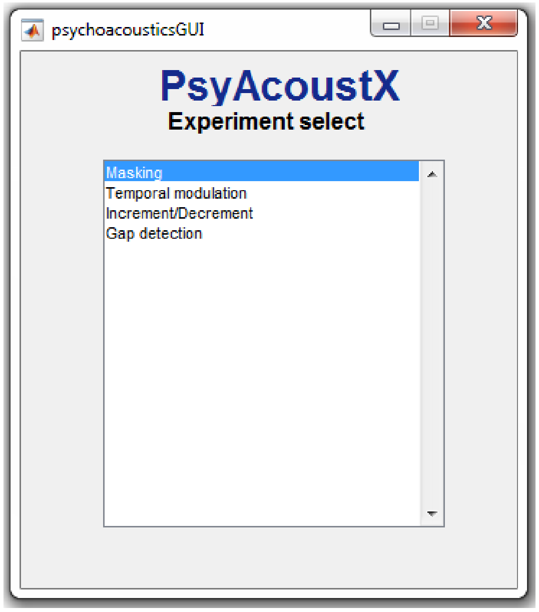

After a task is selected from the PsyAcoustX window, the *home window* (example below) for the selected task will open. The examples that follow involve the *home window* associated with masking tasks. At this point the user has several options including: 1) enrolling a new subject in an experiment involving several conditions (only supported for masking experiments), 2) defining the stimulus parameters for a single user-defined condition, 3) running conditions on an experiment or on a single user-defined condition, 4) deleting conditions from an existing experiment (only supported for masking experiments) or 5) calibrating the laboratory equipment. These options are discussed in the sections that follow.

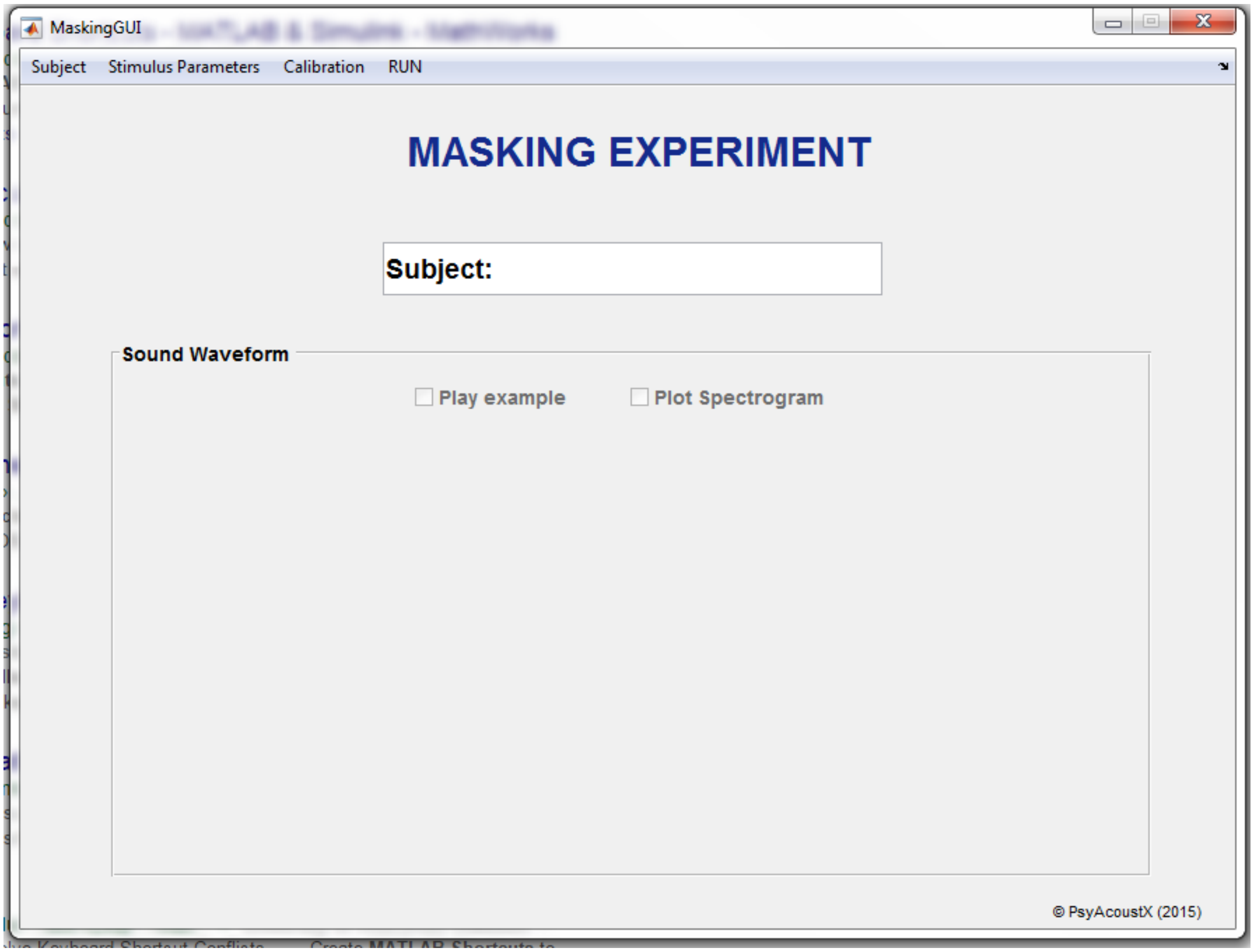

The SystemInfo.mat file

PsyAcoustX can be customize to a particular set of hardware by adjusting variables in the systemInfo.mat file. This file is located in the main PsyAcoustX directory and can accessed by loading in to the workspace via MATLAB's native *load* command, or by simply dragging the file to MATLAB's command window. Once the file is loaded, a variable called "*system*" will appear in the workspace. This variable is stored as a MATLAB structure, with several fields. The fields associated with *system* are:

> *caldB: a numeric value associated with the acoustic output of the system to the 1 kHz calibration tone*
> *calStr: an optional message to assist with daily calibration checks*

phones: a string identifying the headphones being used by the system (e.g., ER2)
Fs: the sampling rate of the system
bits: the number of system quantization bits
mode: used for code development and debugging
hide: used for code development and debugging
calRemind: a boolean indicating whether or not to display a calibration reminder when PsyAcoustX is launched
calRemindStr: a user-defined message displayed when PsyAcoustX is launched

These fields can be edited at the command line. For example, if a system is to be calibrated at 100 dB SPL, the caldB field can be modified by issuing the following statement at the command line:

system.caldB=100;

after this modification, the *system* variable and its fields need to be save in order to update the systemInfo.mat file. To update systemInfo.mat, type the following at the command window:

save('system','systemInfo.mat');

The *system.phones* field is set to "no_correction" by default, meaning that PsyAcoustX will not attempt to correct for attenuation or amplification inherent in the frequency response of the headphones. To allow for such a correction, the user will need to provide the frequency response of the headphones in a .mat file, stored in the \Cal\ directory. Moreover, the user must provide a unique name for these headphones (e.g., ER5A) when naming the .mat file. Finally, the user must update the source code in the dBDiffPhones.m file accordingly.

Note: setting system.phones to something other than "no_correction" is not recommended when using stimuli (including bands of noise) with energy above the cutoff frequency of the headphone's frequency response.

Calibration:

PsyAcoutX has a calibration feature where a 1-kHz tone is played at a level defined in the systemInfo.mat file, according to the variable named system.caldB. The value of CaldB represents the maximum output of PsyAcoustX and should be adjusted for each experimental system using calibration equipment to ensure that distortion is within acceptable limits.

Calibration example:

Calibrating the system can be done with a sound level meter, a coupler, and system headphones/earphones. For daily calibration checks, it is also useful to include a voltmeter or oscilloscope when calibrating to note the electrical output of the calibration tone. The example below uses a Larson-Davis, system 824 sound level meter, a 1-inch Larson-Davis microphone coupled to an AEC 100, and a 2-cc coupler for insert earphones. The system calibration was set to 100 dB SPL, by setting *system.caldB =100*. After checking the calibration of the microphone, EARTONE-5A insert earphones were inserted into the plastic tubing of the 2-cc coupler. Using the *home window* of one of the PsyAcoustX experiments (e.g., masking), *Calibration > Play calibration tone (1kHz):…* was selected and

the outputs of the sound level meter and the oscilloscope were observed. The calibration tone will play for several seconds and then turn off. After observing the output of the oscilloscope to the calibration tone, an acceptable voltage range (e.g. <u>+</u> 2 dB) was specified in the systemInfo.mat file using the variable system.calStr, which is displayed when selecting to play the calibration tone (in the example, this range is between 42.66-67.60 mV). <u>If the output of the sound level meter does not match *system.caldB*, the system output must be increase using a preamplifier from the system's sound card, or via an external preamplifer</u>. The figures below show the calibration set up, the response of the sound level meter (100 dB SPL) and oscilloscope to the calibration tone (55.7 mV), and the display of system.calStr on the *home window*.

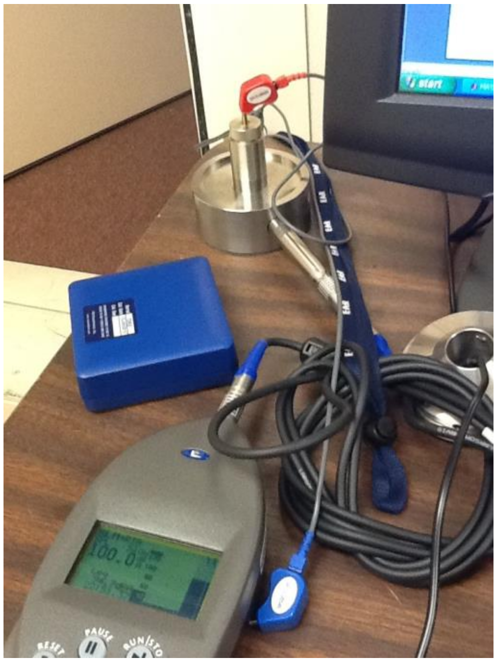

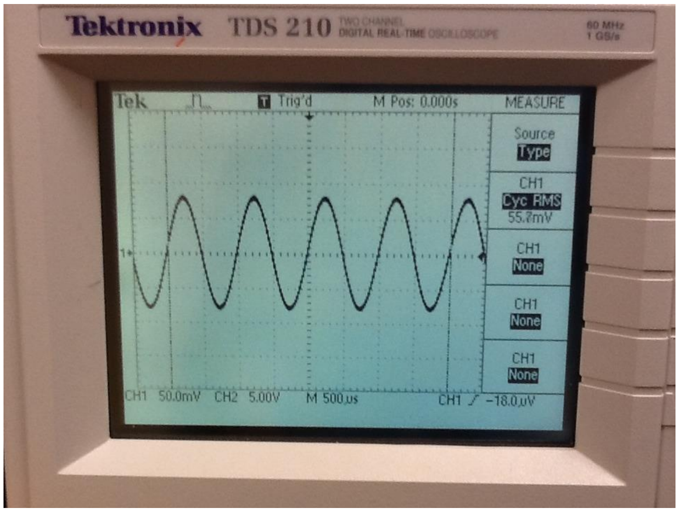

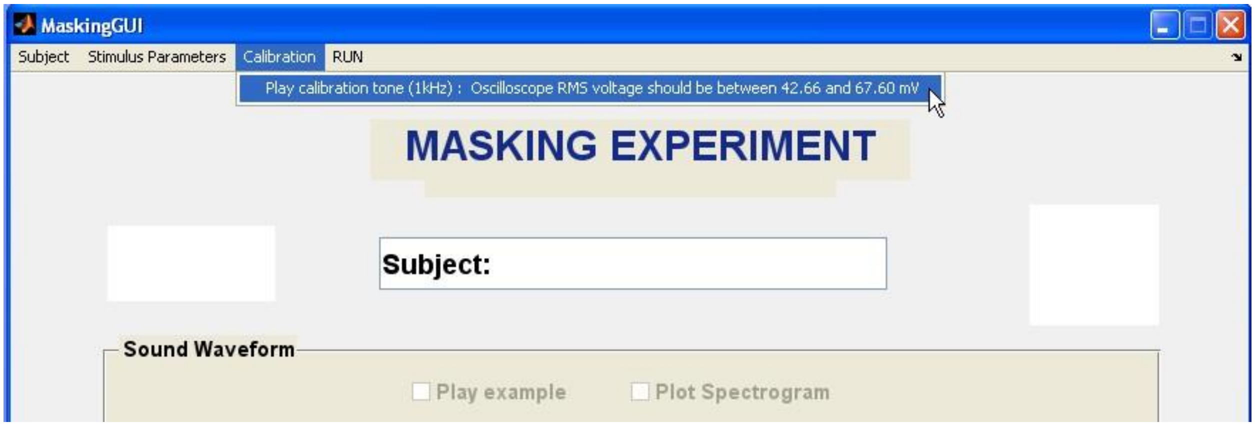

Measuring a Threshold with PsyAcoustX

After selecting the task to measure from PsyAcoustX's opening window (e.g., gap detection), behavioral thresholds can be measured by manually defining a condition, or loading a previously stored condition.

If a masking task is selected threshold collection can be automated using the "enroll subject" feature (see *Enroll Subject* section). If manually defining a condition, or loading a previously stored condition, it is best practice to first assign an identification number (subject ID) to the subject (e.g., "S1"). This will ensure that a unique results file is produced when saving threshold run information. To assign the subject ID, select the following from the *home window*:

Subject > New subject

In window that appears, fill in the subject ID edit box, and then click *Done.*

Manually defining single or multiple experimental conditions

To manually define a condition, perform the following steps

1) With the *home window* open, select to define stimulus parameters from the menu: *Stimulus Parameters > Define*

2) In the window that appears (*Stimulus generation window*), modify parameters as desired by typing values in the boxes. Turning on/off flags activates/deactivates certain modules (e.g., precursor, notched noise).

3) After the stimulus parameters are defined, you can save this set as a stimulus block. Click the "+" in the *Blocks* section of the window to store the block; click "-" to remove a block.

4) To repeat the same stimulus block press the + button twice

5) To add one or more unique additional stimulus blocks, modify the parameters as desired, then press the + button.

Note: If you don't store a blocks the GUI will use what is currently in the window. However, to ensure proper functionality, it is best practice to store at least a single block (i.e., the # of stored blocks should read at least 1).

Note: To measure an unmasked (quiet) signal threshold, select "Measure Target Alone" checkbox.

6) Click the *CONFIRM* button.

7) The GUI then plots the stimulus waveform in the main window. (*Note: this is just a schematic and does not reflect the actual levels of the stimulus*). You can plot its spectrogram (*Plot Spectrogram*) or play the example (*Play example*) by clicking the appropriate checkboxes.

Note: for multiple saved blocks, only the final stimulus block is plotted

8) Run the experiment: *RUN > GO!*

9) In the window that appears (*response window*), click *"START"* to begin running the first block.

10) Once threshold is reached, PsyAcoustX will pause. You can then plot the subject's response track. The threshold (ϴ) and standard deviation (σ) are also printed to the command line.

11) Hit the "*START*" button to begin the next block. When all blocks are complete, a "*Close*" option will appear in the *response window*.

Saving an experimental condition for future data collection

A single experimental condition can be save for future data collection. First enter the stimulus parameters following the steps for manually defining condition (see section above). After the condition is defined, select *File > Save.* A window will appear whereby the location a file name can be specified. By default, the file will be save in the \Data\ folder, unless the user navigates to another directory.

Note: unlike the results file, parameter files can be overwritten!

Loading a single experimental condition

To run a file that has been previously saved using the *File > Save* command, choose the *File > Load* command from the *stimulus generation window*. A standard navigation window will appear to allow the condition file to be loaded .

Note: when running a masking experiment, more options are available when loading experimental conditions. Users can choose to continue with an automated experiment, run a "warm-up" condition, or run a single user-defined condition.

Displaying and saving results

The threshold, standard deviation, and details about the adaptive track are displayed in several ways. First, as the run is being completed, a history of the adaptive track is shown in the *Run tracker window*, and by text printed to the MATLAB command line window. These two windows will also display the threshold and standard deviation of the run when the adaptive track is completed. Additionally, upon completing a run, the *response window* will enable the "plot" button. Pressing this button will result in a graphic representation of the adaptive track, its turn arounds, and the final threshold and standard deviation.

A results file in a .txt format is generated after the subject completes a run. This file is saved in the \Data\ folder and displays information about the subject, the stimulus conditions, the adaptive track, and the measured threshold/standard deviation. By default the results file will be appended to a *subject.txt* file, unless a subject ID is specified when defining the experimental conditions. If the subject ID has been defined, a new .txt file will be saved to [*subjectName_date.txt*]. All additional thresholds collected that day for the subject will be appended to this file; nothing is overwritten.

Playing an Example:

Prior to the start of a run, a button appears at the bottom of the *response window* that allows the subject to hear an example of the condition to be run. To start the example, the user pushes this button

and a “dummy” *response window* appears in which the user then presses the button labeled “START EXAMPLE.”

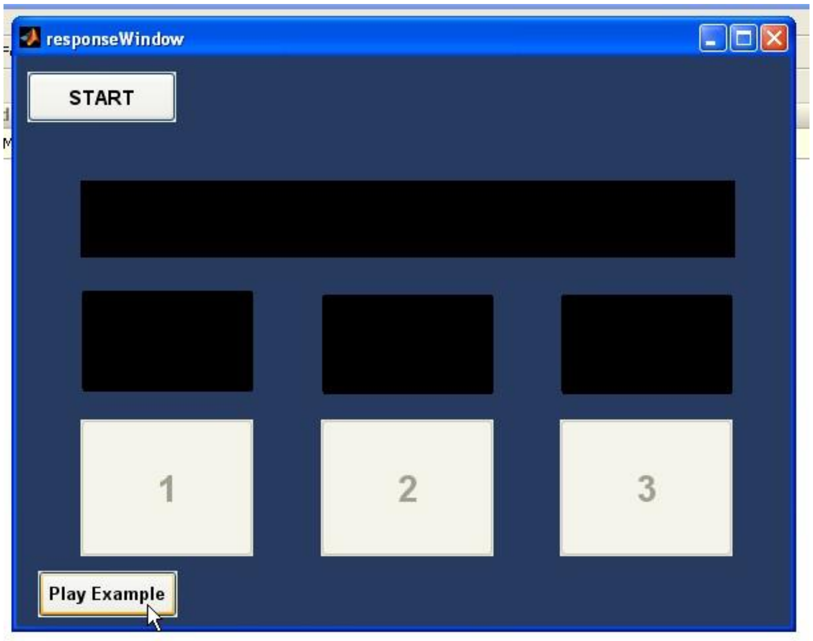

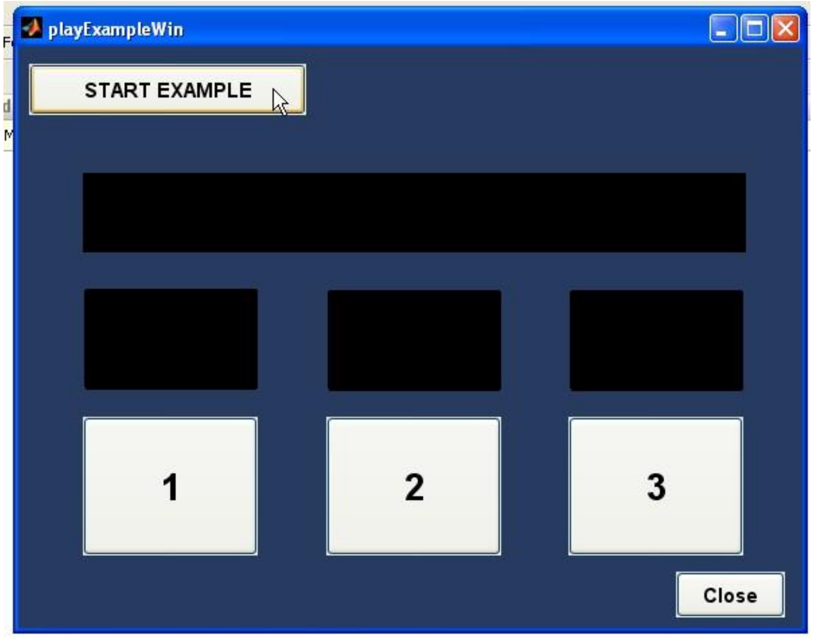

An example then starts where a dummy trial is repeated until the user touches or clicks on the same button used to start the example. For a given repetition of the dummy trial, the program indicates which interval contained the target. After the subject stops the example, buttons are displayed to make the task easier or harder. The subject can click these buttons and restart the example or click close.

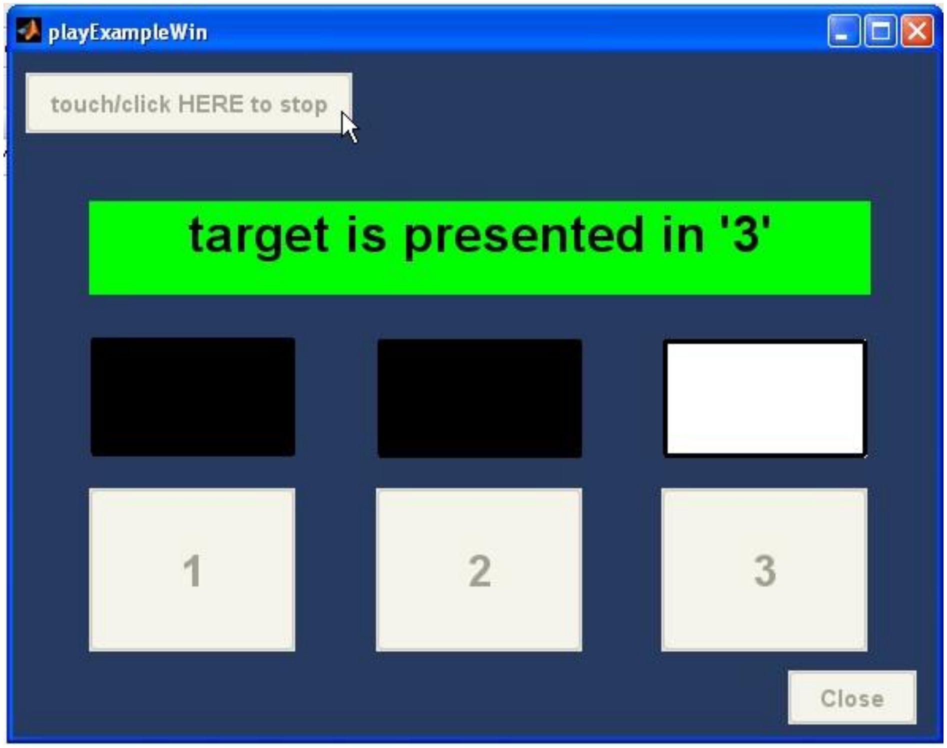
playExampleWin
touch/click HERE to stop
target is presented in '3'
1
2
3
Close

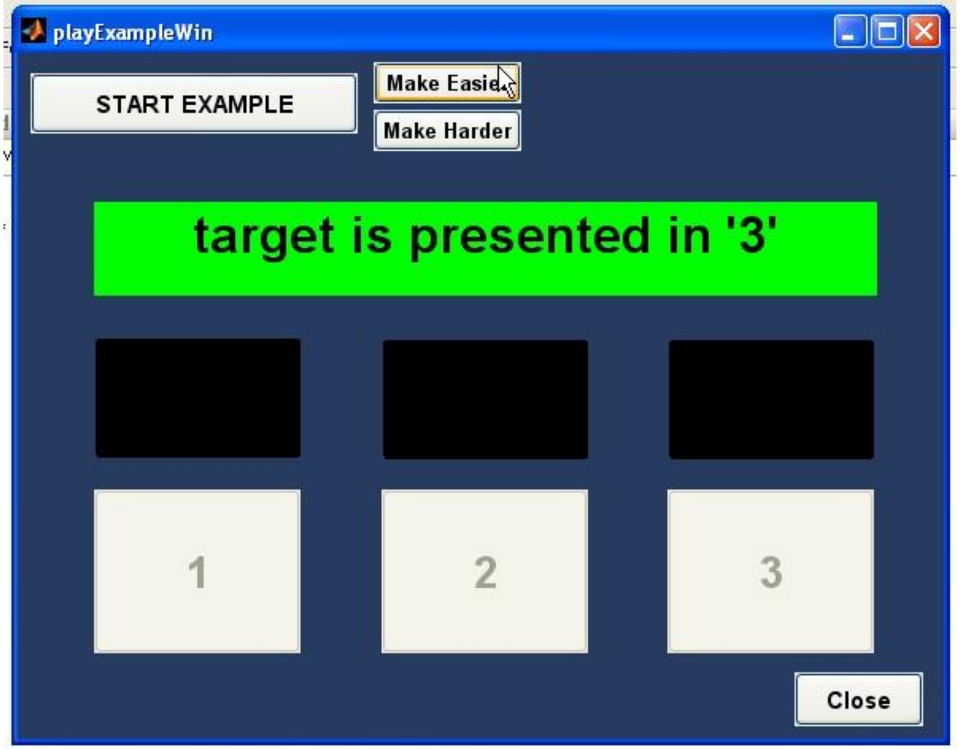
playExampleWin
START EXAMPLE
Make Easier
Make Harder
target is presented in '3'
1
2
3
Close

runTracker:

The *Run tracker window* is displayed while a subject is completing a run. Information regarding the subject, stimuli, current response and current value of the dependent variable are provided. In addition a history of responses and dependent variable values are shown. At the end of a run, the threshold and standard deviation values are displayed in the box "Threshold/STD."

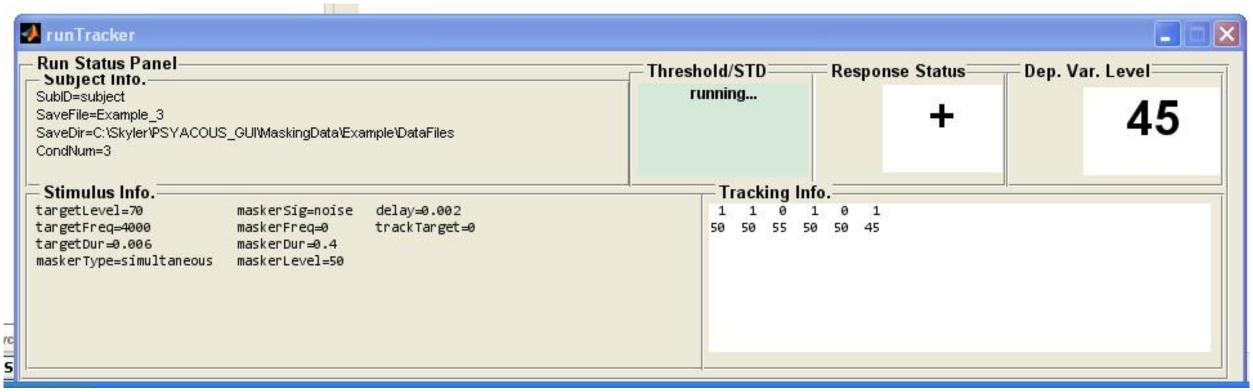

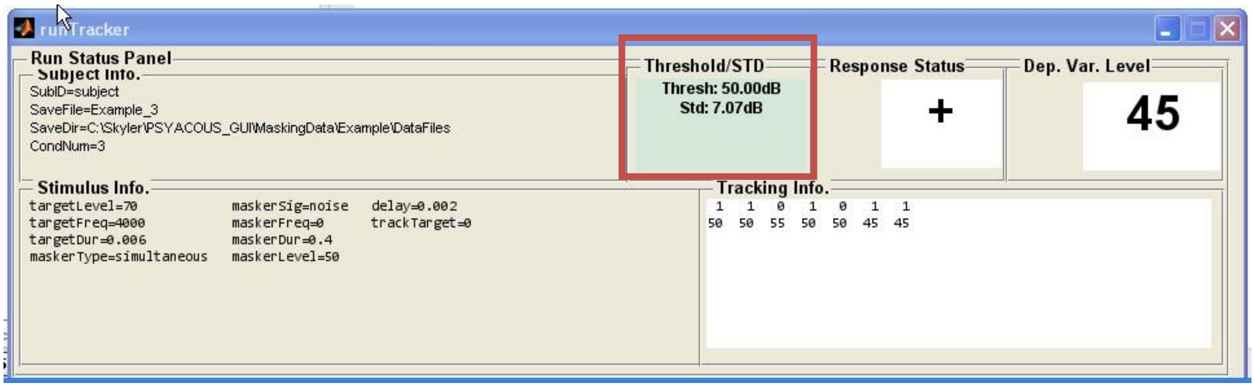

Enrolling a new subject in an experiment (masking tasks only):

To enroll a new subject in an experiment, select **Subject → New Subject** from the menu in the masking *home window*. This will open the *enroll subject window* (i.e., "registerSubject").

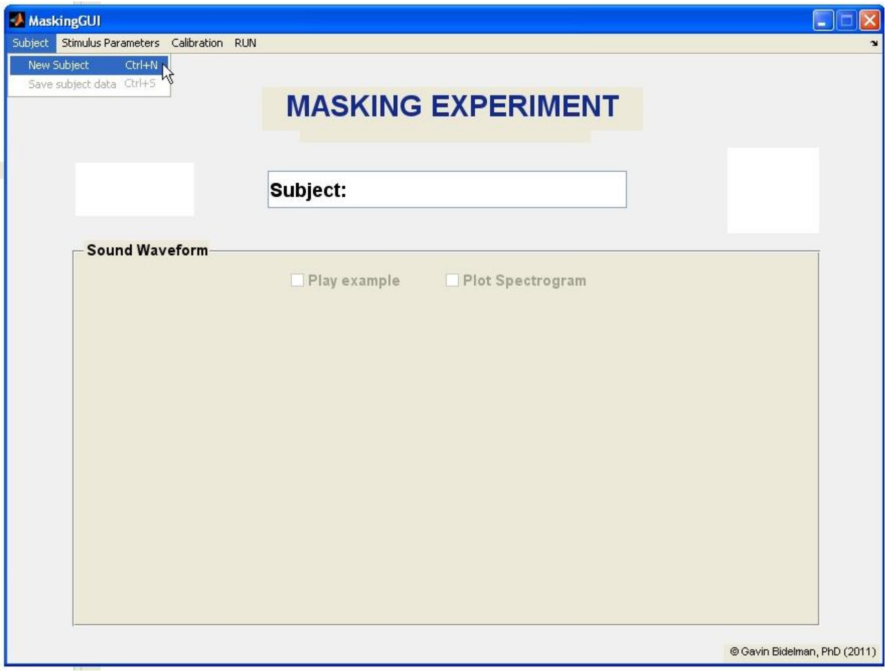

In the *enroll subject window*, the subject's ID and information about the experimental stimuli is defined. The fields associated with stimulus parameters can receive several values if separated by commas.

Example experiment using the enroll subject feature

In the example below, a simultaneous masking experiment involving three signal levels and two delays from masker onset is defined.

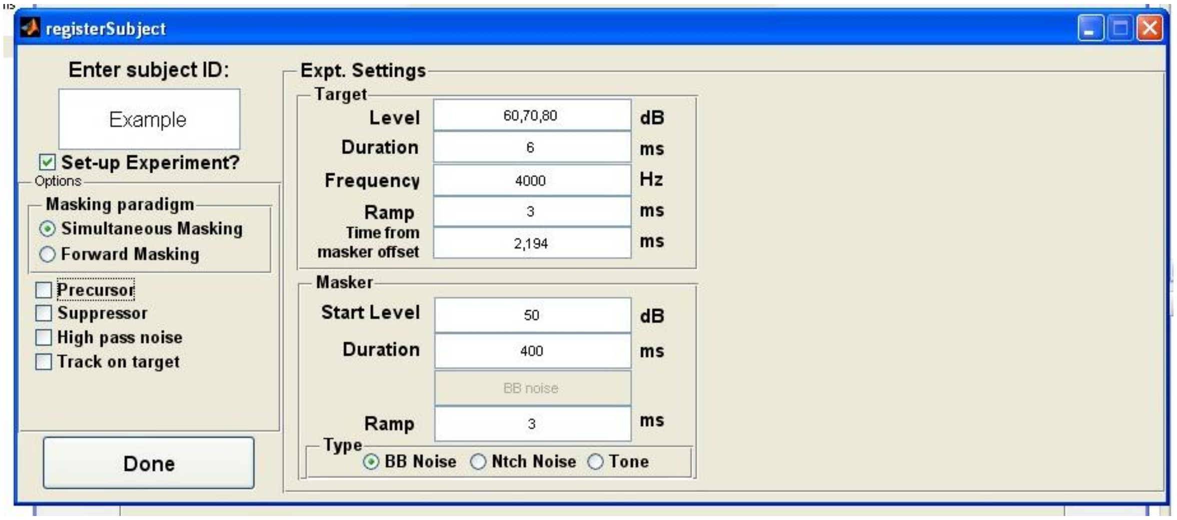

After defining the stimulus parameters in the *enroll subject window*, the user presses the "Done" button to proceed and a table is presented that displays all of the conditions in the experiment. The table shows the stimulus parameters as rows and condition numbers as columns. This example shows the output of the previous simultaneous masking example with three signal levels and two delays from

masker onset. Notice that these signal levels and delays are combined in all possible combinations resulting in six total stimulus conditions. A given combination of signal level and delay from masker onset can be seen by observing the values for the rows labeled "delay" and "targetLevel" for a given column. At this point, the user can review the table to determine if the experiment has been set-up correctly and if so, the user then presses the OK button and then closes the window. After the window is closed, the program will return to the masking home *window*.

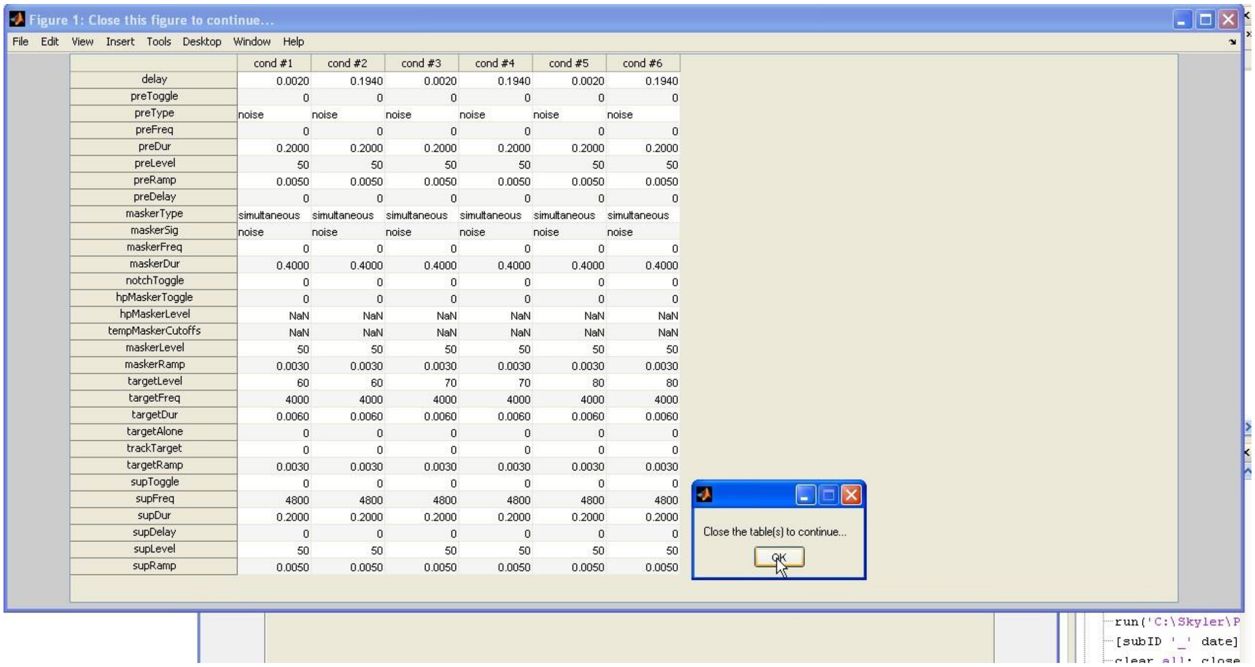

	cond #1	cond #2	cond #3	cond #4	cond #5	cond #6
delay	0.0020	0.1940	0.0020	0.1940	0.0020	0.1940
preToggle	0	0	0	0	0	0
preType	noise	noise	noise	noise	noise	noise
preFreq	0	0	0	0	0	0
preDur	0.2000	0.2000	0.2000	0.2000	0.2000	0.2000
preLevel	50	50	50	50	50	50
preRamp	0.0050	0.0050	0.0050	0.0050	0.0050	0.0050
preDelay	0	0	0	0	0	0
maskerType	simultaneous	simultaneous	simultaneous	simultaneous	simultaneous	simultaneous
maskerSig	noise	noise	noise	noise	noise	noise
maskerFreq	0	0	0	0	0	0
maskerDur	0.4000	0.4000	0.4000	0.4000	0.4000	0.4000
notchToggle	0	0	0	0	0	0
hpMaskerToggle	0	0	0	0	0	0
hpMaskerLevel	NaN	NaN	NaN	NaN	NaN	NaN
tempMaskerCutoffs	NaN	NaN	NaN	NaN	NaN	NaN
maskerLevel	50	50	50	50	50	50
maskerRamp	0.0030	0.0030	0.0030	0.0030	0.0030	0.0030
targetLevel	60	60	70	70	80	80
targetFreq	4000	4000	4000	4000	4000	4000
targetDur	0.0060	0.0060	0.0060	0.0060	0.0060	0.0060
targetAlone	0	0	0	0	0	0
trackTarget	0	0	0	0	0	0
targetRamp	0.0030	0.0030	0.0030	0.0030	0.0030	0.0030
supToggle	0	0	0	0	0	0
supFreq	4800	4800	4800	4800	4800	4800
supDur	0.2000	0.2000	0.2000	0.2000	0.2000	0.2000
supDelay	0	0	0	0	0	0
supLevel	50	50	50	50	50	50
supRamp	0.0050	0.0050	0.0050	0.0050	0.0050	0.0050

Measuring thresholds in quiet at multiple frequencies

The Register Subject dialog can be used to create stimulus conditions similar to those used in measuring a puretone audiogram. To measure thresholds in quiet, enter the subject's ID and then set the masking paradigm to Simultaneous Masking and click the box labeled "Track on target." In the panel labeled "target" set the *Time from masker offset* field to zero and then enter the desired target *Duration* (a scalar), *Ramp* (a scalar) and test *Frequency* (a vector with each value separated by a comma). In the panel labeled "masker," set the masker duration to be greater than the target duration (e.g. if you target is 350 ms, set your masker to 400 ms) and set the masker level to -120 dB. Finally, in the target panel, set the *InitLvl SNR* to result in a 20-30 dB SPL target level. For a masker that is -120 dB this initLvl SNR will be 140-150 dB. A screenshot of an experiment using audiometric frequencies and a target duration of 350 ms is provided below. The same measurement is also shown for a shorter signal duration (6 ms).

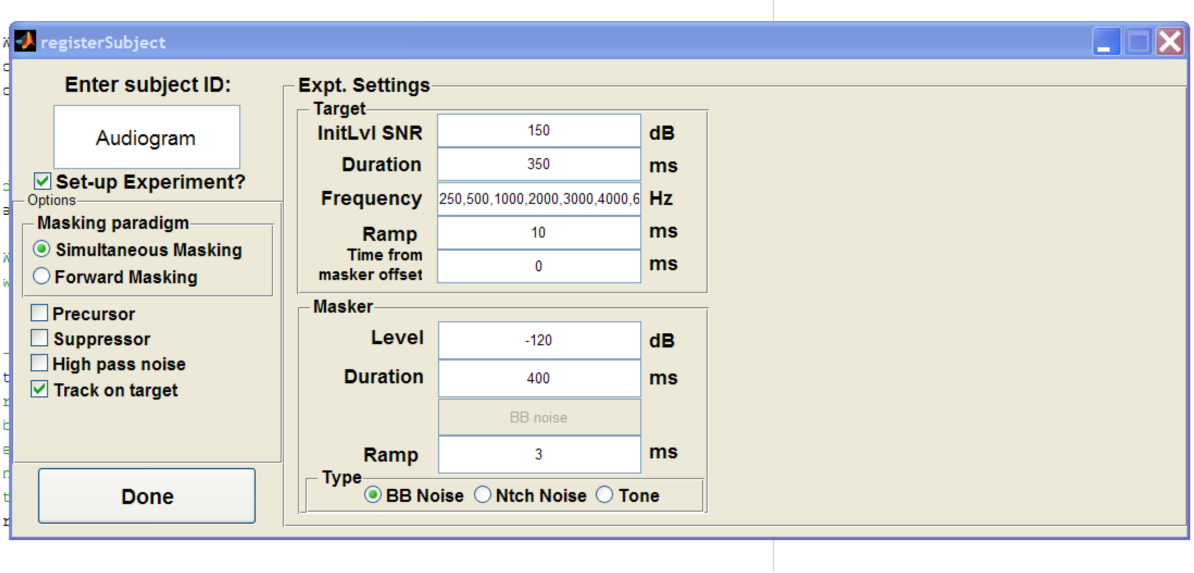

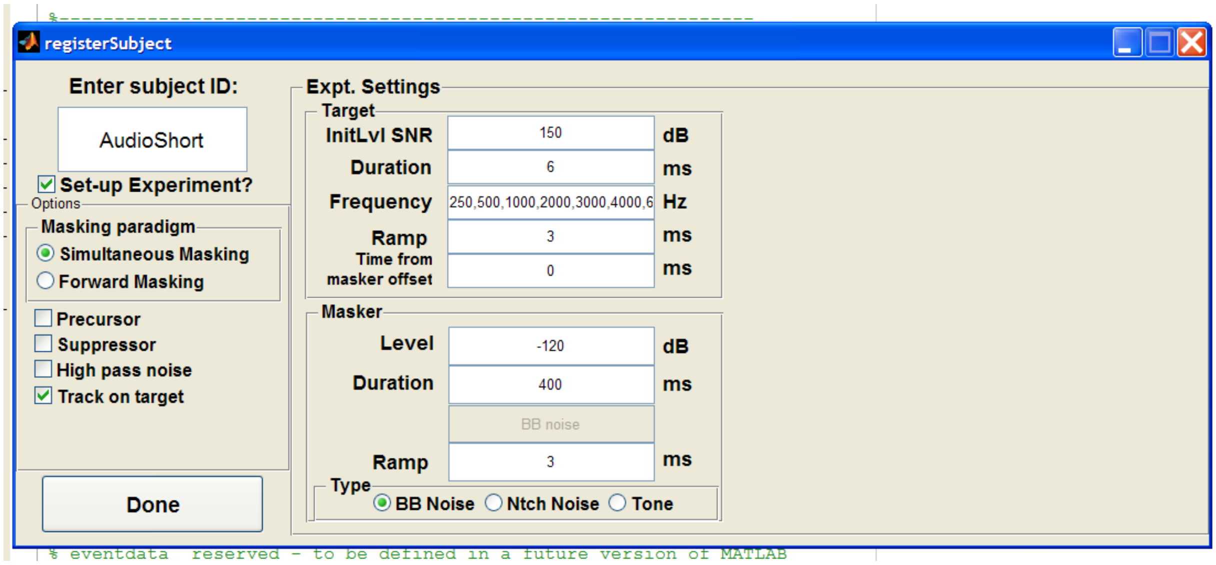

Running an experiment on an enrolled subject

To run an experiment on an enrolled subject, select **Stimulus Parameters → Define** on the menu in the masking *home window*. This will open the *stimulus generation window (i.e.,* “selectStimParams”).

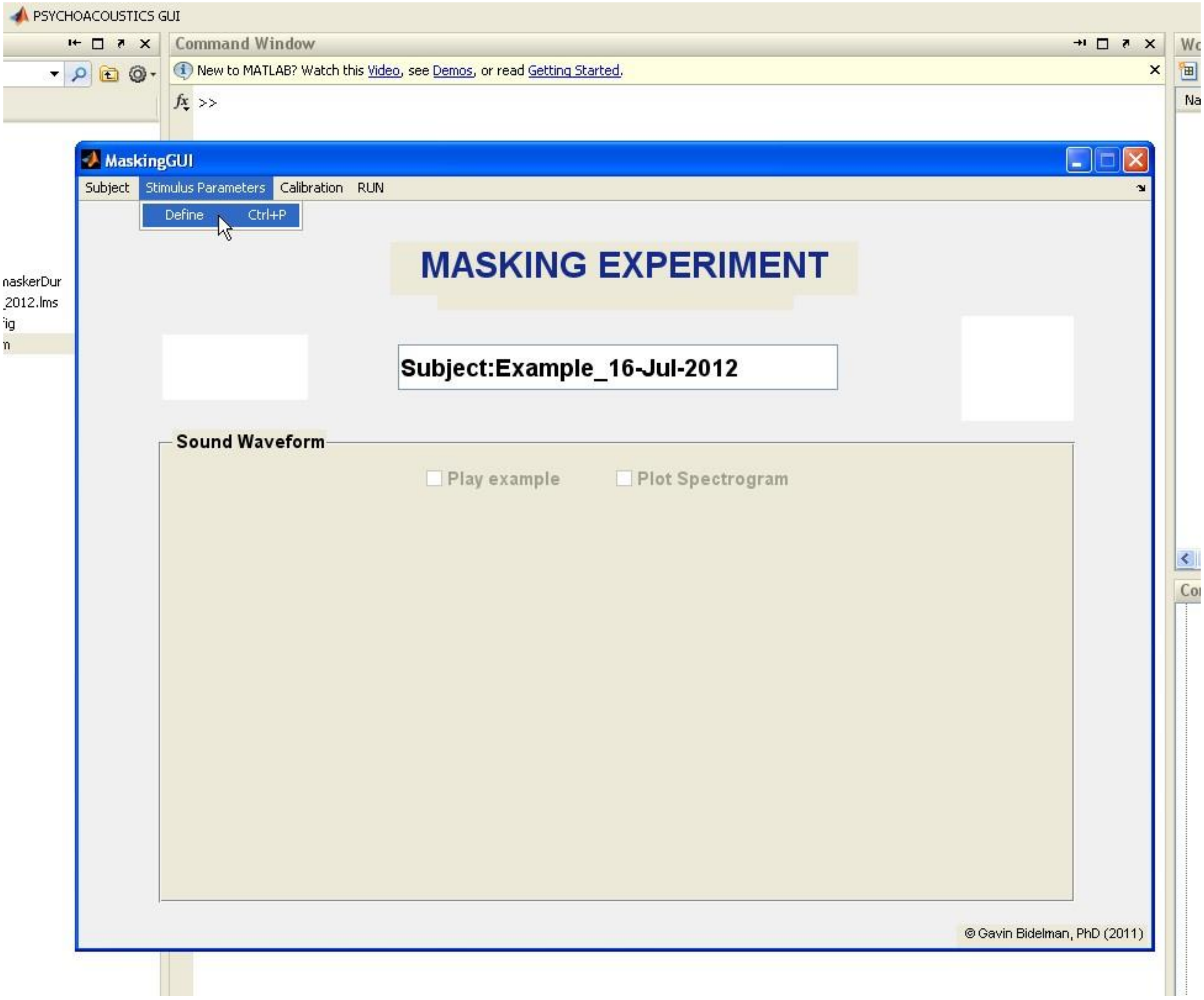
PSYCHOACOUSTICS GUI
Command Window
New to MATLAB? Watch this Video, see Demos, or read Getting Started.
MaskingGUI
Subject
Stimulus Parameters
Calibration
RUN
Define
Ctrl+P
MASKING EXPERIMENT
Subject:Example_16-Jul-2012
Sound Waveform
Play example
Plot Spectrogram
© Gavin Bidelman, PhD (2011)

The user then selects **File → Load** from the menu in the *stimulus generation window* and is prompted to select one of the following options: 1) Run next condition in selected experiment, or 2) Run a “warm-up” condition, or 3) Run a single user-defined condition.

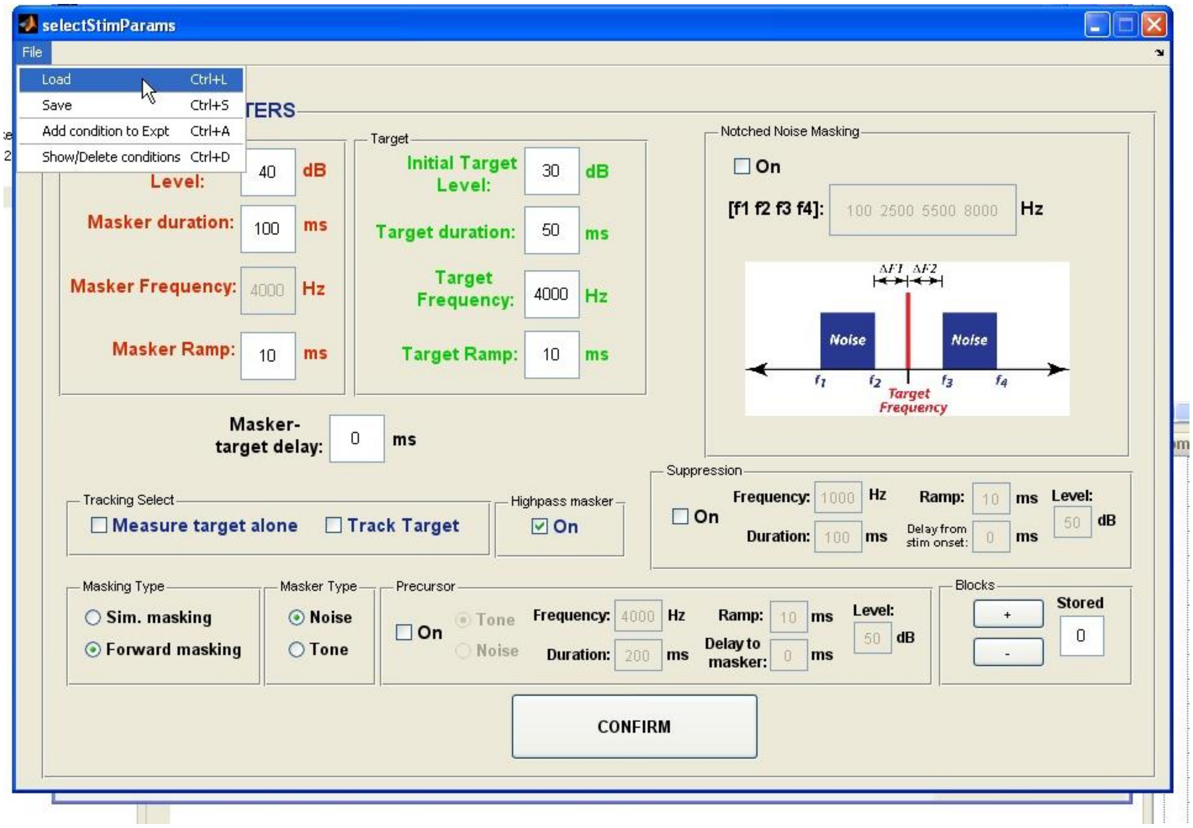

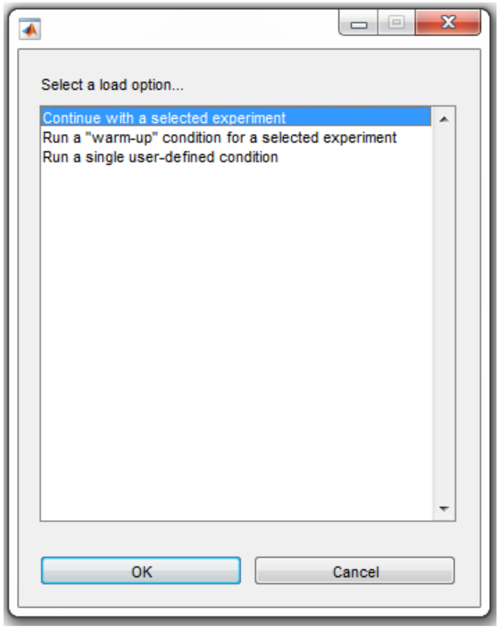

The user then selects option #1 and is prompted to locate the subjects folder and “*<name>*ExptInfo.mat” file that was created when the experiment was define using the *enroll subject window*.

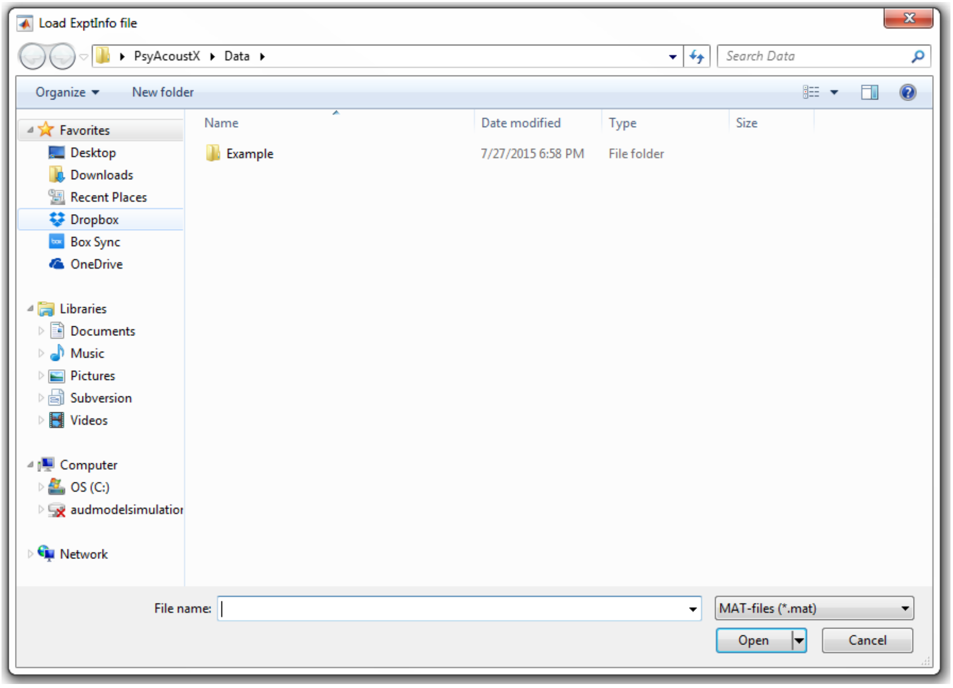

The subjects folder is located in the Data directory and has the same name as the subject ID (default is "subject") defined when the experiment was created using the *enroll subject window*.

The ExptInfo.mat file is located within the subject's directory, with the subject's ID as a prefix to the file name. After the ExptInfo.mat file is located, click "open" in the prompt window. The program will then automatically load the next condition in the experiment and display the time waveform of the stimuli in the masking *home window*.

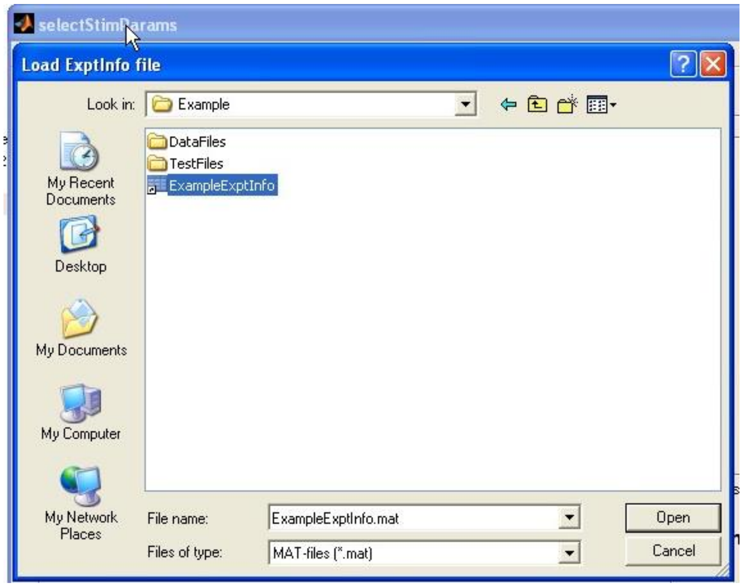

To start a run, selected **RUN → GO!** from the menu in masking *home window*. This will open the window that visually displays the observation intervals while the stimuli are played and the collects the subject's responses (i.e., the *response window*).

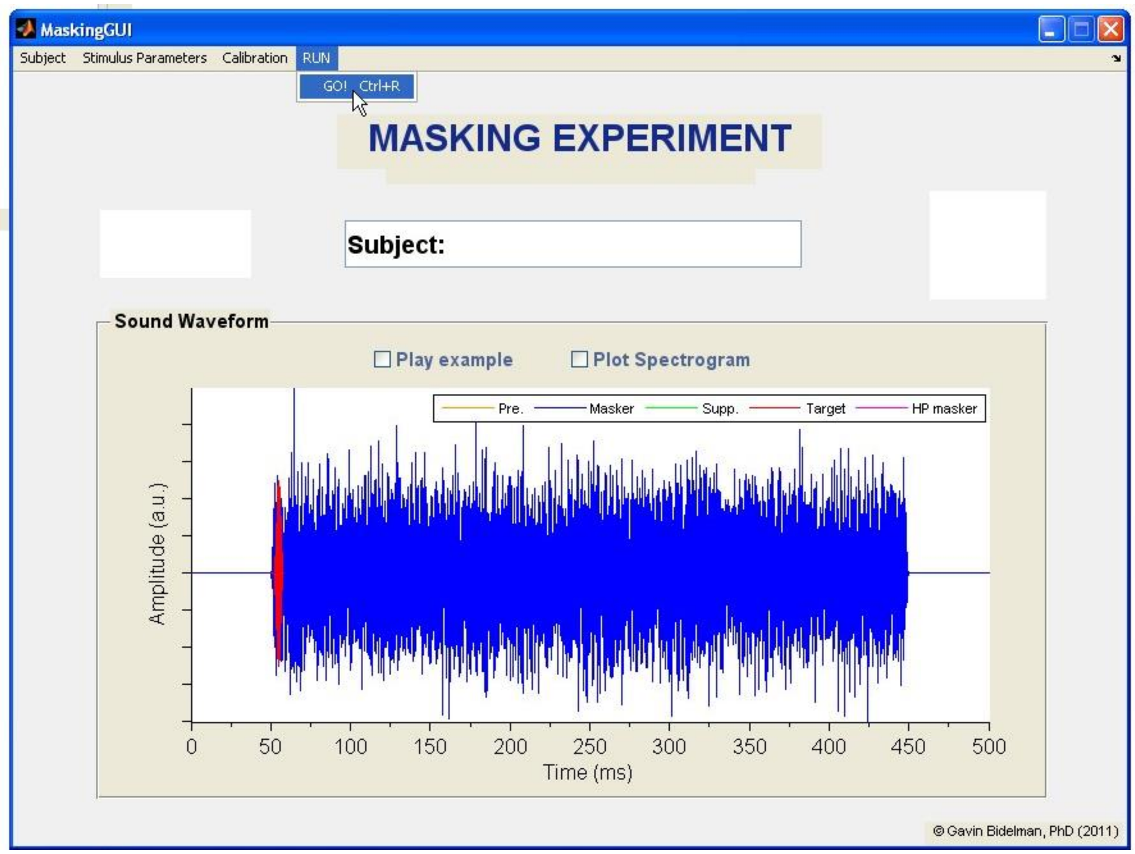

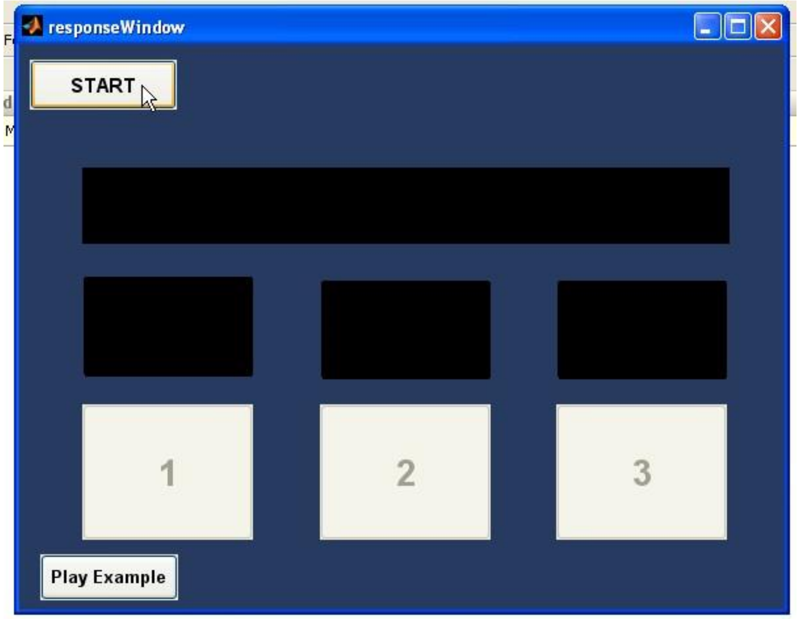

The subject then presses the START button on the *response window* to begin a run.

Information about the current condition is presented in the *Run Tracker window*.

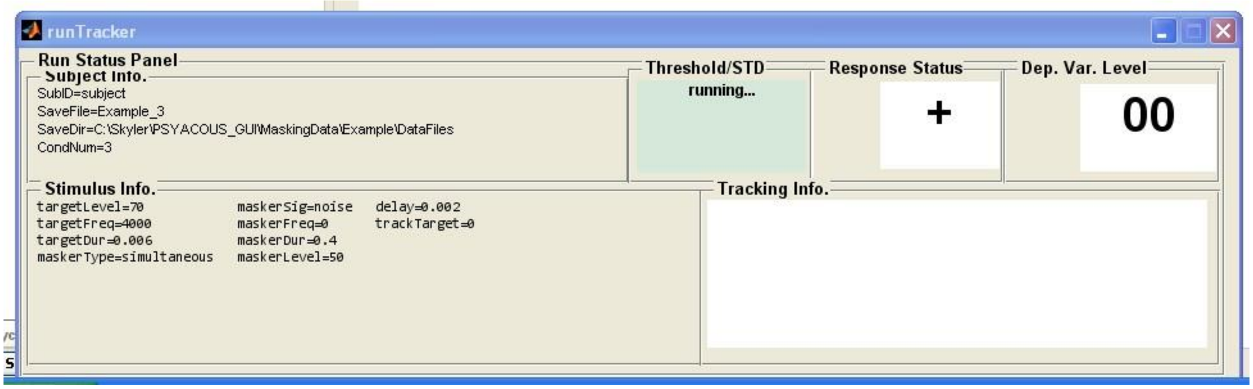

After the START button is pressed, the adaptive three-interval forced-choice procedure will begin where the subject will listen and respond to several trials before a threshold is obtained. During a run, the *response window* will display the word “running” in the upper left corner.

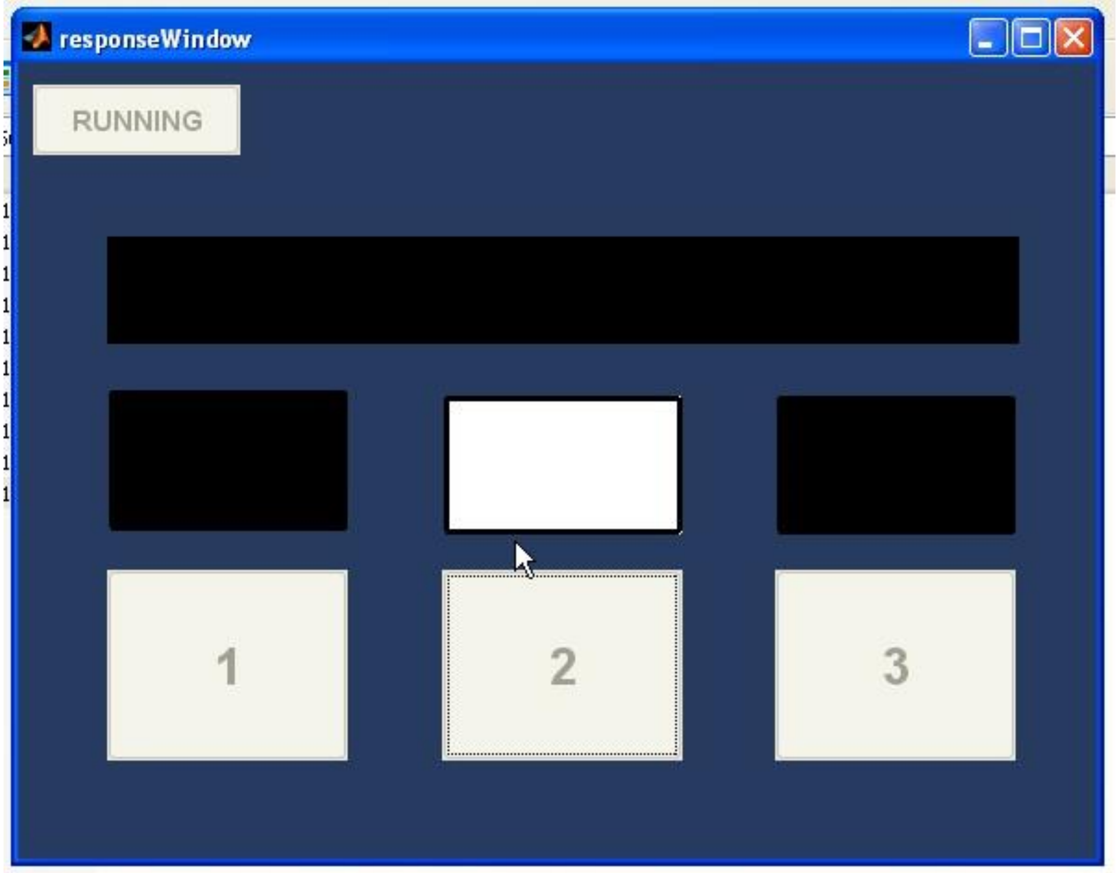

After a run is finished, the START button will reappear in blue, prompting the user to complete the next run of the condition (if multiple repetitions were selected). When all the runs of a condition are completed, the START button will be deactivated and a button labeled “NEXT” will appear in the bottom right-hand side of the window. When the subject presses this button, the next condition of the

experiment will be loaded automatically. Subjects should be encouraged to take breaks only when the START button is enabled. If the START button is deactivated or displays "running," subjects should be encouraged to continue with the task until the START button appears and is enabled. When all conditions are finished, a small window will appear displaying the phrase "You have completed all conditions!" After the subject presses the OK button in this window, the program will save the remaining data and reset.

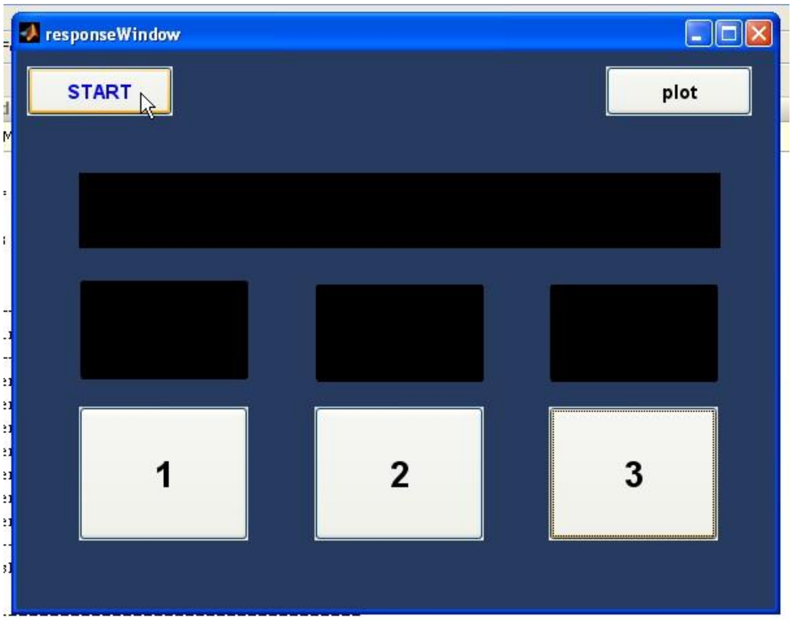

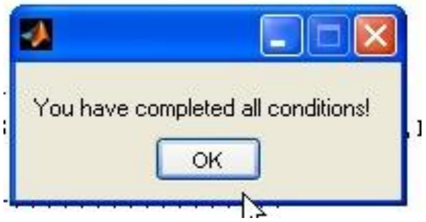

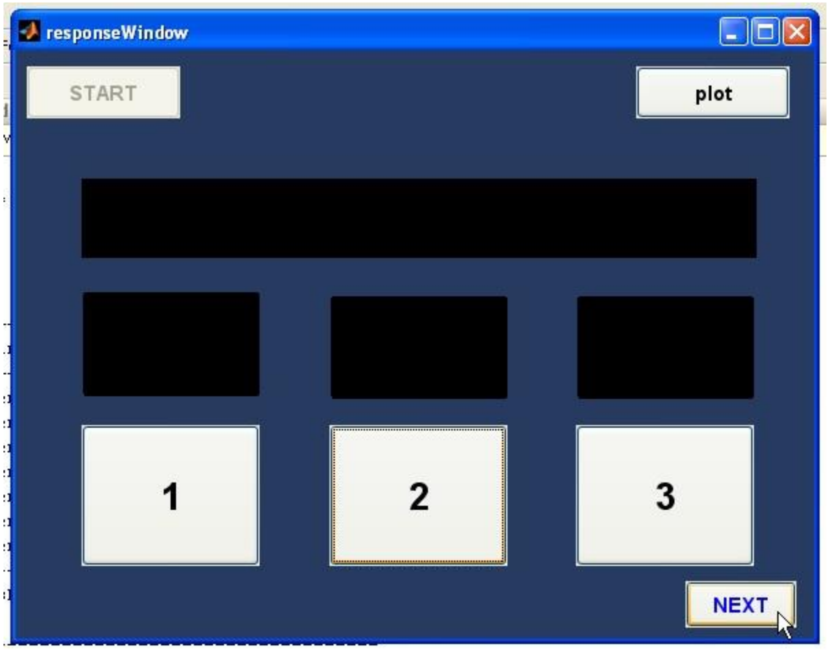

Removing conditions from an experiment:

To remove conditions from an experiment, **select File → Show/Delete conditions** in the menu of the *stimulus generation window*.

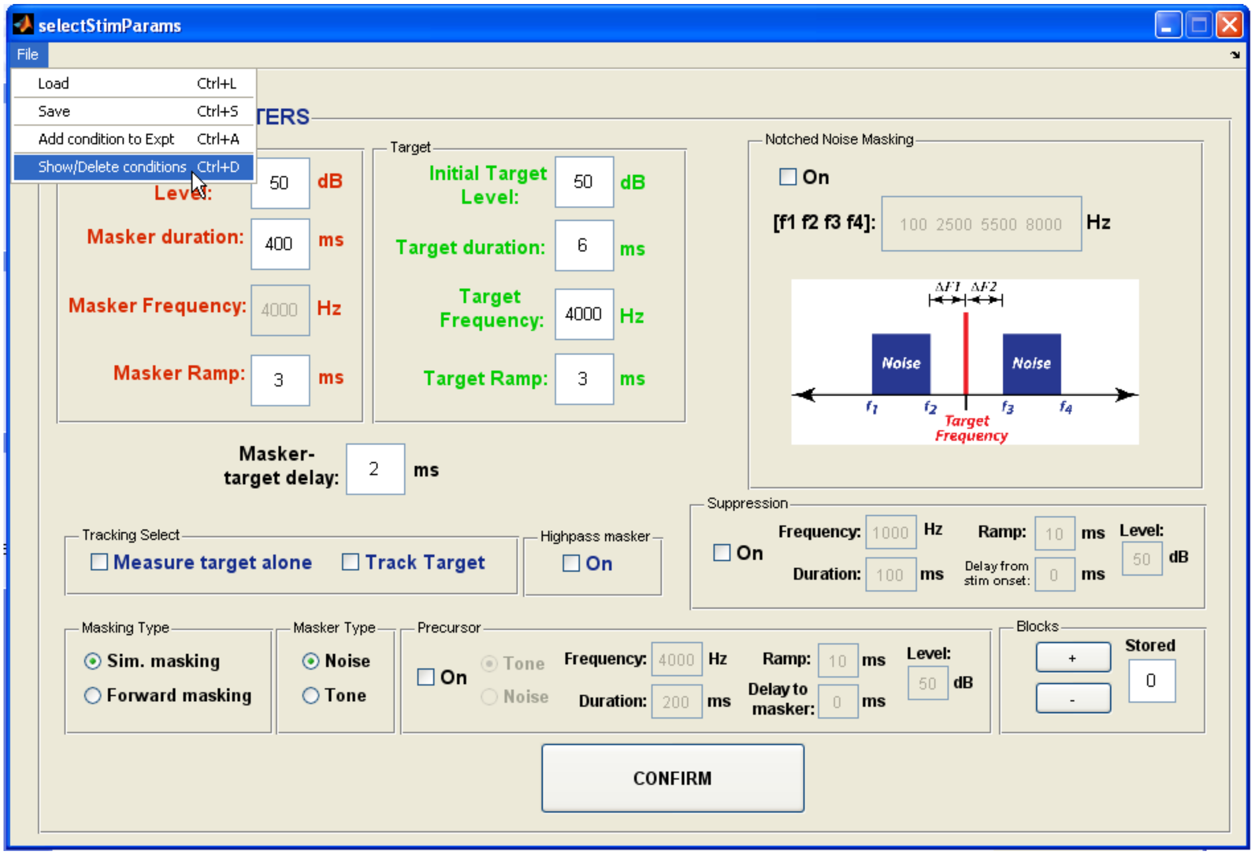

The user is then prompted to locate the subject's directory where the condition is to be removed. Once the directory is located, the user then selects the ExptInfo.mat file and a table showing the current list is displayed.

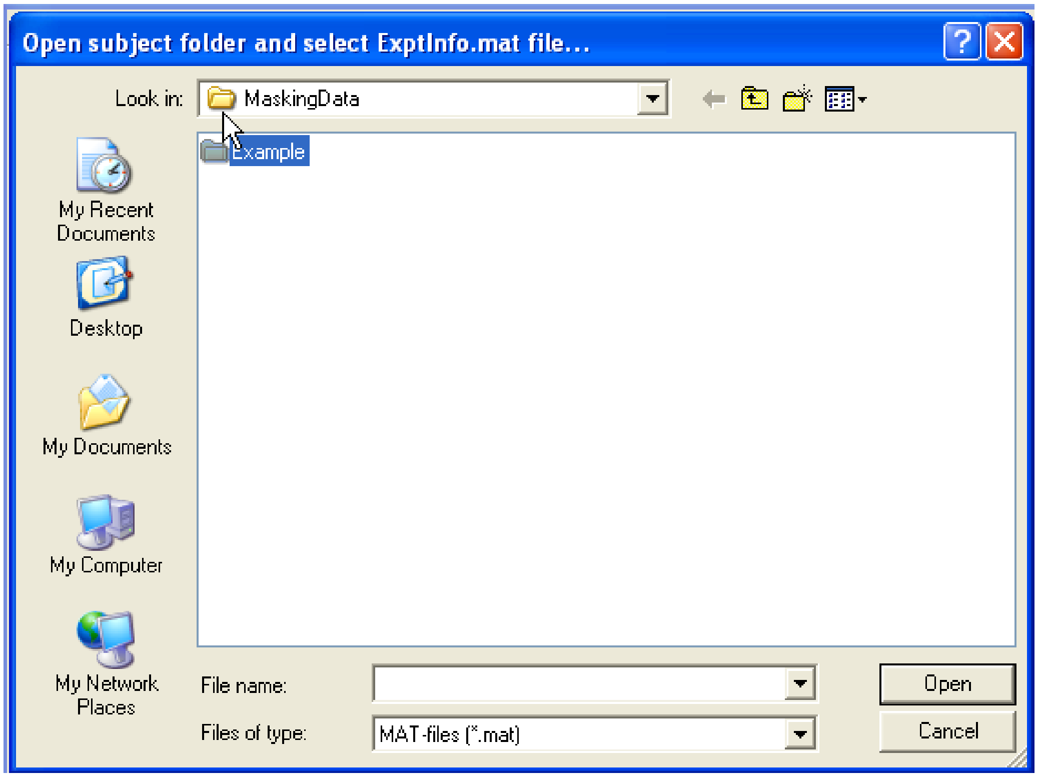

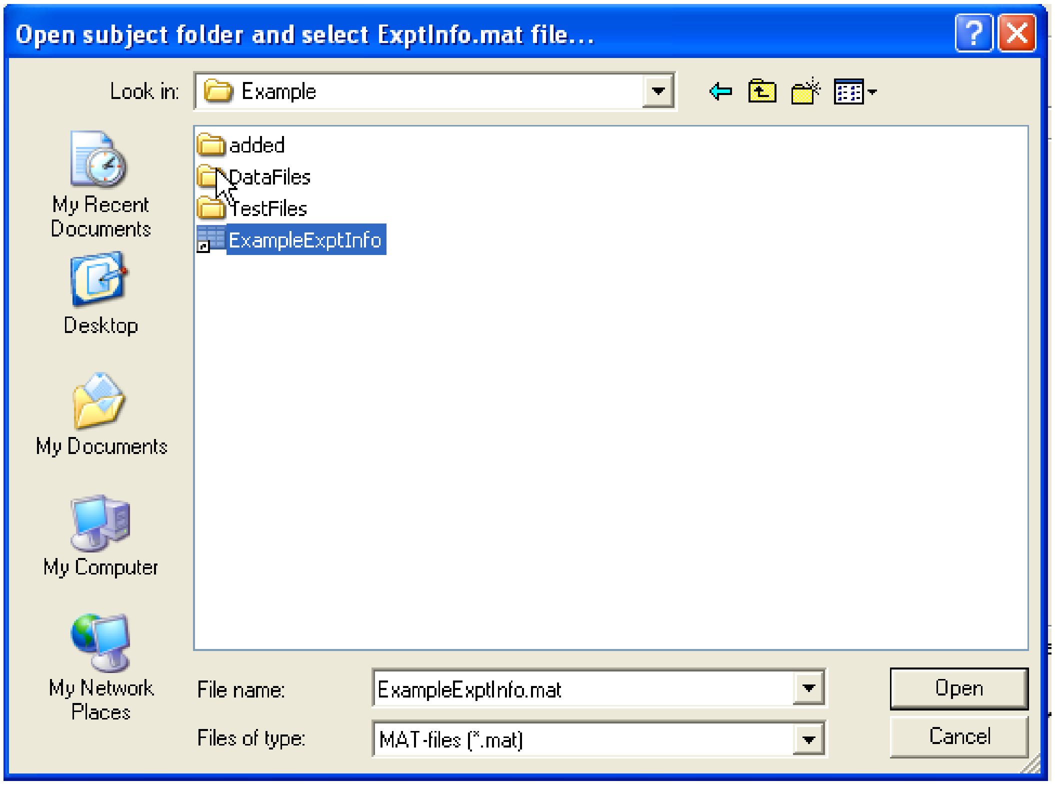

The user is then asked if a condition(s) is to be deleted. If the user selects "No," the table will continue to be displayed until the user closes the window. If the user selects "Yes," a message appears indicating how to delete a condition. Specifically, this message indicates that a condition will be deleted if a cell corresponding to a condition is left-clicked and then right-clicked.

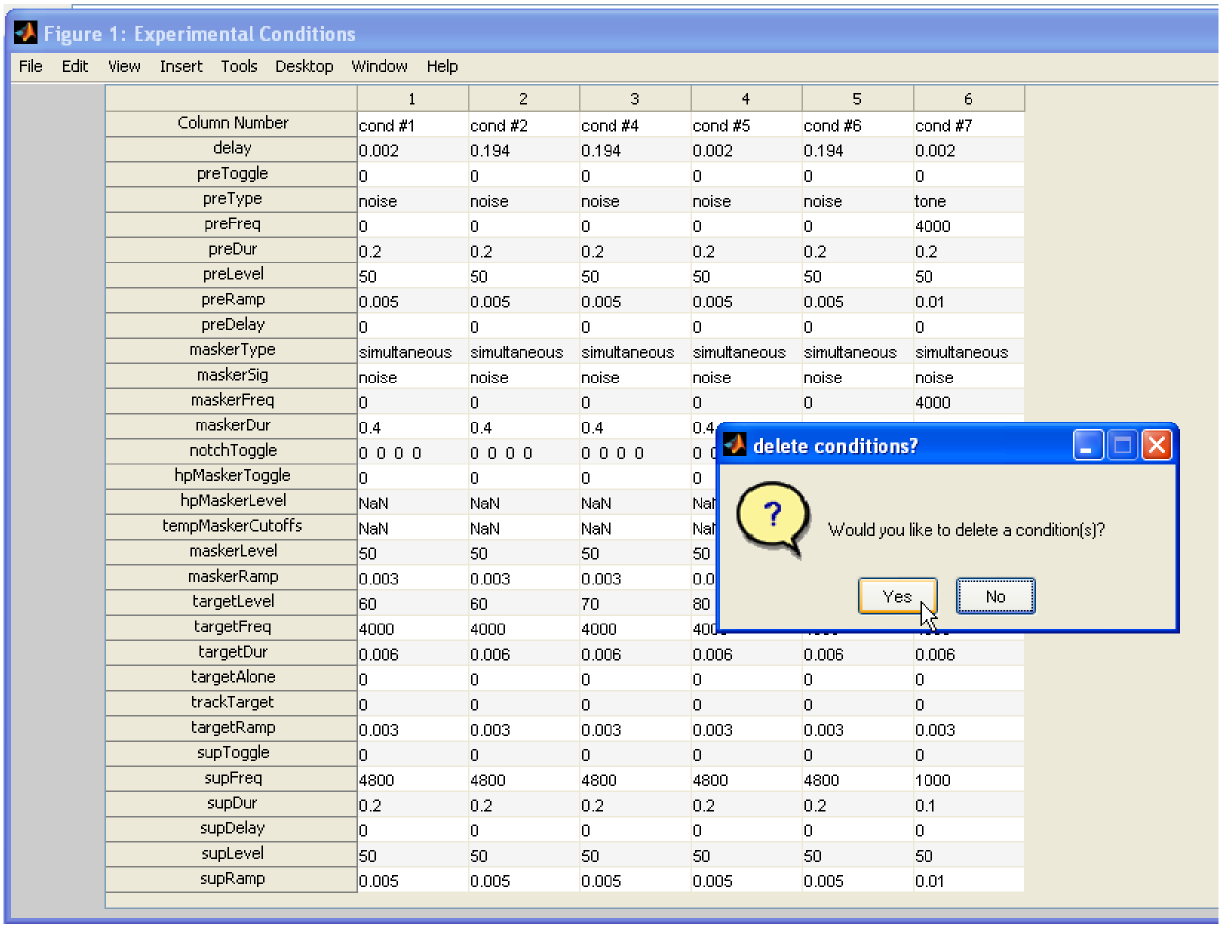

	1	2	3	4	5	6
Column Number	cond #1	cond #2	cond #4	cond #5	cond #6	cond #7
delay	0.002	0.194	0.194	0.002	0.194	0.002
preToggle	0	0	0	0	0	0
preType	noise	noise	noise	noise	noise	tone
preFreq	0	0	0	0	0	4000
preDur	0.2	0.2	0.2	0.2	0.2	0.2
preLevel	50	50	50	50	50	50
preRamp	0.005	0.005	0.005	0.005	0.005	0.01
preDelay	0	0	0	0	0	0
maskerType	simultaneous	simultaneous	simultaneous	simultaneous	simultaneous	simultaneous
maskerSig	noise	noise	noise	noise	noise	noise
maskerFreq	0	0	0	0	0	4000
maskerDur	0.4	0.4	0.4	0.4	[illegible]	[illegible]
notchToggle	0 0 0 0	0 0 0 0	0 0 0 0	[illegible]	[illegible]	[illegible]
hpMaskerToggle	0	0	0	0	[illegible]	[illegible]
hpMaskerLevel	NaN	NaN	NaN	[illegible]	[illegible]	[illegible]
tempMaskerCutoffs	NaN	NaN	NaN	[illegible]	[illegible]	[illegible]
maskerLevel	50	50	50	50	[illegible]	[illegible]
maskerRamp	0.003	0.003	0.003	[illegible]	[illegible]	[illegible]
targetLevel	60	60	70	80	[illegible]	[illegible]
targetFreq	4000	4000	4000	[illegible]	[illegible]	[illegible]
targetDur	0.006	0.006	0.006	0.006	0.006	0.006
targetAlone	0	0	0	0	0	0
trackTarget	0	0	0	0	0	0
targetRamp	0.003	0.003	0.003	0.003	0.003	0.003
supToggle	0	0	0	0	0	0
supFreq	4800	4800	4800	4800	4800	1000
supDur	0.2	0.2	0.2	0.2	0.2	0.1
supDelay	0	0	0	0	0	0
supLevel	50	50	50	50	50	50
supRamp	0.005	0.005	0.005	0.005	0.005	0.01

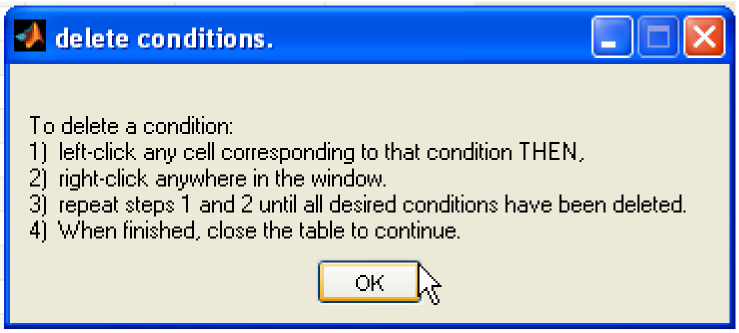

Below is an example of a condition being removed from the experimental list of conditions. Notice that in the first figure condition #5 appears, but in the second it is deleted. The user can continue to delete conditions until all desired conditions have been deleted, after which, the user closes the window.

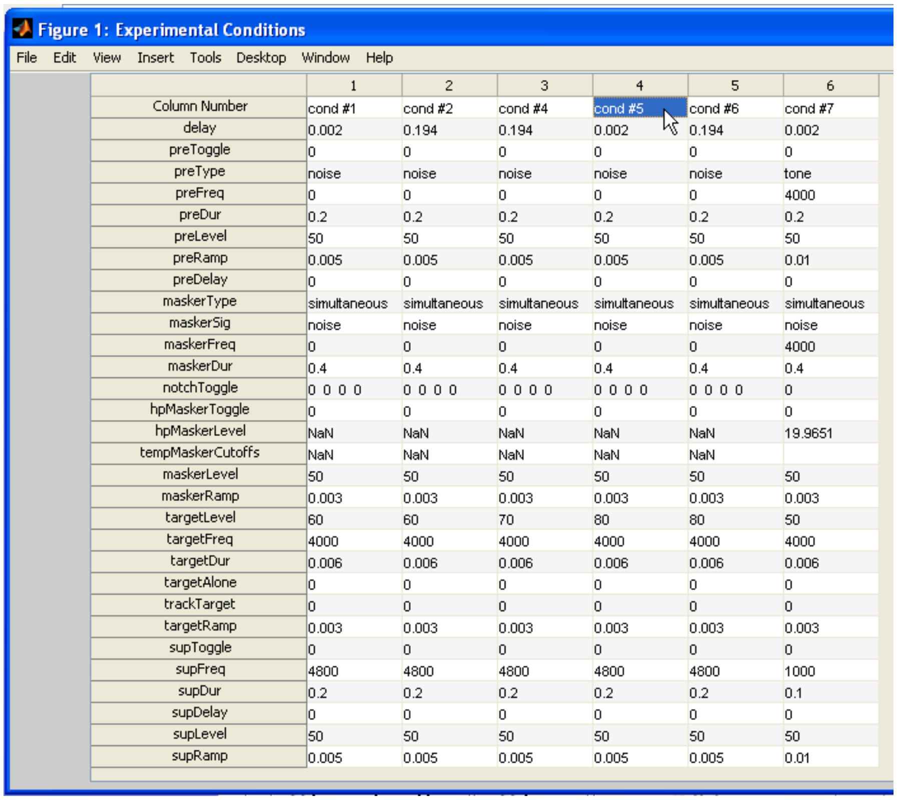

	1	2	3	4	5	6
Column Number	cond #1	cond #2	cond #4	cond #5	cond #6	cond #7
delay	0.002	0.194	0.194	0.002	0.194	0.002
preToggle	0	0	0	0	0	0
preType	noise	noise	noise	noise	noise	tone
preFreq	0	0	0	0	0	4000
preDur	0.2	0.2	0.2	0.2	0.2	0.2
preLevel	50	50	50	50	50	50
preRamp	0.005	0.005	0.005	0.005	0.005	0.01
preDelay	0	0	0	0	0	0
maskerType	simultaneous	simultaneous	simultaneous	simultaneous	simultaneous	simultaneous
maskerSig	noise	noise	noise	noise	noise	noise
maskerFreq	0	0	0	0	0	4000
maskerDur	0.4	0.4	0.4	0.4	0.4	0.4
notchToggle	0 0 0 0	0 0 0 0	0 0 0 0	0 0 0 0	0 0 0 0	0
hpMaskerToggle	0	0	0	0	0	0
hpMaskerLevel	NaN	NaN	NaN	NaN	NaN	19.9651
tempMaskerCutoffs	NaN	NaN	NaN	NaN	NaN	
maskerLevel	50	50	50	50	50	50
maskerRamp	0.003	0.003	0.003	0.003	0.003	0.003
targetLevel	60	60	70	80	80	50
targetFreq	4000	4000	4000	4000	4000	4000
targetDur	0.006	0.006	0.006	0.006	0.006	0.006
targetAlone	0	0	0	0	0	0
trackTarget	0	0	0	0	0	0
targetRamp	0.003	0.003	0.003	0.003	0.003	0.003
supToggle	0	0	0	0	0	0
supFreq	4800	4800	4800	4800	4800	1000
supDur	0.2	0.2	0.2	0.2	0.2	0.1
supDelay	0	0	0	0	0	0
supLevel	50	50	50	50	50	50
supRamp	0.005	0.005	0.005	0.005	0.005	0.01

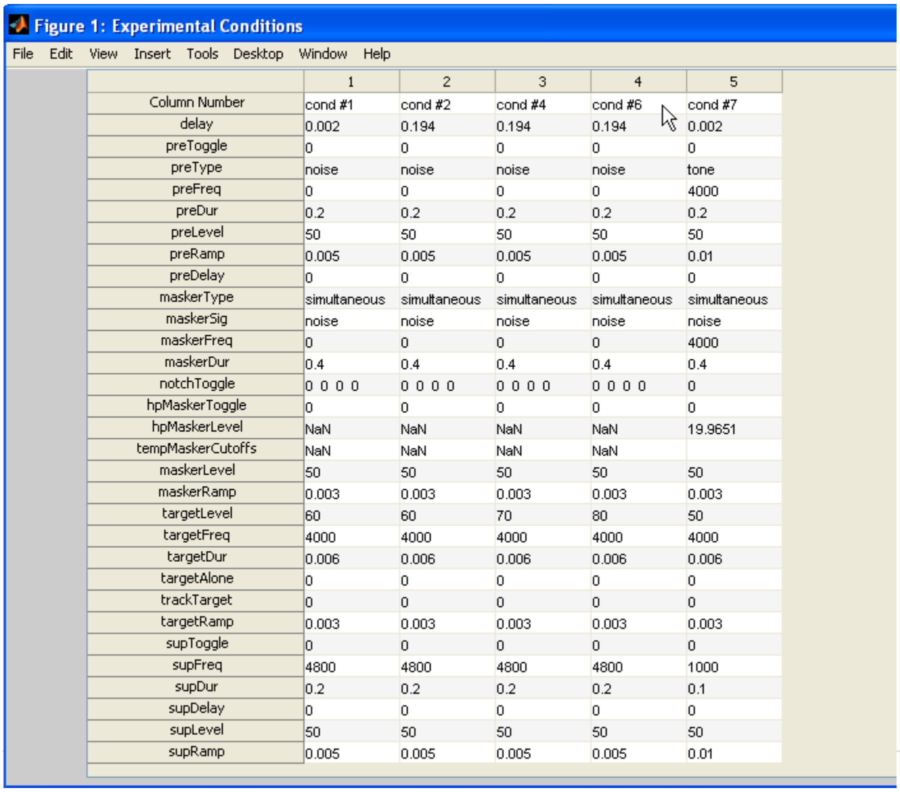

	1	2	3	4	5
Column Number	cond #1	cond #2	cond #4	cond #6	cond #7
delay	0.002	0.194	0.194	0.194	0.002
preToggle	0	0	0	0	0
preType	noise	noise	noise	noise	tone
preFreq	0	0	0	0	4000
preDur	0.2	0.2	0.2	0.2	0.2
preLevel	50	50	50	50	50
preRamp	0.005	0.005	0.005	0.005	0.01
preDelay	0	0	0	0	0
maskerType	simultaneous	simultaneous	simultaneous	simultaneous	simultaneous
maskerSig	noise	noise	noise	noise	noise
maskerFreq	0	0	0	0	4000
maskerDur	0.4	0.4	0.4	0.4	0.4
notchToggle	0 0 0 0	0 0 0 0	0 0 0 0	0 0 0 0	0
hpMaskerToggle	0	0	0	0	0
hpMaskerLevel	NaN	NaN	NaN	NaN	19.9651
tempMaskerCutoffs	NaN	NaN	NaN	NaN	
maskerLevel	50	50	50	50	50
maskerRamp	0.003	0.003	0.003	0.003	0.003
targetLevel	60	60	70	80	50
targetFreq	4000	4000	4000	4000	4000
targetDur	0.006	0.006	0.006	0.006	0.006
targetAlone	0	0	0	0	0
trackTarget	0	0	0	0	0
targetRamp	0.003	0.003	0.003	0.003	0.003
supToggle	0	0	0	0	0
supFreq	4800	4800	4800	4800	1000
supDur	0.2	0.2	0.2	0.2	0.1
supDelay	0	0	0	0	0
supLevel	50	50	50	50	50
supRamp	0.005	0.005	0.005	0.005	0.01

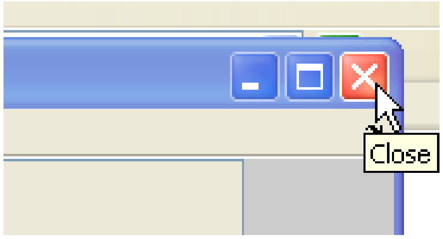

Supplement: Supplementary file 2 [file Data_Sheet_2.PDF]
