# Supplementary material for: PsyAcoustX: A flexible MATLAB® package for psychoacoustics research
Source: Front Psychol. 2015 Oct 12;6:1498. doi: 10.3389/fpsyg.2015.01498 (PMC4601020; doi:10.3389/fpsyg.2015.01498)
Supplement: Supplementary file 1 [file Data_Sheet_1.ZIP › PsyAcoustX/MaskingGUI/README.doc]

***Masking GUI***

**Programmed by: Gavin Bidelman, Ph.D., Purdue University**

**Email: gbidelma@purdue.edu**

**Last Updated: June 28, 2011**

***Setup:***

1) Make sure Masking GUI and its subfolders are in MATLAB’s path: In MATLAB, *File > Set Path > Add with subfolders.*
2) Calibration:
a) First open the Lynx sound card mixer.
b) Set nominal operating level of +4dBu (professional audio line level standard).
c) Set fader on Ch.3/4 to -19 dB attenuation.
d) ER2 headphones were calibrated using the configuration shown in the figure below. The TDT headphone buffer functions as a direct input (DI) box—matching impedance between ER2 headphones and Lynx II XLR output—and also ensures the signal chain is balanced throughout.

e) Run MaskerGUI.m. Select *Calibration > Play calibration tone…* This will play a 10 sec, 1kHz sinusoid with an amplitude of ±1 (RMS: 1/√2 = 0.707).
f) Increase Lynx mixer’s channel fader until the test tone’s level just *below* the level of harmonic distortion (i.e., we want to only see a pure tone at 1kHz on the FFT scope)
g) Read off the SPL. This is the maximum output of the system (in dB SPL) before distortion.
h) Enter this level into the variable caldB located in ‘MaskerGUI.m’ and ‘calibration_script.m’

***Note: Using ER2 headphones passed through the TDT headphone buffer, the max output was calibrated to 93.4 dB SPL (V = -4.69). The corresponding Lynx settings for are stored in MaskingGUI_calibration_ER2.lms [MaskingGUI_calibration_Senn.lms for Sennheisers].***

***Running:***

1) If not already running, run MaskingGUI.m
2) Input subject’s name: *Subject > New subject <*Ctrl+N>. Click *Done.*
3) Select stimulus parameters: *Stimulus Parameters > Define <*Ctrl+P>.
4) Modify parameters as desired by typing values in the boxes. Turning on/off flags activates/deactivates certain modules (e.g., precursor, notched noise). To save a set of parameters: *File > Save.* This parameter file can be loaded into the GUI with *File > Load. (Note: unlike the results file, parameter files can be overwritten!)*
5) After a desired set of params are input to the window you can save this set as a stimulus block. Click the “+” in the *Blocks* section of the window to store the block; click “-“ to remove a block.

If you don’t store any blocks the GUI will use what is currently in the window. However, to ensure proper functionality, please save even a single block (i.e., the # of stored blocks should read at least 1).

To measure an unmasked (quiet) signal threshold, select “*Measure Target Alone”* checkbox.

Click the *CONFIRM* button when done.

6) The GUI then plots the stimulus waveform in the main window. (*Note: this is just a schematic and does not reflect the actual levels of the stimulus*). You can plot its spectrogram (*Plot Spectrogram*) or play the example (*Play example*) by clicking the appropriate checkboxes.

*(Note: for multiple saved blocks, only the final stimulus block is plotted)*

7) Run the experiment: *RUN > GO! <*Ctrl+R*>.* Click *“START*” to begin running the first block. The progress is printed to the command line as the adaptive tracking procedure unfolds.

Once threshold is reached, the GUI will pause. You can then plot the subject’s response track. The threshold (Ɵ) and standard deviation (σ) are also printed to the command line.

Hit “*START”* to begin the next block. When all blocks are complete, a “*Close”* option will appear in the response window and the “*START”* button will be deactivated.

Results including the stimulus parameters, response track, reversal, threshold, and standard deviation are automatically saved to a file [*subjectName_date.txt*] located in the current MATLAB directory. Each block is saved (along with its corresponding parameters) separately. Data is appended to the same file; nothing is overwritten. You can also check to see where MATLAB is saving to by typing pwd in the command line.

***Hard coded parameters not directly modifiable while running the GUI:***

- **Noise masker:** bandwidth is 80-1000 Hz. This is hard coded but can be altered in the “Hard coded parameters” section of *MaskingGUI.m*
- **High pass masker:** cutoff frequency is set in the primary function (*MaskingGUI.m*) in normalized units (e.g., 1.2* target frequency). It’s spectrum level is also set in the primary function (e.g., -50 dB/Hz below the target’s level) and it is varied adaptively with the target. To change this to another value, update line ~109 in *MaskerGUI.m* and line ~51 of *runExperiment.m* (located in the Run folder).
- **Reversals, initial/final step size, sample rate (Fs), bit depth,** and **interstimulus interval (ISI)** can be modified from their hard coded value in the “Hard coded parameters” section of *MaskerGUI.m*
- By default, the number of reversals is set at 12. Threshold is computed from the last 8. The initial step size is 5 dB and lowers to 2 dB after the first 3 reversals (this can be changed on line ~35 of *runExperiment.m*).
- A major workhorse file is *makeStim.m*. If you have a question about how a stimulus is generated this is where to find it. Others are *doTrial.m* and *runExperiment.m* which are responsible for running a single trial of the experiment and the whole block, respectively. The tracking, for example, is implemented in *runExperiment.m.*
